# Patient-reported measures in children and adolescents with chronic kidney disease and their validation: A scoping review

**DOI:** 10.1007/s00467-026-07266-x

**Published:** 2026-04-23

**Authors:** Angela Rejuso, Hassaan Asif, Martin Howell, Chandana Guha, Siah Kim, Allison Jaure, Anastasia Hughes, Kristy Nicholl, Kylie-Ann Mallit, Farzaneh Bouroumad, Jonathan Craig, Armando Teixeira-Pinto, Anna Francis, Nicholas Larkins, Anita van Zwieten, Germaine Wong

**Affiliations:** 1 Centre for Kidney Research, The University of Sydney, The Children’s Hospital at Westmead, Westmead, NSW 2145 Australia; 2 Sydney School of Public Health, The University of Sydney, Camperdown, NSW Australia; 3 Westmead Hospital, Westmead, NSW Australia; 4 Westmead Children’s Hospital, Westmead, NSW Australia; 5 Flinders University, Bedford Park, SA Australia; 6 Queensland Children’s Hospital, Brisbane, QLD Australia; 7 Department of Nephrology, Perth Children’s Hospital, Nedlands, WA Australia; 8 Nutrition and Health Innovation Research Institute, Edith Cowan University, Perth, WA Australia; 9 Medical School, University of Western Australia, Perth, WA Australia; 10 Leeder Centre for Health Policy Economics and Data, The University of Sydney, Camperdown, NSW Australia

**Keywords:** Chronic kidney disease, Health outcomes, Patient-reported measures, Pediatric

## Abstract

**Context:**

Children with chronic kidney disease (CKD) experience complex challenges affecting their physical, emotional and quality of life outcomes. Patient-reported outcome measures (PROMs) and experience measures (PREMs), collectively known as patient-reported measures (PRMs) are essential to capture these impacts, but their validation and suitability in pediatric CKD research remain underexplored.

**Objective:**

To identify and evaluate the psychometric properties of PRMs used in research in children with CKD.

**Data sources:**

Medline, Embase, CINAHL and PSYCinfo searched from inception to March 2025.

**Study selection:**

Studies that reported the results of at least one PRM completed by patients or proxies in children aged 0–18 years with CKD were included.

**Data extraction:**

Study and measure characteristics and psychometric properties of PRMs were extracted.

**Results:**

One hundred seventy-five studies involving 21,423 children from 39 countries were eligible and included. 307 PRMs were identified, representing 121 unique tools across 23 outcome domains. Pediatric Quality of Life Inventory (PedsQL) was the most frequently used measure (50%). Approximately 40% were applied in the pediatric CKD population without evidence of external validation. Internal consistency was the most frequently reported psychometric property, assessed for 19 of all identified PRMs (6%). Disease-specific instruments (PRO-KID, TECAVNER) had good internal consistency (α ≥ 0.8).

**Conclusions:**

There is firm reliance on using generic PRMs in children with CKD; however, most lack robust external validation in this population. Co-development and comprehensive validation of disease-specific instruments that reflect outcomes valued by patients and families are required to enhance the relevance of outcome assessment in pediatric CKD care.

**Graphical Abstract:**

A higher-resolution version of the Graphical abstract is available as  Supplementary information

**Figure:**
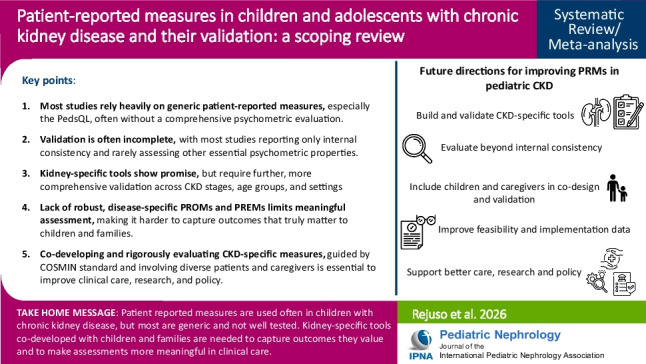

**Supplementary Information:**

The online version contains supplementary material available at 10.1007/s00467-026-07266-x.

## Introduction

Childhood chronic kidney disease (CKD) carries a significant disease and treatment burden. Children with CKD experience a complex range of co-morbidities, including cardiovascular disease, infections, cognitive impairment, psychosocial challenges, and growth and developmental issues [1–8]. Children with advanced CKD on dialysis or post-transplant face complex treatment regimens and endure a high symptom burden, including fatigue, pain, psychological distress, and nausea, which adversely impacts their quality of life (QoL) and well-being [1, 9, 10].

Patient-reported measures (PRMs), comprising patient-reported outcome measures (PROMs) and patient-reported experience measures (PREMs), capture patients’ perspectives on symptoms, QoL, experiences of care, and the broader impact of the disease in their daily life [11, 12]. PROMs collect patients’ perceptions of how they feel and function, while PREMs elicit patients' experiences of treatment [11]. These instruments enable clinicians and researchers to tailor interventions to individuals’ needs, track symptom trajectories and evaluate the effectiveness of potential therapeutic approaches [13, 14]. Standard instruments commonly used to assess the health-related quality of life (HRQoL) in children include the generic Pediatric Quality of Life Inventory (PedsQL), KIDSCREEN, and Pediatric Patient-Reported Outcomes Measurement Information System (PROMIS) measures of physical, mental, and social health. While these instruments are widely adopted in children with CKD, their validity, reliability, and overall psychometric robustness in this population remain uncertain [15–23]. Given the unique needs and priorities of children with CKD, generic measures may fail to capture key issues such as fatigue, growth concerns, dietary restrictions, and the burden associated with dialysis or transplant care. Thus, identifying valid and reliable disease-specific PRMs is crucial to capture outcomes that accurately reflect the lived experience of children with CKD and their caregivers [15, 23–25]. A comprehensive understanding of these measures is needed to ensure valid and reliable outcome and experience assessment in research and clinical practice while minimizing respondent burden by limiting assessment to the most robust tools. This scoping review aimed to systematically identify and synthesize the scope of PRMs used in children and adolescents with CKD and to evaluate their psychometric properties.

## Methods

This scoping review was conducted in accordance with the Joanna Briggs Institute methodology for scoping reviews and followed the Preferred Reporting Items for Systematic Reviews and Meta-Analyses—Extension for Scoping Reviews (PRISMA-ScR) checklist (Supplementary Table S1) [26, 27]. A protocol was developed before the review to guide the review process (available from the authors upon request).

### Search strategy

We searched MEDLINE, Embase, PsycINFO and CINAHL from inception to March 2025 with no restrictions on the year or language of publication. The search strategy is provided in the Supplementary Table S2.

### Study selection process

Titles and abstracts were independently screened against the inclusion/exclusion criteria by at least two authors (of A.R, H.A, K.N and A.H). Full-text articles were independently double-screened for 10% of the studies by two reviewers (of A.R, H.A, A.H, and M.H). The remaining full texts were screened by one reviewer. For any uncertainties or disagreements that could not be resolved, a third reviewer was consulted (A.vZ).

### Study inclusion/exclusion criteria

We included quantitative studies (cohort, case–control or cross-sectional studies and randomized controlled trials) and qualitative studies, which described the use of a patient reported measure (PRM) (including PROMs and PREMs) or their development and validation in children or adolescents aged 0–18 years with any stage of CKD (dialysis, kidney transplant or CKD stages 1–5 not yet receiving kidney replacement therapy (KRT)). The PRM had to be completed by the child and/or their proxy (e.g. a parent or caregiver) to be deemed eligible. For PROMs, the proxy report needed to pertain to the child’s health or experiences, rather than those of the proxy. For PREMs, we included both proxy reports of the child’s experience and the proxy’s own experience, as both perspectives are relevant to evaluating the child’s healthcare experience. Studies were excluded if the outcomes were clinician-reported, outcomes did not include PRM, individuals over 18 years of age or mixed child/adult populations where there were no stratified child-specific data, or if the study was a conference abstract, thesis, editorial, opinion piece, case study, or case series. Reviews were excluded; however, the full texts were accessed first, and their references were reviewed for relevant studies to include. Where a study reported use but not validation of a PRM, we hand-searched the reference list and incorporated any eligible source validation studies that were cited in the study into our review if they were not already included.

### Data extraction

The following characteristics were extracted for each study by A.R: evidence source details (including author, publication year, study type, objective, country, data collection method, length of study, and setting), study population details (including demographic and clinical details of the child and demographic details of the caregiver), information on included PRMs (including the specific PRMs used, main outcome domains covered by these PRMs, domains and sub-domains assessed, response format, scoring methods, frequency of use, mode of administration, completion time, cost, and language), and details (methods and findings) of validation or assessment of psychometric properties for the PRMs within included studies.

### Psychometric properties

For studies that evaluated the psychometric properties of included PRM(s), we used the Consensus-based Standard for the selection of health Measurement Instruments (COSMIN) methodology guideline for systematic reviews of PROMs to extract and report on the psychometric properties [28] (Fig. 4 and Supplementary Table 4). Definitions of all psychometric properties, based on COSMIN standards, are available in Table 2.

### Mapping and synthesis

We developed a world map and a bar chart, showing the number of studies by country and the frequency of outcomes across all PRMs. Outcome domains were defined as follows: QoL (including HRQoL assessed through generic multi-attribute measures), behavior/mental health (including positive and negative aspects of mental health, self-perception, and mindfulness), sleep, physical activity/mobility, treatment adherence (including medication adherence and compliance), social wellbeing (including social changes, social relationships and social health), treatment experience (including experience with hospitals and clinicians, and caregiver burden/experience), symptoms, overall health/health status, diet/nutrition (including appetite), transition/coping, and others (such as fatigue, schooling, food insecurity, health behaviors/preventative health, access to treatment, experience of medical treatments in research, screen time, activities of daily living, attitude towards illness, and oral health) [29]. Characteristics and measurement properties of validated PRMs were summarized narratively and in text, with detailed information provided in the supplementary materials.

## Results

### Characteristics of included studies

**Fig. 1:**
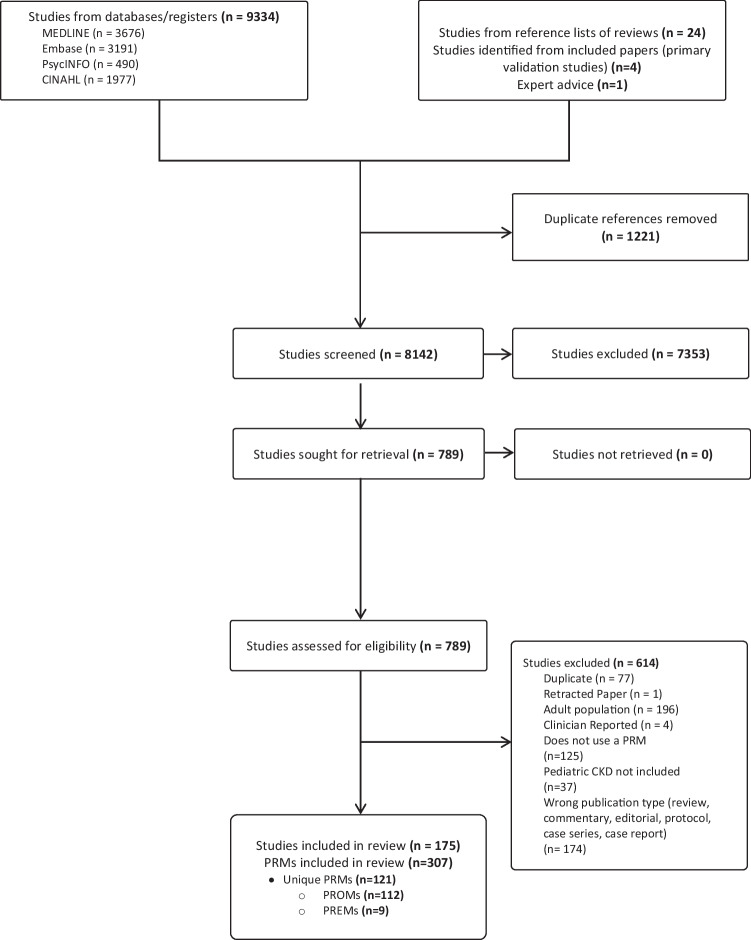
PRISMA flow diagram

The search strategy retrieved 9334 studies, with an additional 24 identified through reference lists of included reviews, and 4 primary validation papers identified from citations of included papers and 1 from expert advice. Following de-duplication, 8142 studies were screened by title and abstract, and 789 were assessed for full-text eligibility. Of these, 175 studies met the inclusion criteria and were included in this review. The study selection process is shown in Fig. 1.

**Fig. 2:**
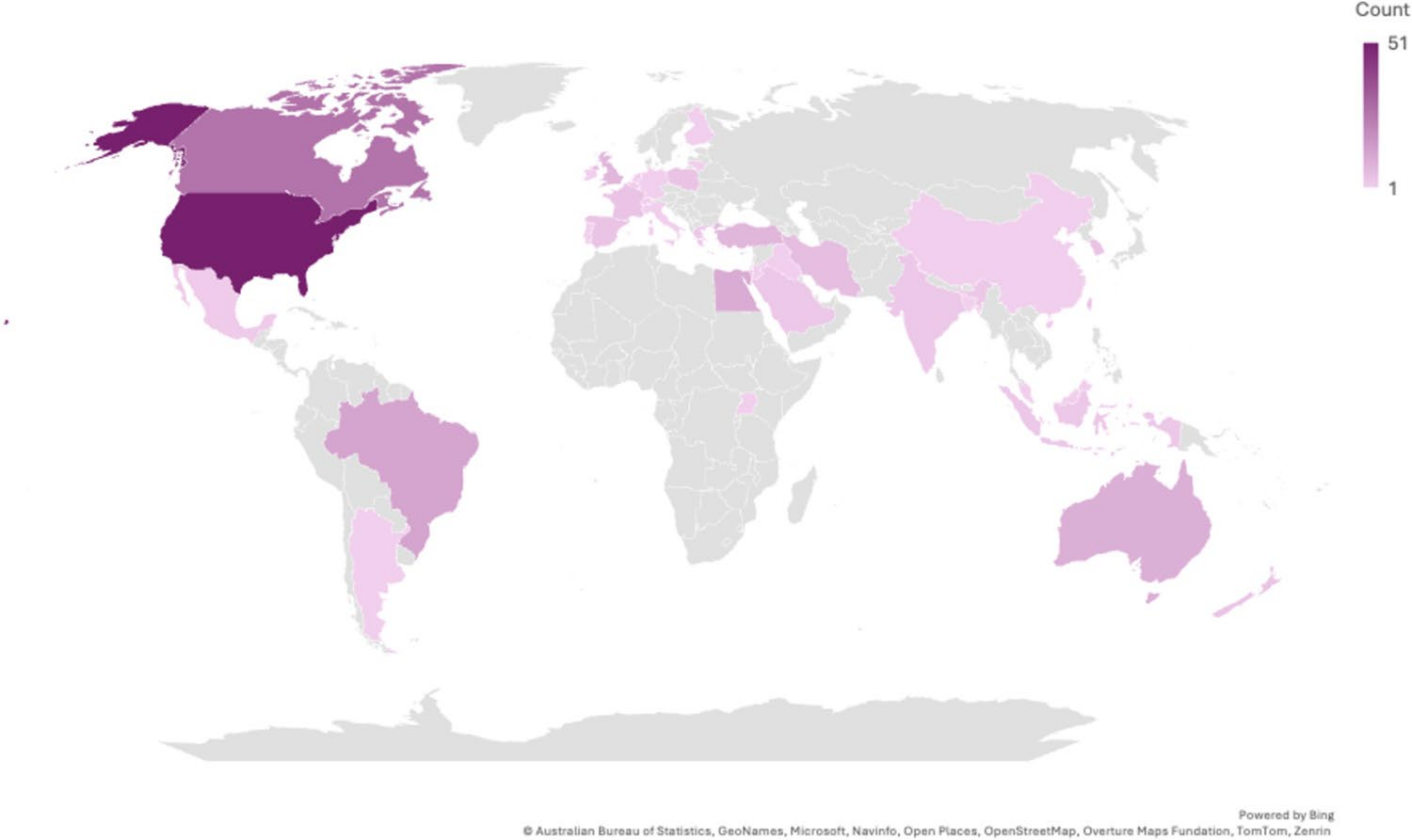
Number of included studies conducted across countries, Figure notes: Count of included studies across countries is as follows: Argentina *n* = 1, Australia *n* = 5, Australia and New Zealand *n* = 4, Bangladesh *n* = 1, Belgium *n* = 2, Brazil *n* = 13, Canada *n* = 13, China *n* = 1, Egypt *n* = 11, Finland *n* = 1, France *n* = 4, Germany *n* = 1, Greece *n* = 3, Hong Kong *n* = 1, India *n* = 3, Indonesia *n* = 3, Iran *n* = 6, Republic of Ireland *n* = 1, Iraq *n* = 1, Israel *n* = 1, Italy n = 1, Jordan *n* = 1, Lithuania *n* = 1, Malaysia *n* = 1, Mexico *n* = 2, Poland *n* = 4, Portugal *n* = 1, Saudi Arabia *n* = 3, South Korea *n* = 4, Spain *n* = 4, Switzerland *n* = 1, Taiwan *n* = 3, The Netherlands *n* = 3 Turkey *n* = 8, Uganda *n* = 1, United Kingdom *n* = 8, United states of America *n* = 37, United States of America and Canada *n* = 14

Across the included studies, 21,423 children with CKD were involved. Studies were conducted across 39 countries, with the most significant proportion conducted in the United States (37 studies, 21%), followed by combined cohorts from the United States and Canada (14, 8%), Canada alone (13, 8%), and Egypt (11, 6%). A third of the studies were conducted in low- and middle-income countries. Figure 2 illustrates the global distribution of studies. Most studies were cross-sectional (123, 70%), and 116 (66%) focused exclusively on children with CKD. The studies included children with a diverse range of CKD stages: 79 (30%) included children with CKD stages I–IV, 75 (29%) involved children treated with dialysis, 69 (26%) included transplant recipients, and 17 (7%) included patients with nephrotic syndrome. The proportion of male participants ranged from 24 to 90% across studies. The study characteristics are summarized in Tables 1 with detailed information for each survey provided in Supplementary Table .

**Table 1:** Study characteristics (*n* = 175)

Study characteristic	Value
Publication year, *n* (%)	
Before 2000	4 (2.3)
2000–2009	25 (14.3)
2010–2019	87 (49.7)
2020 onwards	59 (33.7)
Study type, *n* (%)	
Cross-sectional	123 (70.3)
Cohort	30 (17.1)
Mixed methods	7 (4.0)
RCT	6 (3.4)
Case–control	4 (2.3)
Other^a^	2 (1.1)
Qualitative	2 (1.1)
Before and after study	1 (0.6)
Sample size, *n* (%)	
0–49	79 (45.1)
50–249	75 (42.9)
250–500	12 (6.9)
500 +	9 (5.1)
Population composition, *n* (%)	
CKD population only	116 (66.3)
CKD + other populations	59 (33.7)
Child mean age, *n* (%)	
0–5	1 (0.6)
6–11	31 (17.7)
12–18	67 (38.3)
Not reported	76 (43.4)
Child CKD stage (*n*)*	
Pre-dialysis (CKD I–V)	79 (30.2)
Transplant	69 (26.3)
Dialysis (PD/HD)	75 (28.6)
Not reported	22 (8.4)
Nephrotic Syndrome	17 (6.5)
Type of patient-reported measure *n* (%)^b^	
Patient-reported outcome measures (PROMs)	112 (92.6)
Patient-reported experience measures (PREMs)	9 (7.4)
Number of PRMs used in the study *n* (%)	
1	102 (58.3)
2	41 (23.4)
3	20 (11.4)
4	5 (2.9)
5	4 (2.3)
6	3 (1.7)
PRM target age group	
Children (< 12 years)	7 (4.0)
Not reported	17 (9.7)
Adolescents (12–18 years)	23 (13.1)
Children and adolescents	128 (73.1)
PRM outcome reporter *n* (%)	
Participant and proxy	66 (37.7)
Participant only	45 (25.7)
Participant or proxy	35 (20.0)
Proxy only	24 (13.7)
Not reported	5 (2.9)
Validation of included PRM(s) in the paper *n* (%)	
No	153 (87.4)
Yes (all of the PRMs in the study)	20 (11.4)
Yes (some of the PRMs in the study)	2 (1.1)

^*^ Note: Total percentages exceed 100 as some studies fall into multiple categories

^a^ Other includes 1 non-randomized clinical trial and 1 quasi-experimental study

^b^ There were 307 PRMs used in total, however only 121 were unique

Abbreviations: *CKD*, Chronic kidney disease; *HD*, Hemodialysis; *PD*, Peritoneal dialysis; *PRM*, Patient reported measure; *PREM*, Patient reported experience measure; *PROM*, Patient reported outcome measure; *RCT*, Randomized controlled trial

**Table 2:** Psychometric properties definitions per the COSMIN guidelines

Measurement	Definitions as per the COSMIN guidelines [56]
Reliability	“The degree to which the measurement is free from measurement error”
Reliability (including test–retest, inter-rater, and intra-rater)	“The extent to which scores for patients who have not changed are the same for repeated measurement under several conditions: e.g. using different sets of items from the same health related-patient reported outcomes (HR-PRO) (internal consistency); over time (test–retest); by different person on the same occasion (inter-rater); or by the same persons (i.e. rater of responders) on different occasions (intra-rater)"
Internal consistency	“The degree of inter-relatedness among the items”
Measurement error	“The systematic and random error of a patient’s score that is not attributed to true changes in the construct to be measured”
Content validity (including face validity)	“The degree to which the content/items of a HR-PRO instrument is an adequate reflection of the construct to be measured”
Construct validity	“The degree to which the scores of a HR-PRO instrument are consistent with hypotheses (with regard to internal relationships, relationships to scores of other instruments, or differences between relevant groups) based on the assumption that the HR-PRO validly measures the construct to be measured”
Structural validity	“The degree to which the scores of a HR-PRO instrument are an adequate reflection of the dimensionality of the construct to be measured”
Hypotheses testing	“Definition as per construct validity above”
Cross-cultural validity	“The degree to which the performance of the items on a translated or culturally adapted HR-PRO instrument are an adequate reflection of the performance of the items of the original version of the HR-PRO instrument”
Criterion validity	“The degree to which the scores of a HR-PRO are an adequate reflection of ‘gold standard’”
Responsiveness	“The ability of a HR-PRO instrument to detect change over time in the construct to be measured”

*HR-PRO*, Health-related patient reported outcome

### Characteristics of PRMs: an overview

**Fig. 3:**
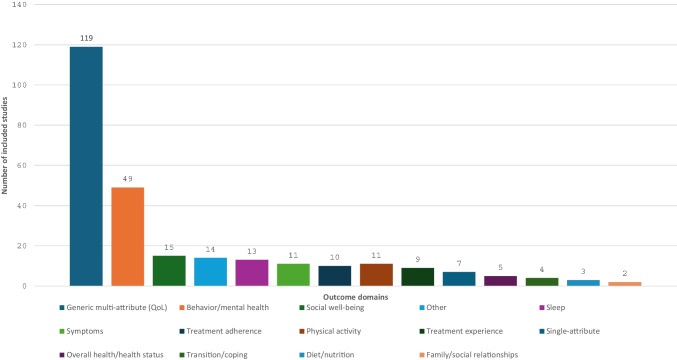
Number of included studies assessing different outcome domain, Figure notes: Count of PRM outcome domains covered in included studies: Generic multi-attribute measures (assessing QoL) *n* = 119, behavior/mental health *n* = 49, social well-being *n* = 15, other *n* = 14 (including fatigue *n* = 4, access to treatment *n* = 1, activities of daily living *n* = 1, attitude towards illness *n* = 1, research experience *n* = 1, schooling *n* = 1, food insecurity *n* = 1, experience of medical treatments in research *n* = 1, health behaviors/preventative health *n* = 1, oral health *n* = 1, mobility *n* = 1), sleep *n* = 13, symptoms *n* = 11, physical activity *n* = 11, treatment adherence *n* = 10, treatment experience *n* = 9, single-attribute measures *n* = 7, overall health/health status *n* = 4, diet/nutrition (including appetite) *n* = 4, transition/coping *n* = 4, fatigue *n* = 2, food insecurity *n* = 1, schooling *n* = 1, oral health *n* = 1, family/social relationships *n* = 2. Note: Count does not sum to total N of included studies as some studies covered multiple domains or to total number of PRMs as some studies included multiple PROMs for one domain

QoL was the most frequently assessed outcome domain, being reported in 119 studies, followed by behavior/mental health in 49 studies and social well-being in 15 studies. Figure 3 presents further details on the distribution of the outcome domains across all studies. Across the 175 included studies, 102 (59%) used a single PRM measure, while 42 (26%) used two, and smaller proportions used three measures (15%) or more. Outcomes were most commonly reported by patients and their proxies (66 studies, 38%), followed by patient-only reports (45 studies, 26%), patient or proxy reports (35 studies, 20%), and proxy-only reports (24 studies, 14%). Majority of PRMs used in studies had a target age group of children and adolescents (128 studies, 73%). There were very few studies which reported PRM implementation characteristics, with zero studies reporting readability and 35 studies (20%) reporting time to administer. Most (153 studies, 87%) did not conduct any form of validation of the PRMs being used within their study population, and only 10 studies (6%) explicitly stated that they aimed to perform external validation.

A total of 121 unique PRMs were identified across the 175 studies. Of these, 112 were PROMs and nine were PREMs. The following sections outline the characteristics and psychometric properties of PROMs and PREMs respectively.

### Patient-reported outcome measures (PROMs)

#### Characteristics of PROMs

A total of 112 unique PROMs were identified in our review. Of these, 102 were generic (not disease-specific), four were preference-based measures, and six were disease-specific for CKD. In terms of development source, five were author-developed or adapted PRMs, while the remaining 107 were pre-existing instruments.

The Pediatric Quality of Life Inventory (PedsQL) was the most frequently used PROM (94, 32%). Within these 94 instances, several different modules were used, including the 4.0 generic core scale (58, 62%), 3.0 End-stage renal disease (ESRD) module (19, 20%), transplant module (10, 11%), family information form (3, 3%), oral health scale (1, 1%), and multidimensional fatigue scale (1, 1%). Other commonly used measures included the Child Depression Inventory (CDI; 10, 3%), Child Behavior Checklist (CBCL; 10, 3%), PROMIS instruments (9, 3%), Pediatric Sleep Questionnaire (PSQ; 7, 2%), and Physical Activity Questionnaire (PAQ; 7, 2%).

#### Psychometric properties of PROMs by outcome domain

**Fig. 4:**
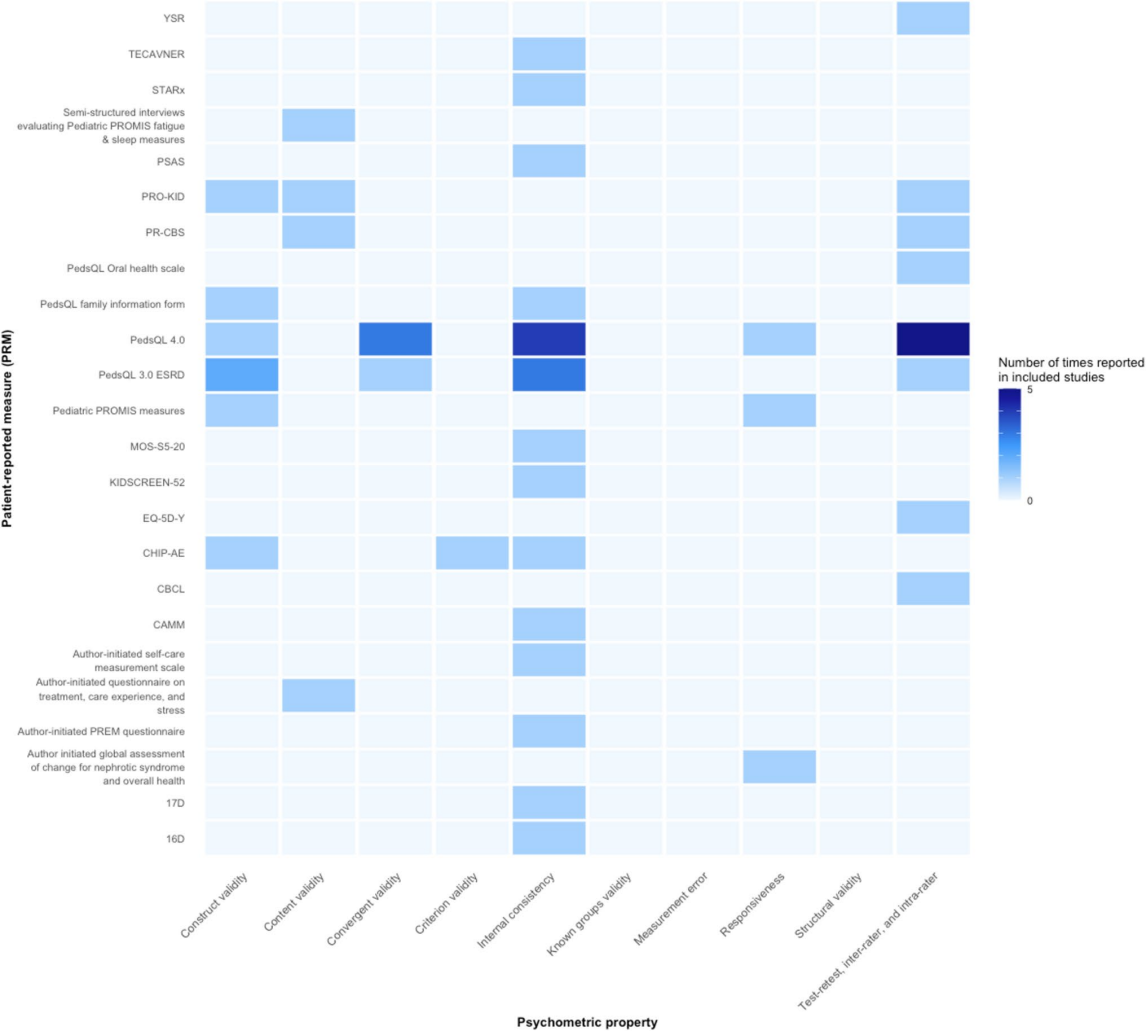
Heatmap of psychometric properties of PRMs validated in included studies, Figure notes: Abbreviations: CBCL = child behavior checklist, CHIP-AE = Child Health and Illness Profile—Adolescent Edition, EQ-5D-Y = EuroQol Five Dimensions Youth version, ESRD = End-stage renal, disease, MOS-SF-20 = 20-item Medical Outcomes Study, PedsQL = Pediatric quality of life inventory, PR-CBS = Pediatric Renal Caregiver Burden Scale, PROMIS = Patient-Reported Outcomes, Measurement Information System, PRM = patient-reported measures, QoL = Quality of Life, PSAS = Patient Self-Advocacy Scale, TECAVNER = Test of Quality of Life in Children with Kidney Disease, YSR = youth self-report

Figure 4 and Supplementary Table 4 present a summary of the psychometric properties reported in the included studies across outcome domains and measures. Across the 19 studies which performed PROM validation, 1685 children were included across 9 different countries. Internal consistency was the most frequently assessed reliability property across all measures, followed by other forms of reliability including test–retest, inter-rater, and intra-rater reliability. Construct validity and responsiveness were the least commonly evaluated. Most studies reported one or two psychometric properties, with none assessing all properties in a single study.

#### Quality of life (QoL)

QoL was the outcome domain with the most comprehensive evidence on psychometric properties, assessed through generic multi-attribute instruments encompassing multiple domains of health and well-being, including 21 PROMs, 15 of which were versions of the PedsQL. Notably, most of these psychometric evaluations related to the PedsQL, highlighting its robust reliability across various versions, including translated adaptations and condition-specific modules. Internal consistency was the most frequently reported psychometric property for 13 of these HRQoL PROMs [30–39]. Of these, 16 studies reported Cronbach’s alpha values, with the majority (*n* = 14) reporting values > 0.7, reflecting good to excellent reliability [30–36, 38, 39]. Inter-rater and test–retest reliability was reported for six instruments, with intraclass correlation coefficients (ICCs) ranging from 0.47 to > 0.9 and agreement varying by age and domain, indicating poor to excellent reliability [38, 40–43]. Construct validity was supported in four PedsQL instruments through significant intercorrelations with symptom burden measures and other validated scales [36–38, 44]. Known-groups validity was assessed in two PROMs, with the PedsQL 4.0 distinguishing between children with CKD and healthy peers, and the PedsQL 3.0 ESRD module showing moderate to strong construct validity [34].

The Test of Quality of Life in Children with kidney disease (TECAVNER), was developed explicitly in Spanish for pediatric CKD populations [45]. The tool demonstrated good reliability in child and parent proxy reports (α = 0.925 for child; 0.916 for parent) and internal consistency, with values exceeding α ≥ 0.7 across all domains [45].

#### Health status/symptoms

Three instruments focused on health status or symptom burden with validation information (Child Health and Illness Profile – Adolescent Edition [CHIP-AE], PRO-Kid, and author-initiated global assessment of change for nephrotic syndrome and overall health [GAC]). All reported high internal consistency (α ≥ 0.80) [37, 44, 46]. Construct validity was reported in the CHIP-AE and PRO-Kid, with both instruments identifying differences across CKD treatment stages [37, 46]. Responsiveness was assessed for the GAC using the Patient-reported Outcomes Measurement Information System (PROMIS) Pediatric measures, which were administered via computer-adaptive testing and were able to detect clinically meaningful changes in health status over time [44]. In addition, PRO-Kid, the first PROM specifically developed to assess symptom frequency and burden in pediatric CKD, demonstrated strong psychometric performance [37, 47]. Evidence indicated strong internal consistency with α of 0.83 and 0.84 for frequency and burden, respectively, and moderate to strong correlations with PedsQL scores in the expected direction, providing preliminary support for construct validity [37].

#### Behavior and mental health

Three behavioral and mental health instruments (Child Behavior Checklist (CBCL), Youth self-report (YSR), Child and Adolescent Mindfulness Measure (CAMM)) were validated in the included studies [30, 48]. All reported strong internal consistency (α ≥ 0.80), but none assessed other psychometric domains [30, 48]. The CBCL and YSR reported consistent α values across parent and self-report versions, reflecting strong internal consistency and reliability across respondents [30].

#### Other domains

Validation data were also available in included studies for several PROMs across other outcome domains, including perceived oral health, self-care, and self-advocacy, with validation often limited to internal consistency [31, 42, 44, 48, 49]. Four study-specific measures with validation data were identified, covering a range of domains such as hospital and clinical staff experience, self-care behaviors, caregiver burden, and healthcare management. While most demonstrated moderate to high internal consistency (α = 0.77–0.94), few evaluated psychometric properties beyond internal consistency [31, 33, 49, 50].

### Patient-Reported Experience Measures (PREMs)

#### Characteristics of PREMs

Our review found a small number of PREMs relative to PROMS, with only nine identified. Of these nine PREMs, six were author-initiated, two were generic, and one was disease-specific. The focus of these PREMs were caregiver burden, healthcare experiences, staff/clinical team interactions.

#### Psychometric properties of PREMs

Validation was limited for PREMs. Only three studies performed validation, involving 228 children across 3 countries. Validation data for PREMs were available in outcome domains related to caregiver burden and healthcare experiences, with validation limited to internal consistency, reliability, and content validity [33, 50, 51]. Notably, the Pediatric Renal Caregiver Burden Scale (PR-CBS), a proxy-reported PREM designed to assess caregiver burden in pediatric CKD populations, demonstrated acceptable internal consistency and preliminary reliability in a test–retest analysis [51].

## Discussion

In this review of 175 studies using 121 unique instruments in children with CKD, we found a firm reliance on generic PRMs. Most PRMs applied in the pediatric CKD cohorts lacked external validation. QoL was the most frequently assessed outcome domain, with the PedsQL being the most commonly used instrument. Many pediatric CKD studies using these tools did not report or provide sufficient CKD-specific evidence for key psychometric properties or assessed only a narrow subset of properties. Internal consistency was the most frequently reported psychometric property, with few assessments of other domains, such as structural validity, criterion validity, responsiveness, or test–retest reliability. Notably, no single PRM was evaluated across all COSMIN psychometric properties.

Our study findings align with previous reviews of PRMs conducted in adult and pediatric populations with chronic diseases. A recurring theme is the predominant use of generic instruments, particularly the PedsQL in children, often used without disease-specific validation. A review of PROMs used in children and adolescents in palliative care identified that the PedsQL was used in nearly half of the studies (50%), with limited evidence of comprehensive psychometric evaluation within this population [52]. This raises concerns about the routine use of non-validated or partially validated tools in pediatric populations. A recent review of multi-attribute utility instruments (MAUI) in children reported that no generic tools met psychometric standards across all evaluated domains [53]. A review of frequently used generic PROMs in children demonstrated that no instrument, including the PedsQL, met all International Society for Pharmacoeconomics and Outcomes Research (ISPOR) recommended good research practices [54].

Previous reviews of specific outcome domains in pediatric CKD have also highlighted similar concerns. A recent review identified 63 instruments used to assess life participation in children with CKD, with the PedsQL 4.0 employed in almost half of the studies (48%), followed by the PedsQL 3.0 ESRD module (9%) [55]. Only 30% of instruments had undergone any psychometric validation in children with CKD. However, most were not designed to evaluate life participation as a primary outcome. In our review, while PedsQL demonstrated strong internal consistency across multiple studies, evidence supporting other key measurement properties, such as content validity, construct validity, and responsiveness, is lacking. There is also limited evidence supporting responsiveness and known-groups validity [56–58]. These gaps are concerning, given the complexity of childhood CKD and the need to ensure that this generic instrument can accurately capture outcomes important to patients and families with CKD, including treatment demands, fatigue, growth concerns, and psychosocial well-being [24, 59–62].

PROMs specific to pediatric CKD included the PedsQL 3.0 ESRD module, the Test of Quality of Life in Children with Kidney Disease (TECAVNER), and PRO-Kid [36, 45, 47]. The PedsQL 3.0 ESRD module is a disease-specific module designed to assess treatment-related burden, disease-specific worry, communication challenges, impact on daily and peer relationships, and perceived physical appearance, providing a more tailored assessment of QoL in children with kidney failure [36, 63]. However, evidence for key psychometric properties beyond internal consistency, reliability (inter-rater reliability), and known-groups validity is limited. The TECAVNER provides a comprehensive evaluation of CKD-specific HRQoL domains, however, current validation evidence is currently confined to internal consistency [45]. PRO-Kid is the first PROM to measure symptom frequency and burden within the pediatric CKD population [37, 47]. Its rigorous psychometric development and assessment emphasized the need for more disease-specific instruments that capture the unique experiences and challenges faced by children with CKD [37, 47]. Despite promising early evidence, further validation is required to establish responsiveness, interpretability, and applicability across CKD stages, age groups, and care settings. Collectively, these CKD-specific PROMs reported potential utility of these instruments in children with CKD, but further psychometric testing is required to establish feasibility, implementation, and broader applicability, alongside further comprehensive assessment across COSMIN measurement domains. Similarly, validation data for PREMS was sparse, and their use in pediatric CKD remains limited with very few instruments undergoing rigorous psychometric evaluation, highlighting a research and clinical gap. Management of pediatric CKD is highly resource-intensive and service-driven, particularly during dialysis and transplant, and requires sustained, long-term engagement with multiple health services [61, 64, 65]. Without robust, disease-specific PREMs, it is challenging to assess the quality, accessibility, and responsiveness of service provision from the patient's and caregiver's perspective, with potential implications for decision-making in clinical practice and policy [66]. Among the few disease-specific PREMs identified, the Pediatric Renal Caregiver Burden Scale (PR-CBS) captures caregiver stress related to disease progression, hospital experiences, and the complexity of care [51]. While there is not a single superior pediatric CKD PROM or PREM to use, the choice should be guided by the clinical context, CKD stage, age group, respondent, and cultural and language considerations, alongside the specific domains of interest.

Adopting a patient-centered approach to the design and validation of these measures is important to ensure strong communication between patients, families, and clinicians, facilitate shared decision-making, and enable the delivery of appropriate tailored interventions. The Standardized Outcomes in Nephrology—Children and Adolescents (SONG-Kids) initiative has also highlighted the need for validated, condition-specific tools to measure core outcomes such as life participation, survival, kidney function, and infection which are important to patients, caregivers and clinicians [24, 25]. Integrating social determinants of health into PRM development and validation may also further enhance relevance, equity, and interpretability, as very few studies assessed how sociodemographic context influences PRM performance and results. Future work should explicitly evaluate measurement properties across diverse populations to support equitable use.

Beyond psychometric performance, our review highlights several important gaps in the feasibility, implementation, and coverage across age groups which warrant consideration in future pediatric CKD PRM research. There were very few studies which reported PRM implementation characteristics such as readability and time to administer despite their importance for clinical use. Several outcome domains were limited across specific age-respondent groups. Evaluation of clinical efficiency and downstream clinical and policy impacts were beyond the primary scope of this scoping review. However, these findings highlight important priorities for future research and reviews on the feasibility, acceptability and implementation characteristics, in conjunction to psychometric robustness of PRMs. The use of tools with limited or unproven validation may also lead to inaccurate interpretation of patient experience, with implications for clinical decision-making and health policy. The use of validated, age-appropriate assessment tools, together with the routine involvement of allied health professionals, and care navigators, may facilitate systematic identification of patients’ and caregivers’ needs and better capture the complexity of their experiences.

Our review has several strengths. The review provides a valuable reference for researchers, clinicians, and healthcare leaders seeking to implement rigorous patient and caregiver-centered outcome assessment in pediatric CKD. We searched multiple key health and medical databases across disciplines, without language or date restrictions, to ensure a comprehensive capture of relevant studies. We also identified studies from reviews and primary validation studies cited in included studies using PRMs. The study selection process was conducted independently by at least two reviewers, contributing to a systematic and transparent review process. The application of established COSMIN criteria provides a systematic and transparent appraisal of validity and reliability evidence, supporting the identification of clinically meaningful and methodologically robust instruments. However, our review has several limitations. Firstly, validation information was only collected from studies that explicitly reported psychometric properties, which may have led to an underestimation of the extent of validation evidence available. Secondly, we did not include grey literature, which may have resulted in the omission of relevant instruments or validation data. We were unable to meta-analyze the point estimates of the individual studies as the data was highly heterogeneous, with variations in the study populations, outcome definitions, and statistical approaches that precluded meaningful and robust quantitative synthesis.

## Conclusion

This scoping review identified a heavy reliance on the use and validation of generic PRMs in pediatric CKD, particularly the PedsQL, often without a comprehensive psychometric evaluation. Few studies have validated the PRMs appropriately for this population, with most focusing solely on internal validity while neglecting other key properties. Condition-specific tools such as PRO-Kid, PR-CBS, and TECAVNER show promise but require further validation. Rigorous development and evaluation of pediatric CKD-specific instruments, guided by COSMIN standards and co-developed with patients and caregivers, are essential to ensure the PRMs used in pediatric CKD are robust, relevant, and capable of informing care, research, and policy. Strengthening these tools will require early and meaningful engagement with diverse and representative groups of children and caregivers to gain insight into their varied lived experiences, and help in refining and assessing the relevance, acceptability, and performance of the PRMs.

## Supplementary Information

Below is the link to the electronic supplementary material.

Graphical abstract (PPTX 271 KB)

Supplementary file2 (DOCX 565 KB)

## Data Availability

All data supporting the findings of this study are available within the paper and its Supplementary Information.
