# Supplementary material for: Patient-reported measures in children and adolescents with chronic kidney disease and their validation: A scoping review
Source: Pediatr Nephrol. 2026 Apr 23;41(10):3347–59. doi: 10.1007/s00467-026-07266-x (PMC13481412; doi:10.1007/s00467-026-07266-x)
Supplement: Supplementary file 1 [file 467_2026_7266_MOESM2_ESM.docx]

**SUPPLEMENTARY MATERIAL FOR PATIENT-REPORTED MEASURES IN CHILDREN AND ADOLESCENTS WITH CHRONIC KIDNEY DISEASE AND THEIR VALIDATION: A SCOPING REVIEW**

**Published in Pediatric nephrology**

**Corresponding author:** Angela Rejuso, Centre for Kidney Research, The Children’s Hospital at Westmead, Westmead NSW 2145, Sydney, Australia. E-mail: angela.rejuso@sydney.edu.au ORCID ID: <https://orcid.org/0009-0008-7253-5449>

**Contents:**

Supplementary table SI. Preferred Reporting Items for Systematic Reviews and Meta-Analyses—Extension for Scoping Reviews (PRISMA-ScR) checklist

Supplementary table SII. Table of search strategies

Supplementary table SIII. Characteristics of included studies

Supplementary table SIV. Psychometric properties definitions per the COSMIN guidelines

Supplementary table SV. Detailed summary of psychometric properties of PRMs used in the included studies

**S1. Preferred Reporting Items for Systematic Reviews and Meta-Analyses—Extension for Scoping Reviews (PRISMA-ScR) checklist**

| **SECTION** | **ITEM** | **PRISMA-ScR CHECKLIST ITEM** | **REPORTED ON PAGE #** |
| --- | --- | --- | --- |
| **TITLE** | | | |
| Title | 1 | Identify the report as a scoping review. | 1 |
| **ABSTRACT** | | | |
| Structured summary | 2 | Provide a structured summary that includes (as applicable): background, objectives, eligibility criteria, sources of evidence, charting methods, results, and conclusions that relate to the review questions and objectives. | 2 |
| **INTRODUCTION** | | | |
| Rationale | 3 | Describe the rationale for the review in the context of what is already known. Explain why the review questions/objectives lend themselves to a scoping review approach. | 4 |
| Objectives | 4 | Provide an explicit statement of the questions and objectives being addressed with reference to their key elements (e.g., population or participants, concepts, and context) or other relevant key elements used to conceptualize the review questions and/or objectives. | 4 |
| **METHODS** | | | |
| Protocol and registration | 5 | Indicate whether a review protocol exists; state if and where it can be accessed (e.g., a Web address); and if available, provide registration information, including the registration number. | 4 |
| Eligibility criteria | 6 | Specify characteristics of the sources of evidence used as eligibility criteria (e.g., years considered, language, and publication status), and provide a rationale. | 5 |
| Information sources* | 7 | Describe all information sources in the search (e.g., databases with dates of coverage and contact with authors to identify additional sources), as well as the date the most recent search was executed. | 4 |
| Search | 8 | Present the full electronic search strategy for at least 1 database, including any limits used, such that it could be repeated. | Supplementary File; page 4 to 9 |
| Selection of sources of evidence† | 9 | State the process for selecting sources of evidence (i.e., screening and eligibility) included in the scoping review. | 4 to 5 |
| Data charting process‡ | 10 | Describe the methods of charting data from the included sources of evidence (e.g., calibrated forms or forms that have been tested by the team before their use, and whether data charting was done independently or in duplicate) and any processes for obtaining and confirming data from investigators. | 5 to 6 |
| Data items | 11 | List and define all variables for which data were sought and any assumptions and simplifications made. | 6 |
| Critical appraisal of individual sources of evidence§ | 12 | If done, provide a rationale for conducting a critical appraisal of included sources of evidence; describe the methods used and how this information was used in any data synthesis (if appropriate). | N/A |
| Synthesis of results | 13 | Describe the methods of handling and summarizing the data that were charted. | 7 |
| **RESULTS** | | | |
| Selection of sources of evidence | 14 | Give numbers of sources of evidence screened, assessed for eligibility, and included in the review, with reasons for exclusions at each stage, ideally using a flow diagram. | 22 to 23 |
| Characteristics of sources of evidence | 15 | For each source of evidence, present characteristics for which data were charted and provide the citations. | Supplementary file; page 10 to 34 |
| Critical appraisal within sources of evidence | 16 | If done, present data on critical appraisal of included sources of evidence (see item 12). | N/A |
| Results of individual sources of evidence | 17 | For each included source of evidence, present the relevant data that were charted that relate to the review questions and objectives. | Supplementary file; page 10 to 34 |
| Synthesis of results | 18 | Summarize and/or present the charting results as they relate to the review questions and objectives. | 6 to 12 |
| **DISCUSSION** | | | |
| Summary of evidence | 19 | Summarize the main results (including an overview of concepts, themes, and types of evidence available), link to the review questions and objectives, and consider the relevance to key groups. | 12 to 16 |
| Limitations | 20 | Discuss the limitations of the scoping review process. | 15 to 16 |
| Conclusions | 21 | Provide a general interpretation of the results with respect to the review questions and objectives, as well as potential implications and/or next steps. | 16 |
| **FUNDING** | | | |
| Funding | 22 | Describe sources of funding for the included sources of evidence, as well as sources of funding for the scoping review. Describe the role of the funders of the scoping review. | N/A |

| **S2. Table of search strategies** | |
| --- | --- |
| **Medline Ovid search strategy (inception to 01/06/2023, updated 30/09/2024 and 01/03/2025)**   1. exp child/ or exp Adolescent/ 2. (child* or adolescen* or teenager* or kid or kids or youngster* or youth* or schoolchild* or toddler* or paediatric or pediatric or young people or young person).tw. 3. exp patient reported outcome measures/ 4. (Surveys or Questionnaires).tw. or Patient Health Questionnaire/ 5. exp health status/ 6. (outcome* adj1 measur*).tw. 7. (outcome* adj1 assess*).tw. 8. (health measur* or health assess* or health outcome*).tw. 9. (PROs or PROMs or PROMIS).tw. 10. self-report* measure*.tw. 11. ((patient adj1 report* adj1 outcome adj1 measur*) or (patient adj1 report* adj1 outcome)).tw. 12. ((self adj1 report* adj1 outcome adj1 measur*) or (self adj1 report* adj1 outcome)).tw. 13. ((parent adj1 report* adj1 outcome adj1 measur*) or (parent adj1 report* adj1 outcome)).tw. 14. ((proxy adj1 report* adj1 outcome adj1 measur*) or (proxy adj1 report* adj1 outcome)).tw. 15. ((child adj1 report* adj1 outcome adj1 measur*) or (child adj1 report* adj1 outcome)).tw. 16. ((carer adj1 report* adj1 outcome adj1 measur*) or (carer adj1 report* adj1 outcome)).tw. 17. ((caregiver adj1 report* adj1 outcome adj1 measur*) or (caregiver adj1 report* adj1 outcome)).tw. 18. ((patient or self or child or parent or carer or caregiver or proxy or subjective) adj1 assess* adj1 measur*).tw. 19. ((patient or self or child or parent or carer or caregiver or proxy or subjective) adj1 outcome* adj1 assess*).tw. | |
| 20. ((patient or self or child or parent or carer or caregiver or proxy or subjective) adj1 rate* adj1 health).tw. 21. ((patient or self or child or parent or carer or caregiver or proxy or subjective) adj1 assess* adj1 health).tw. 22. PREMs.tw. 23. Patient report* experience measur*.tw. 24. kidney diseases/ or renal insufficiency/ or renal insufficiency, chronic/ or kidney failure, chronic/ 25. renal replacement therapy/ or hemofiltration/ or exp renal dialysis/ or hemodiafiltration/ or hemodialysis, home/ or peritoneal dialysis/ or exp kidney transplantation/ 26. Renal insufficiency, Chronic/ 27. dialysis.tw. 28. hemodialysis.tw. 29. haemodialysis.tw. 30. hemofiltration.tw. 31. haemofiltration.tw. 32. hemodiafiltration.tw. 33. haemodiafiltration.tw. 34. (kidney disease* or renal disease* or kidney failure or renal failure).tw. 35. (ESRF or ESKF or ESRD or ESKD).tw. 36. (CKF or CKD or CRF or CRD).tw. 37. (CAPD or CCPD or APD).tw. 38. (predialysis or pre-dialysis).tw. 39. Purpura, Schoenlein-henoch/ 40. (congenital kidney disease* or congenital abnormalit* or congenital kidney dyspla* or congenital renal disease* or congenital renal abnormalit* or congenital renal dyspla*).tw. 41. (Henoch Schoenlein or Henoch schonlein).tw. 42. 1 or 2 43. 3 or 4 or 5 or 6 or 7 or 8 or 9 or 10 or 11 or 12 or 13 or 14 or 15 or 16 or 17 or 18 or 19 or 20 or 21 or 22 or 23 44. 24 or 25 or 26 or 27 or 28 or 29 or 30 or 31 or 32 or 33 or 34 or 35 or 36 or 37 or 38 or 39 or 40 or 41 45. 42 and 43 and 44 | |
| **APA Psycinfo via Ovid search strategy (inception to 05/06/2023, updated 30/09/2024 and 01/03/2025)**   1. child*.tw. 2. Adolescen*.tw. 3. teen*.tw. 4. kids*.tw. 5. youngster.tw. 6. youth*.tw. 7. schoolchild*.tw. 8. toddler*.tw. 9. pediatrics/ or exp chronically ill children/ 10. young person*.tw. 11. young people*.tw. 12. exp patient reported outcome measures/ or exp "treatment process and outcome measures"/ or exp patients/ or exp self-report/ or exp treatment outcomes/ 13. exp measurement/ 14. exp questionnaires/ or exp surveys/ 15. exp health screening/ 16. screening/ or exp measurement/ 17. exp health status/ 18. (outcome* adj1 measur*).tw. 19. (outcome* adj1 assess*).tw. 20. (health measur* or health assess* or health outcome*).tw. 21. (PROs or PROMs or PROMIS).tw. 22. self-report* measure*.tw. 23. ((patient adj1 report* adj1 outcome adj1 measur*) or (patient adj1 report* adj1 outcome)).tw. 24. ((self adj1 report* adj1 outcome adj1 measur*) or (self adj1 report* adj1 outcome)).tw. 25. ((parent adj1 report* adj1 outcome adj1 measur*) or (parent adj1 report* adj1 outcome)).tw. 26. ((proxy adj1 report* adj1 outcome adj1 measur*) or (proxy adj1 report* adj1 outcome)).tw. 27. ((child adj1 report* adj1 outcome adj1 measur*) or (child adj1 report* adj1 outcome)).tw. 28. ((carer adj1 report* adj1 outcome adj1 measur*) or (carer adj1 report* adj1 outcome)).tw. 29. ((caregiver adj1 report* adj1 outcome adj1 measur*) or (caregiver adj1 report* adj1 outcome)).tw. 30. ((patient or self or child or parent or carer or caregiver or proxy or subjective) adj1 assess* adj1 measur*).tw. 31. ((patient or self or child or parent or carer or caregiver or proxy or subjective) adj1 assess* adj1 outcome*).tw. 32. ((patient or self or child or parent or carer or caregiver or proxy or subjective) adj1 outcome* adj1 assess*).tw. 33. ((patient or self or child or parent or carer or caregiver or proxy or subjective) adj1 rate* adj1 health).tw. 34. ((patient or self or child or parent or carer or caregiver or proxy or subjective) adj1 assess* adj1 health).tw. 35. PREMs.tw. 36. Patient report* experience measur*.tw. 37. 12 or 13 or 14 or 15 or 16 or 17 or 18 or 19 or 20 or 21 or 22 or 23 or 24 or 25 or 26 or 27 or 28 or 29 or 30 or 31 or 32 or 33 or 34 or 35 or 36 38. (CRF or chronic renal failure or CKF or chronic kidney failure or kidney disease* or renal disease or kidney failure or renal failure or CKD or chronic kidney disease or ESKD or end stage kidney disease or ESKF or end stage kidney failure or ESRF or end stage renal failure or ESRD or end stage renal disease or chronic renal insufficiency or renal insufficiency or kidney insufficiency).tw. 39. (Renal replacement therapy or APD or Automated Peritoneal Dialysis or CAPD, Continuous Ambulatory Peritoneal Dialysis or CCPD or Continuous cyclic peritoneal dialysis or dialysis or h*emofiltration or h*emodiafiltration or h*emodialysis or kidney transplant* or predialysis or pre-dialysis or renal replacement or renal transplant*).tw. 40. ((kidney or renal) adj1 (transplant* or graft* or allograft*)).tw. 41. (congenital kidney disease* or congenital abnormalit* or congenital kidney dyspla* or congenital renal disease* or congenital renal abnormalit* or congenital renal dyspla*).tw. 42. (Nephrotic syndrome or nephritis).tw. 43. (Henoch Schoenlein or Henoch schonlein).tw. 44. 38 or 39 or 40 or 41 or 42 or 43 45. 1 or 2 or 3 or 4 or 5 or 6 or 7 or 8 or 9 or 10 or 11 46. 37 and 44 and 45 | |
| **EMBASE via Ovid search strategy (inception to 01/06/2023, updated 30/09/2024 and 01/03/2025)**   1. exp Child/ 2. exp Adolescent/ 3. (child* or adolescen* or teenager* or kid or kids or youngster* or youth* or schoolchild* or toddler* or paediatric or pediatric or young people or young person).tw. 4. (Surveys or Questionnaires).tw. or Patient reported outcome measures/ or Patient Health Questionnaire/ 5. (outcome* adj1 measure*).tw. 6. (outcome* adj1 assessment*).tw. 7. (health measur* or health assess* or health outcome*).tw. 8. ((patient adj1 report* adj1 outcome adj1 measur*) or (patient adj1 report* adj1 outcome)).tw. 9. ((self adj1 report* adj1 outcome adj1 measur*) or (self adj1 report* adj1 outcome)).tw. 10. ((parent adj1 report* adj1 outcome adj1 measur*) or (parent adj1 report* adj1 outcome)).tw. 11. ((child adj1 report* adj1 outcome adj1 measur*) or (child adj1 report* adj1 outcome)).tw. 12. ((proxy adj1 report* adj1 outcome adj1 measur*) or (proxy adj1 report* adj1 outcome)).tw. 13. ((carer adj1 report* adj1 outcome adj1 measur*) or (carer adj1 report* adj1 outcome)).tw. 14. ((caregiver adj1 report* adj1 outcome adj1 measur*) or (caregiver adj1 report* adj1 outcome)).tw. 15. ((patient or self or child or parent or carer or caregiver or proxy or subjective) adj1 assess* adj1 measur*).tw. 16. ((patient or self or child or parent or carer or caregiver or proxy or subjective) adj1 outcome* adj1 assess*).tw. 17. ((patient or self or child or parent or carer or caregiver or proxy or subjective) adj1 rate* adj1 health*).tw. 18. ((patient or self or child or parent or carer or caregiver or proxy or subjective) adj1 assess* adj1 health*).tw. 19. Patient report* experience measur*.tw. 20. (PROs or PROMs or PROMIS).tw. 21. self-report* measure*.tw. 22. PREMs.tw. 23. patient report* experience measur*.tw. 24. exp renal replacement therapy/ 25. kidney disease/ 26. chronic kidney disease/ 27. kidney failure/ 28. chronic kidney failure/ 29. mild renal impairment/ 30. stage 1 kidney disease/ 31. moderate renal impairment/ 32. severe renal impairment/ 33. end stage renal disease/ 34. renal replacement therapy-dependent renal disease/ 35. kidney transplantation/ 36. (hemodialysis or haemodialysis).tw. 37. (hemofiltration or haemofiltration).tw. 38. (hemodiafiltration or haemodiafiltration).tw. 39. dialysis.tw. 40. (CAPD or CCPD or APD).tw. 41. (kidney disease* or renal disease* or kidney failure or renal failure).tw. 42. (CKF or CKD or CRF or CRD).tw. 43. (ESRF or ESKF or ESRD or ESKD).tw. 44. (predialysis or pre-dialysis).tw. 45. ((kidney or renal) adj (transplant* or graft* or allograft*)).tw. 46. Purpura, Schoenlein-henoch/ 47. (congenital kidney disease* or congenital abnormalit* or congenital kidney dyspla* or congenital renal disease* or congenital renal abnormalit* or congenital renal dyspla*).tw. 48. (Nephrotic syndrome or nephritis).tw. 49. (Henoch Schoenlein or Henoch schonlein).tw. 50. 1 or 2 or 3 51. 4 or 5 or 6 or 7 or 8 or 9 or 10 or 11 or 12 or 13 or 14 or 15 or 16 or 17 or 18 or 19 or 20 or 21 or 22 or 23 52. 24 or 25 or 26 or 27 or 28 or 29 or 30 or 31 or 32 or 33 or 34 or 35 or 36 or 37 or 38 or 39 or 40 or 41 or 42 or 43 or 44 or 45 or 46 or 47 or 48 or 49 53. 50 and 51 and 52 | |
| **CINAHL via EBSCO search strategy (inception to 01/06/2023, updated 30/09/2024 and 01/03/2025)** | |
| S1 | (mh child, preschool ) |
| S2 | (mh child+ ) |
| S3 | AB child* or TI child* |
| S4 | (mh adolescence ) |
| S5 | AB adolescen* or TI adolescen* |
| S6 | AB kids or TI kids |
| S7 | AB( youth or young people or teen* or young adult* or schoolchild or toddler* ) or TI ( youth or young people or teen* or young adult* or schoolchild or toddler*) |
| S8 | AB(p#ediatric) or TI(p#ediatric) |
| S9 | (mh patient-reported outcomes ) or (mh self report ) |
| S10 | AB (patient report* outcomes) or TI (patient report* outcomes) |
| S11 | AB(self-report*) or TI (self-report*) |
| S12 | AB (surveys or questionnaires) or TI(surveys or questionnaires) |
| S13 | mh health status |
| S14 | AB (outcome N1 measure*) or TI(outcome N1 measure*) |
| S15 | AB (outcome N1 assess*) or TI(outcome N1 assess*) |
| S16 | AB(health measur* or health assess* or health outcome*) or TI(health measur* or health assess* or health outcome*) |
| S17 | AB( PROs or PROMs or PROMIS) or TI( PROs or PROMs or PROMIS) |
| S18 | AB(self-report* measure*) or TI(self-report* measure*) |
| S19 | AB(patient N1 report* N1 outcome* N1 measur*) or TI(patient N1 report* N1 outcome* N1 measur*) or AB(patient N1 report* N1 outcome*) or TI(patient N1 report* N1 outcome*) |
| S20 | AB(self N1 report* N1 outcome* N1 measur*) or TI(self N1 report* N1 outcome* N1 measur*) or AB(self N1 report* N1 outcome*) or TI(self N1 report* N1 outcome*) |
| S21 | AB(parent N1 report* N1 outcome* N1 measur*) or TI(parent N1 report* N1 outcome* N1 measur*) or AB(parent N1 report* N1 outcome*) or TI(parent N1 report* N1 outcome*) |
| S22 | AB(proxy N1 report* N1 outcome* N1 measur*) or TI(proxy N1 report* N1 outcome* N1 measur*) or AB(proxy N1 report* N1 outcome*) or TI(proxy N1 report* N1 outcome*) |
| S23 | AB(child N1 report* N1 outcome* N1 measur*) or TI(child N1 report* N1 outcome* N1 measur*) or AB(child N1 report* N1 outcome*) or TI(child N1 report* N1 outcome*) |
| S24 | AB(carer N1 report* N1 outcome* N1 measur*) or TI(carer N1 report* N1 outcome* N1 measur*) or AB(carer N1 report* N1 outcome*) or TI(carer N1 report* N1 outcome*) |
| S25 | AB(caregiver N1 report* N1 outcome* N1 measur*) or TI(caregiver N1 report* N1 outcome* N1 measur*) or AB(caregiver N1 report* N1 outcome*) or TI(caregiver N1 report* N1 outcome*) |
| S26 | AB((patient or self or child or parent or carer or caregiver or proxy or subjective) N1 outcome* N1 assess*)) or TI((patient or self or child or parent or carer or caregiver or proxy or subjective) N1 outcome* N1 assess*)) |
| S27 | AB((patient or self or child or parent or carer or caregiver or proxy or subjective) N1 assess* N1 measur*)) or TI((patient or self or child or parent or carer or caregiver or proxy or subjective) N1 assess* N1 measur*)) |
| S28 | AB((patient or self or child or parent or carer or caregiver or proxy or subjective) N1 rate* N1 health*)) or TI((patient or self or child or parent or carer or caregiver or proxy or subjective) N1 rate* N1 health*)) |
| S29 | AB((patient or self or child or parent or carer or caregiver or proxy or subjective) N1 assess* N1 health*)) or TI((patient or self or child or parent or carer or caregiver or proxy or subjective) N1 assess* N1 health*)) |
| S30 | MH patient-reported experience measures or prem |
| S31 | AB(patient-reported experience measures or prem) or TI(patient-reported experience measures or prem) |
| S32 | (MH "Renal Insufficiency, Chronic+") OR (MH "Kidney Failure, Chronic") |
| S33 | CKD or CRD or CKF or CRF |
| S34 | ESKD or ESRD or ESKF or ESRF |
| S35 | AB(chronic kidney or chronic renal or chronic nephropath) or TI(chronic kidney or chronic renal or chronic nephropath) |
| S36 | AB(kidney failure or renal failure) or TI(kidney failure or renal failure) |
| S37 | AB(kidney insufficienc* or renal insufficienc*) or TI(kidney insufficienc* or renal insufficienc*) |
| S38 | AB( endstage kidney or endstage renal) or TI( endstage kidney or endstage renal ) |
| S39 | AB( end-stage kidney or end-stage renal ) or TI( end-stage kidney or end-stage renal ) |
| S40 | (MH "Renal Replacement Therapy+") |
| S41 | AB(dialysis or predialysis ) or TI(dialysis or predialysis ) |
| S42 | AB( h?emodialysis or h?emofiltration or h?emodiafiltration) or TI( h?emodialysis or h?emofiltration or h?emodiafiltration) |
| S43 | S33 AB(kidney transplant* or renal transplant*) or TI(kidney transplant* or renal transplant*) |
| S44 | AB(kidney graft* or renal graft*) or TI(kidney graft* or renal graft*) |
| S45 | AB(kidney replacement* or renal replacement*) or TI(kidney replacement* or renal replacement*) |
| S46 | CAPD or CCPD or APD |
| S47 | AB(pre-dialysis or predialysis) or TI(pre-dialysis or predialysis) |
| S48 | MH(Purpura, Schoenlein-henoch) |
| S49 | AB(Henoch Schoenlein or Henoch schonlein) or TI(Henoch Schoenlein or Henoch schonlein) |
| S50 | AB( congenital kidney disease* or congenital abnormalit* or congenital kidney dyspla* or congenital renal disease* or congenital renal abnormalit* or congeital renal dyspla*) or TI(congenital kidney disease* or congenital abnormalit* or congenital kidney dyspla* or congenital renal disease* or congenital renal abnormalit* or congeital renal dyspla*) |
| S51 | S1 OR S2 OR S3 OR S4 OR S5 OR S6 OR S7 OR S8 |
| S52 | S9 OR S10 OR S11 OR S12 OR S13 OR S14 OR S15 OR S16 OR S17 OR S18 OR S19 OR S20 OR S21 OR S22 OR S23 OR S24 OR S25 OR S26 OR S27 OR S28 OR S29 OR S30 OR S31 |
| S53 | S32 OR S33 OR S34 OR S35 OR S36 OR S37 OR S38 OR S39 OR S40 OR S41 OR S42 OR S43 OR S44 OR S45 OR S46 OR S47 OR S48 OR S49 OR S50 |
| S54 | S51 AND S52 AND S53 |

**Table S3. Characteristics of included studies (n=175)**

| Study | Country | Study type | Study objective | Total CKD patients and caregivers | | CKD  stage | Child  age  range | Child gender (Male %) | No.  of PROMs/ PREMs used | PROMs/PREMs used | Outcome domains covered |
| --- | --- | --- | --- | --- | --- | --- | --- | --- | --- | --- | --- |
|  |  |  |  | P | C |  |  |  |  |  |  |
| Abbasi  2022 [1] | Iran | Case-control | To gain a better understanding of the QoL of children with NS | 60 | NR | Early CKD  n=60 | 6 - 18 | 58 | 1 | PedsQL 4.0 | QoL |
| Abrao  2021[2] | Brazil | Cross-sectional | To describe and analyze the QoL and behavioral problems of children and adolescents with CKD stages 3-5, as well as the quality of life and mental health of their parent/caregivers and their association | 80 | 80 | CKD n=30 PD/HD n=20 Tx n=30 | 8 - 18 | 55 | 4 | PedsQL 4.0   PedsQL 3.0 – ESRD   CBCL   YSR | Behavior/mental health QoL |
| Abreu  2014[3] | Brazil | Qualitative | To identify the impactful attributes on children and adolescents’ QoL with CRF on dialysis treatment as an initial step to build a QoL measurement instrument for this population | 21 | 21 | HD n=21 | 8 - 18 | NR | 1 | Instrument developed by DISABKIDS which has 12 open questions | QoL |
| Agrawal 2017[4] | India | Cross-sectional | To evaluate the QoL in children with NS and delineate the QoL domains in various clinical subtypes of NS | 50 | NR | NS | 2.2 - 15 | 60 | 1 | PedsQL 4.0 | QoL |
| Ahmed  2022[5] | Canada | Cross-sectional | To explore the strength of association with self- reported levels of physical activity and with baseline and contemporaneous transplant-related clinical factors | 46 | N/A | Tx n=46 | 12 - 18 | 56.5 | 2 | PAQ-C   PAQ-A | Physical activity |
| Al-Uzri  2013[6] | USA | Cross-sectional | To compare the QoL of children with CKD and short stature (SS) to children with CKD and normal height (NH), to evaluate the impact of catch-up growth and growth  hormone use on QoL, and to describe the concordance of perceptions of QoL between children with SS and NH and their parents. | 483 | 483 | Early CKD  n=483 | 8 - 17 | SS: 32  NH: 63 | 1 | PedsQL 4.0 | QoL |
| Albatati 2024[7] | Saudi Arabia | Cross-sectional | To explore the impact of social determinants of health on the QoL of children with CKD in Saudi Arabia | 50 | 50 | PD n=10 HD n=9 CKD n=31 | 2 - 18 | 56 | 1 | PedsQL 3.0 (Arabic version) | QoL |
| Alhamed  2022[8] | USA  and Canada | Cross-sectional (secondary analysis) | To examine the relationship between executive functions, socio-emotional functioning and QoL in children and adolescents with mild to moderate CKD | 199 | N/A | Early CKD n=199 | 5 - 17 | 59 | 3 | PedsQL 4.0  BRIEF (parent report)  BASC-2 (parent report) | Behavior/mental health QoL |
| Alhusaini  2019[9] | Saudi Arabia | Cross-sectional | To record pediatric ESRD patients’ QoL in relation to PD and HD | 23 | 23 | PD n=14,  HD n=9 | 2 - 18 | PD: 71.4 HD: 44.4 | 1 | PedsQL 3.0 ESRD module | QoL |
| Almardini  2019[10] | Ireland and Jordan | Cross-sectional | To use a multiple methods approach to assess adherence to mycophenolate in children with kidney transplant, to identify patient/parental factors that influenced adherence and to link adherence behavior to clinical outcomes | 33 | 33 | Tx n=29 | 8 - 18 | 61 | 3 | Beliefs about medicines questionnaires  Medication adherence report scale (MARS) - specific adherence over the last month (parent and child version)  PedsQL 3.0 Transplant module | Behavior/mental health QoL  Treatment adherence |
| AlQaisy 2019[11] | Iraq | Case-control | To assess the QoL in a sample of Iraqi children with NS in relation to children with other chronic disease | 50 | NR | NS | 2 - 12 | 66 | 1 | PedsQL 4.0 | QoL |
| Aman  2022[12] | Canada | Cohort | To understand the family functioning and parental and child quality of life during the pandemic compared to pre-pandemic assessment | 107 | 107 | Early CKD, NS | 0.5 - 18 | 71 | 1 | PedsQL 4.0 | QoL |
| Amin  2015[13] | Canada | Cross-sectional | To ascertain the prevalence and type of sleep-disordered breathing in paediatric patients with severe CKD based on the results of polysomnograms | 19 | NR | Early CKD n=12 Dx n=7 | 8 - 17 | 42 | 3 | Epworth Sleepiness Scale (ESS)  PedsQL  PSQ | QoL Sleep |
| Amr  2009[14] | Egypt | Mixed method | To describe the psychological adjustment in children with CKD | 38 | NR | HD n=19 Early CKD n=19 | 9 - 15 | Dx: 58 Pre-Dx: 68 | 1 | CBCL | Behavior/mental health |
| Anthony  2010[15] | Canada | Cross-sectional | To examine multidimensional QoL outcomes after kidney transplant using VAQOL and General Health, the PedsQL 4.0, PedsQL ESRD Module, and Impact on Family Module. | 21 | 21 | Tx n=21 | 2 - 18 | 71 | 3 | VAQOL (visual analogue quality of life)  PedsQL 4.0  PedsQL 3.0 ESRD module | QoL |
| Aparicio Lopez  2010[16] | Spain | Cross-sectional | To assess QoL in children with CKD and compare it to healthy children. | 71 | NR | Early CKD n=22 Tx n=33 PD n=16 HD n=5 | - | 55 | 1 | MOS-SF-20 (20-item Medical Outcomes Study) | QoL |
| Aparicio Lopez  2010[17] | Spain | Cross-sectional | To design a specific questionnaire for kidney disease in children based on the QoL test for adults with kidney disease and on the test of QoL for children with epilepsy adapting them for children with kidney disease | 71 | NR | - | - | - | 1 | TECAVNER (Test of Quality of Life in Children with kidney disease) | QoL |
| Aparicio Lopez  2013[18] | Spain | Cross-sectional | To analyze the impact of laboratory variables and various clinical situations on HRHR of pediatric CKD patients | 71 | NR | HD n=5 PD n=11 Tx n=33 Early CKD n=22 | NR | 55 | 1 | TECAVNER (Test of Quality of Life in Children with kidney disease) | QoL |
| Ayestaran  2016[19] | USA | Cross-sectional | To investigate the relationship between self-reported appetite and clinical outcomes in children with CKD | 879 | NR | NR | NR | 62 | 2 | Appetite and diet assessment tool (ADAT)  PedsQL | Diet/nutrition QoL |
| Azukaitis 2023[20] | Lithuania | Cross-sectional | To explore the feasibility and potential relevance of integrated electronic collection of PROMs and PREMs in children with special healthcare needs | 98 | 192 | NR | 0 - 18 | 57.80 | 2 | Author-initiated PREM questionnaire  PedsQL 4.0 (Lithuanian version) | Treatment experience  QoL |
| Baek  2017[21] | Korea | Cross-sectional | To evaluate the QoL of Asian children with pre-dialysis CKD and to reveal the factors influencing the QoL of children with CKD | 376 | NR | Early CKD n=376 | 0 - 17 | Parent proxy-reports: 68.1 Child self-reports: 69.4 | 2 | PedsQL 4.0 Generic Core Scale Module  K-CBCL (Korean child behavior checklist) | Behavior/mental health  QoL |
| Baek  2018[22] | Korea | Cross-sectional | To investigate the impact of pediatric ESRD on parents, based on the PedsQL family impact module, and the relationship to the QoL of pediatric ESRD patients measured by PedsQL 3.0 module | 79 | NR | HD n=8 PD n=39 Tx n=32 | 8 - 18 | 39 | 1 | PedsQL 3.0 ESRD module | QoL |
| Bakar  2023[23] | Malaysia | Cross-sectional | To validate the Bahasa Melayu (Malay language) version of PedsQL 3.0 ESRD module and determine the QoL scores amongst children with CKD in Malaysia | 69 | 69 | Early CKD n=18 HD n=22 PD n=29 | 8 - 18 | 45 | 2 | PedsQL 3.0 ESRD module (Malay version)  PedsQL 4.0 | QoL |
| Bamford  2016[24] | United Kingdom | Cross-sectional | To investigate whether psychology annual reviews have facilitated identification of patients and families experiencing psychosocial difficulties | 20 | NR | Tx n=20 | 3 - 17 | 65 | 3 | PedsQL Transplant Module 3.0  Pediatric Index of Emotional Distress (PI-ED)  Hospital Anxiety and Depression Scale (HADS) | Behavior/mental health QoL |
| Bauer  2021[25] | USA | Cross-sectional | To utilize the novel Pediatric renal- caregiver burden survey tool to describe caregiver burden in a pediatric dialysis population at three pediatric dialysis centers in the USA | 31 | 31 | HD n=21 PD n=10 | NR | 61.30 | 1 | Pediatric Renal Caregiver Burden Scale (PR-CBS) | Treatment experience |
| Berney-Martinet  2009[26] | Canada | Mixed method | To describe the psychological profile of renal transplant adolescents compared to healthy peers and to adolescents with CKD | 60 | NR | Tx n=40 CKD n=20 | 12 - 18 | Tx: 65 CKD: 55 | 2 | CBCL  YSR | Behavior/mental health |
| Brunelli  2012[27] | Brazil | Cross-sectional | To analyze the QoL in a pediatric CKD population and compare it with healthy controls | 33 | NR | HD n=22 PD n=11 | 4 - 12 | 58 | 1 | Autoquestionnaire Qualite´ de Vie Enfant Image´(AUQEI) | QoL |
| Buyan  2010[28] | Turkey | Cross-sectional | To compare the QoL scores of Turkish children who are dialysis patients, renal transplant recipients, and age-matched healthy controls and to compare child-self and parent-proxy scores | 211 | NR | Dx n=72 Tx n=139 | 4 - 18 | 49 | 1 | The Kinder Lebensqualität Fragebogen (KINDL) questionnaire for children (Turkish version) | QoL |
| Buyukkaragoz  2016[29] | Turkey | Cross-sectional | To evaluate the QoL of pediatric renal transplant recipients and their parents and investigate the effects of clinical, socioeconomic, and psychological factors as well as continuous counselling | 12 | 11 | Tx n=12 | 8 - 18 | 66.7 | 3 | Beck Depression Inventory (BDI)  Rosenberg Self-Esteem Scale (RSES)  The Kinder Lebensqualität Fragebogen (KINDL) questionnaire for children (Turkish version) | Behavior/mental health QoL |
| Carlson  2020[30] | USA | Cross-sectional | To evaluate impact of anemia on QoL over time in a large pediatric cohort with mild-to-moderate CKD | 773 | N/A | Early CKD n=773 | NR | 61 | 1 | PedsQL 4.0 | QoL |
| Carlson 2023[31] | USA and Canada | Cohort | To determine how QoL changes over time in childhood CKD | 692 | NR | CKD n=692 | 1-16 | 62 | 1 | PedsQL Generic Core Scales | QoL |
| Cavusoglu  2023[32] | Turkey | Cross-sectional | To compare the effects of Nintendo Wii-based exergaming and home-based fun video exercises on functional capacity, muscle strength, physical activity, depression, fatigue, and QoL in pediatric patients with CKD | 16 | NR | Early CKD n=16 | 5 - 18 | Group 1: 62.5  Group 2: 75 | 2 | PedsQL 4.0  Child depression inventory (CDI) | Behavior/mental health  Fatigue QoL |
| Chan  2022[33] | Hong Kong | Cohort | To provide prospective data on important clinical outcome measures and evaluate patient QoL among children receiving APD with remote patient monitoring | 17 | N/A | PD n=17 | 0 - 18 | 29 | 1 | PedsQL 3.0 ESRD module | QoL |
| Chan  2024[34] | USA | Cohort | To evaluate the effectiveness of a structured education curriculum for adolescents and young adults with CKD | 42 | NR | Early CKD | 14 - 17 | 62 | 1 | Transition Readiness Assessment Questionnaire (TRAQ) | Transition/coping |
| Cheung 2015[35] | USA | Cross-sectional | To determine the prevalence of RLS in children with nephrotic syndrome | 50 | NR | NR | 3 - 18 | 54 | 3 | Pediatric Emory RLS questionnaire  Pediatric Daytime Sleepiness Scale  Pediatric Sleep Questionnaire | Sleep |
| Chien-Ning  2018[36] | Taiwan | Cross-sectional | (i) To assess QoL in pediatric patients with mild-to-moderate CKD states as measured by the visual analogue scale and descriptive system of the EQ-5D-Y (ii) To investigate the validity and reliability of Taiwanese version of EQ-5D-Y. | 68 | NR | Early CKD n=68 | 7 - 18 | 64.70 | 1 | EQ-5D-Y | QoL |
| Clark  2016[37] | USA | Cross-sectional | To examine self-reported physical activity and screen time in adolescents with CKD in comparison with healthy peers and to characterize changes in physical activity and screen time with progression of CKD | 224 | NR | Early CKD n=224 | 12 - 18 | 60 | 1 | Physical activity and screen time questionnaire (modelled after the United States Centers for Disease Control National Health and Nutrition Examination Survey (NHANES) questionnaire) | Physical activity |
| Clave  2019[38] | France | Cohort | To describe the QoL of adolescents initiating HD, to determine the factors associated with QoL, and to assess coping strategies and their impact on QoL | 32 | NR | HD n=32 | 11 - 17 | 62.5 | 2 | Vécu et Santé Perçue de l’Adolescent” (VSP-A)  Kidcope | Behavior/mental health QoL |
| Coleman  2002[39] | UK | Cohort | To compare a newly formulated renal-specific micronutrient supplement with Ketovite tablets in children with CKD or on chronic PD and HD | 14 | NR | Early CKD n=11 CPD n=2 HD n=2 | 1.1 -16 | NR | 1 | Questionnaires on palatability, acceptability, and ease of administration of medications | Treatment adherence |
| Conde Garcia  2010[40] | Spain | Cross-sectional | To identify the difficulties presented by families and patients for correct compliance with treatment in pediatric recipients of renal transplants | 41 | NR | Tx n=41 | 5 - 18 | 68 | 1 | Questionnaire designed with closed questions on demographic aspects, knowledge and attitudes to the treatment received, self-sufficiency or dependence in administration, compliance and tiredness of the patient and carer. | Treatment adherence |
| Craven  2023[41] | USA | Cross-sectional | To examine patient- and caregiver-reported factors associated with school absenteeism to help identify modifiable risk factors to optimize school attendance | 48 | 48 | Early CKD n=48 | 7 - 18 | 69 | 1 | Questionnaire created by study team to examine reasons for absenteeism among children with chronic disease | Schooling |
| Davis  2012[42] | USA and Canada | Cross-sectional | To define the prevalence of a variety of sleep disturbance symptoms in a large pediatric CKD population and to evaluate whether these symptoms were associated with lower QoL in this unique cohort | 159 | NR | HD/PD n=30 Tx n=61 Early CKD n=68 | 5 - 18 | 50.90 | 4 | Pediatric modification of the Epworth Sleepiness Scale (ESS)  Pediatric Sleep Questionnaire (PSQ)  RLS symptom questionnaire derived from standard criteria for the diagnosis of RLS in children and adolescents  PedsQL 4.0 | QoL Sleep |
| De Bruyne  2022[43] | Belgium | Cross-sectional | To translate the original version of the CKD-specific PedsQL™ 3.0 ESRD Module into a Dutch version and to evaluate its validity and reliability | 58 | 31 | HD n=2 PD n=2 Early CKD n=54 | 8 - 18 | 66 | 2 | PedsQL 4.0  PedsQL™ 3.0 End Stage Renal Disease Module | QoL |
| De Pádua Paz  2021[44] | Brazil | Cross-sectional | To identify main factors associated with child and adolescent access to CKD treatment in Brazil | 335 | 335 | Early CKD n=335 | 0 - <19 | 60 | 1 | Medical Outcomes Study Social Support Survey (MOS) | Access to treatment |
| Demir  2023[45] | Turkey | Cross-sectional | To evaluate the effect of the presence of a psychiatric disorder on the QoL in children and adolescents after kidney transplantation | 43 | NR | Tx n=43 | 6 - 18 | 55.80 | 2 | PedsQL  Strengths and difficulties questionnaire (SDQ) | Behavior/mental health QoL |
| DeWalt  2015[46] | USA | Cross-sectional | To conduct a comparative analysis of eight pediatric self-report scales for ages 8-17 years from the NIH PROMIS® in six pediatric chronic health conditions, using indicators of disease severity | 384 | NR | Early CKD n=384 | 8 - 17 | 57.80 | 6 | Patient Reported Outcomes Measurement Information System (PROMIS) pediatric measures of mobility, upper extremity functioning, depressive symptoms, anxiety, anger, peer relationships, pain interference, and fatigue | Behavior/mental health Fatigue QoL Mobility Social well-being Symptoms |
| Diaz-Gonzalez de Ferris  2021[47] | USA | Cross-sectional | To investigate the impact of medications on QoL in CKD patients, regardless of CKD severity, diagnosis, or duration of disease | 734 | NR | Early CKD n=734 | NR | 61 | 1 | PedsQL 4.0 | QoL |
| Didsbury  2019[48] | Australia and New Zealand | Cohort | To determine the association of socioeconomic disadvantage and parent-rated health in children with CKD | 377 | 377 | Early CKD n=199 Dialysis n=43 Tx n=135 | 6 - 18 | 62 | 1 | Self-rated health (SRH) | Overall health/health status |
| Dobbels  2010[49] | Belgium | Cross-sectional | To evaluate depression, QoL, treatment adherence and presence of side effects from the perspective of the patient and their parents, and to compare scores with norm data. | 23 | 23 | Tx n=23 | 10 - 18 | 69.6 | 5 | KIDSCREEN-27   Beck Depression Inventory (BDI)  MTSOSD-59R (adapted from adult Tx version)  BAASIS (self-report interview) - treatment adherence to immunosuppressive regimen  Developed a semi-structured interview for adherence to other aspects of the therapeutic regimen | Behavior/mental health QoL Symptoms Treatment adherence |
| Dodson  2009[50] | USA and Canada | Cross-sectional | To determine the prevalence and impact of incontinence on QoL in children with chronic kidney disease | 329 | NR | Early CKD n=329 | 5 - 16 | 61.40 | 1 | PedsQL | QoL |
| Dodson  2007[51] | USA | Cross-sectional | To investigate the QoL of adolescents with CKD due to an underlying congenital urological diagnosis and compare this to adolescents with medical kidney disease and to population-based norms | 113 | NR | CAKUT group  Early CKD n=35 Tx n=60 Dx n=5  Other diagnoses group  Early CKD n=33 Tx n=40 Dx n=27 | 10 - 18 | 63 | 1 | Child Health and Illness Profile-Adolescent Edition (CHIP-AE) | QoL |
| Dodson  2008[52] | USA | Cohort | To assess QoL in adolescents with CKD due to urological anomalies using a validated parent proxy instrument | 92 | 92 | CAKUT group Tx n=22 Early CKD n=13  Other diagnoses group Tx n=31 Early CKD n=26 | 11 - 18 | CAKUT (77.1)  Other diagnoses (57.9) | 1 | Child Health Questionnaire-Parent Form 50 (CHQ-P50) | QoL |
| Dotis  2016[53] | Greece | Cross-sectional | To record the QoL of children in Greece with CKD on PD or who have had a renal transplant and compare this with healthy children | 55 | 54 | Tx n=16 PD n=14 Early CKD n=25 | 8 - 18 | 49.09 | 1 | KIDSCREEN (Greek translation) | QoL |
| Duverger 2013[54] | France | Mixed method | (i) To verify certain psychopathological hypotheses concerning the existence of anxiety and depressive disorders in young kidney transplant recipients (ii) To analyze the QoL of these young kidney transplant recipients | 40 | NR | Tx n=40 | 5 - 15 | 62.50 | 6 | Anxiety scales (STAI)   Depression scales (CDI and CDRS-R)  Overall functioning (CGAS)  Evaluation of quality of life (AUQUEI/OK ADO) | Behavior/mental health QoL |
| Eid  2020[55] | Egypt | Cross-sectional | To evaluate QoL in Egyptian children with idiopathic NS in comparison to healthy children and children with chronic non-renal illnesses | 100 | NR | NR | 8 - 18 | 58 | 2 | GCQ  PedsQL 4.0 | QoL |
| Eijsermans 2004[56] | Netherlands | Cross-Sectional | To investigate QoL and exercise tolerance in children with ESRD undergoing chronic intermittent HD | 10 | N/A | HD n=10 | 7.3 -16.9 | 80 | 1 | TNO-AZL Child Quality of Life (TACQOL) | QoL |
| El Shafei 2018[57] | Egypt | Cross-Sectional | To assess the QoL among children with ESRD, either undergoing HD or had renal transplantation therapy and comparing it with healthy controls | 55 | - | NR | 5 - 18 | 50.90 | 2 | PedsQL 4.0 (Arabic version)  PedsQL 3.0 ESRD module | QoL |
| El-baroudy 2020[58] | Egypt | Cross-Sectional | To characterize the types of sleep disorders in children on regular HD and to detect the predictors of sleep efficiency in those children | 40 | N/A | HD n=40 | 6-15 | 35 | 1 | Epworth Sleepiness Scale (ESS) | Sleep |
| El-Gamasy 2017[59] | Egypt | Cross-Sectional | To clarify biodemographic characteristics, common complaints, and physical, and psychosocial status of children with ESRD under regular maintenance HD | 30 | NR | HD n=30 | 6 - 16 | 43.30 | 1 | CBCL | Behavior/mental health |
| El-Refaey 2013[60] | Egypt | Cross-Sectional | To assess the sleep quality in children on HD | 25 | - | HD n=25 | 8 - 18 | 52 | 1 | Pediatric Sleep questionnaire (PSQ) | Sleep |
| Elabbassy 2015[61] | Egypt | Other | To evaluate the effect of practical guide on burden's coping strategies among caregiver of children undergoing HD | 50 | 50 | HD N=50 | 0 - 18 | 46 | 1 | Pediatric Renal Caregiver Burden Scale (PR-CBS) | Treatment experience |
| Esmaeeli 2014[62] | Iran | Cross-Sectional | To examine the prevalence of depression in hospitalized children with chronic and acute conditions in Dr Sheikh Pediatrics Hospital in Mashhad | 30 | NR | NR | 8 - 16 | NR | 1 | Children Depression Inventory questionnaire (CDI) | Behavior/mental health |
| Fadrowski 2006[63] | USA | Cohort | To investigate which clinical parameters influence functional status or health-related QoL in these young people with CKD | 78 | - | Tx n=40 Dx n=11 | 11 - 18 | 64 | 1 | CHQ-PF50 | QoL |
| Falger  2008[64] | Switzerland | Cross-Sectional | To conduct a comprehensive evaluation of both QOL and PA in children and adolescents after RTPL by use of standardized questionnaires for patients and parents | 37 | NR | Tx n=37 | 5 - 17 | 54 | 5 | Brief symptom inventory  Child Behavior Checklist (CBCL)  Family relationship index (FRI)  Childhood post-traumatic stress disorder reaction index (CPTSD-RI)  The Netherlands Organization for Applied Scientific Research Academical Medical Centre (TNO-AZL) Child Quality of Life Questionnaire (TACQOL) | Behavior/mental health  Social well-being QoL |
| Fayer Rotella  2020[65] | Brazil | Mixed -method | To investigate the emotional repercussions and QoL associated with ESKD in children and adolescents undergoing HD or a kidney transplant | 48 | 48 | HD n=24 Tx n=24 | 5 - 18 | HD pts: 33.3  Tx pts: 58.3 | 1 | PedsQL 4.0 | QoL |
| Fenton 2015[66] | USA | Mixed -method | To conceptualize how disease-related risk factors and psychosocial resilience factors influence health care transition readiness when controlling for age and disease severity | 41 | NR | Early CKD n=41 | 13 - 18 | 68 | 5 | Family relationship index (FRI)  KIDCOPE scale  PedsQL 4.0   The Medication Adherence Measure  UNC TRxANSITION Scale | QoL Social well-being Transition/coping Treatment adherence |
| Forrest 2020[67] | USA | Qualitative | To evaluate the content validity of the PROMIS Pediatric Fatigue and sleep measures for children with CKD and children with Crohn’s disease | 26 | NR | NR | 8 - 17 | 58 | 1 | Semi-structured interviews evaluating Pediatric PROMIS fatigue and sleep measures | Sleep |
| Francis 2019[68] | Australia and New Zealand | Cross-Sectional | To compare QoL among children and adolescents with different stages of CKD and determine factors associated with changes in QoL | 375 | NR | Early CKD n=197 Dx n=43 Tx n=135 | 6 - 18 | 62 | 1 | Health Utilities Index (HUI) | QoL |
| Gerson 2004[69] | USA | Cohort | To examine the association between anemia and QoL in adolescents with CKD | 105 | NR | Early CKD n=37 Dx n=13 Tx n=50 | 11 - 18 | 66 | 1 | Child health questionnaire – parent form 50 (CHQP50) | QoL |
| Gerson 2005[70] | USA | Cross-Sectional | To assess scores on the CHIP-AE in adolescents with CKD compared with two control groups and to compare health of patients who had CKD, were on Dx, and were post-Tx | 113 | NR | Early CKD n=39 Dx n=21 Tx n = 53 | 10 - 18 | 64 | 1 | CHIP-AE | QoL |
| Gerson 2010[71] | USA and Canada | Cross-Sectional | To compare the QoL of children with CKD to healthy children, to evaluate the association between CKD severity and QOL, and to identity demographic, socioeconomic and health-status variables associated with impairment in QoL in children with mild to moderate CKD | 402 | NR | Early CKD n=402 | 2 - 16 | 60 | 1 | PedsQL 4.0 | QoL |
| Gipson  2013[72] | USA | Cross-sectional | To evaluate the ability of children and adolescents with NS to complete the PROMIS assessment and to initiate validity assessments of the short forms and full item banks in pediatric NS | 151 | 150 | NS | 8 - 17 | 52.20 | 1 | PROMIS Pediatric-36 Profile | Behavior/mental health  Mobility Social well-being Symptoms |
| Goldstein 2006[73] | USA | Cross-Sectional | To utilize pilot data to create a practical disease-specific assessment instrument that can be used longitudinally, and which can be integrated with a brief generic QoL instrument. | 96 | 95 | NR | 2 - 18 | NR | 1 | PedsQL 4.0 | QoL |
| Goldstein 2008[74] | USA | Cross-Sectional | To investigate the feasibility, reliability, and validity of the PedsQL 3.0 ESRD Module and the PedsQL 4.0 Generic Core Scales at 4 pediatric ESRD centers | 193 | 190 | HD n=30 PD n=45 Tx n=127 Unavailable data n=6 | 2 - 18 | 53.80 | 2 | PedsQL 3.0 ESRD Module  PedsQL 4.0 | QoL |
| Goldstein 2009[75] | USA | Cross-Sectional | To investigate the differences in reported ESRD-specific QoL across renal transplant and maintenance dialysis modalities utilizing the PedsQL ESRD Module | 186 | NR | HD n=30 PD n=45 Tx n=127 | 3 - 18 | HD pts: 56.7  PD pts: 64.4  Tx pts: 50.4 | 2 | PedsQL 3.0 ESRD Module  PedsQL Family Information Form | QoL |
| Gomes 2017[76] | Portugal | Cross-Sectional | To describe the prevalence of sleep disturbances in a cohort of children on APD evaluated by polysomnography complemented by the results of a validated questionnaire | 8 | NR | PD n=8 | 1 - 18 | 62.50 | 1 | Author-initiated sleep questionnaire | Sleep |
| Guha  2023[77] | Australia | RCT | To determine the effectiveness of a patient navigator program in children with CKD | 162 | 162 | Early CKD n=109 HD n=3 PD n=8 Waiting list for Tx n=9 Tx n=42 | 0 - 16 | 64.80 | 2 | Health Utilities Index Mark 3 (HUI3)  Self-rated health (SRH) | QoL  Overall health/ health status |
| Guha  2023[78] | Australia and New Zealand | Cross-Sectional | To assess the trajectories of QoL in children with CKD over time | 377 | 377 | Early CKD n=199 Dx n=43 Tx n=135 | 6 - 18 | 62 | 1 | Health Utilities Index Mark 3 (HUI3) | QoL |
| Guilfoyle 2011[79] | USA | Cross-Sectional | To identify sociodemographic and medical correlates of both caregiver and patient-reported general family functioning and determining the unique contributions of these to perceived post-transplant adherence barriers | 45 | 45 | Tx n=45 | 7 - 18 | 60.5 | 3 | Adolescent Medication Barriers Scale (AMBS)  FACES–IV  Perceived Collective Family Efficacy Scale (PCFES) | Social well-being Treatment adherence |
| Guzman-Carrillo 2022[80] | Mexico | Cohort | To evaluate the efficacy of a cognitive behavioral intervention to improve adherence to treatment and QoL and promote psychological resources in pediatric CKD patients undergoing HD and at the same time reduce the burden on their primary caregivers | 5 | 5 | HD n=5 | 12 - 17 | 20 | 3 | Family Relationship Scale (FRI)  Individual Psychological Resources Scale  PedsQL 4.0 | Behavior/mental health Diet/nutrition QoL Social well-being Treatment adherence |
| Haavisto 2013[81] | Finland | Cross-sectional | To compare QoL and PSA in school-aged heart-, kidney-, and liver Tx patients treated at the same center without exclusion of patients with major comorbidity. | 44 | 44 | Tx n=44 | 6.3 - 16.7 | 57 | 2 | 17D, 16D and 15D  Child behavior checklist (CBCL)  Youth self-report (YSR) | Behavior/mental health QoL |
| Hajj  2024[82] | France | Cohort | To evaluate the impact of PD on children and families’ QoL | 12 | 12 | PD n=12 | 1 - 17 | NR | 1 | PedsQL3.0 ESRD (French translation) | QoL |
| Hamedi-Kalajahi 2022[83] | Iran | RCT | To investigate the effect of L-carnitine supplementation on inflammatory markers and FBS, as the risk factors of CKD among HD children. | 24 | - | HD n=24 | 6 - 18 | L-carnitine: 58.3  Placebo: 50 | 2 | Physical activity questionnaire - child (PAQ-C)  PedsQL 4.0 (Iranian version) | QoL Physical activity/mobility |
| Harmer 2019[84] | UK | Cross-Sectional | To report the QoL scores as assessed by the validated PedsQL questionnaire and to explore the relationship of QoL scores to markers of nutritional status | 46 | - | Early CKD n=46 | 3 - 18 | 60.87 | 1 | PedsQL | QoL |
| Heath  2011[85] | UK | Cross-Sectional | To investigate the self-reported QoL of children and young people with CKD | 124 | - | HD n=8 PD n=16 Tx n=68 Early CKD n=32 | 6.2 - 15 | 52.40 | 1 | Generic Children’s Quality of Life Measure (GCQ) | QoL |
| Heath  2017[86] | UK | Cross-Sectional | To evaluate a generic and renal-specific self-report QoL scale, assess children’s attitudes towards living with CKD and propose an appropriate tool for future individual clinical use or departmental audit | 28 | - | HD n=9 PD n=0 Tx n=44 Early CKD n=18 | 6 - 12 | NR | 3 | Child attitude towards illness (CATIS)  Generic Children’s Quality of Life Measure (GCQ)  PedsQL 3.0 ESRD module | Attitude towards illness QoL |
| Holroyd 1986[87] | USA | Cross-Sectional | To contribute to construct validity for the QRS and to a short form of the QRS by clarifying the meaning(s) of elevated scores | 11 | 11 | NR | 6 - 15 | 45.40 | 1 | Questionnaire on resources and stress (QRS) | Behavior/mental health |
| Huang  2022[88] | USA | RCT | To examine the effects of “ALL YOU NEED IS LOVE”, a novel six-week, self-directed patient education manual designed to improve CKD knowledge/self-management, health care transition readiness, self-advocacy, and mindfulness skills among adolescents with CKD | 49 | - | NR | 11 - 17 | Pt education only group: 48.4   Mindfulness-based pt education group: 61.1 | 4 | “ALL YOU NEED IS LOVE”-related Surveys  Child and Adolescent Mindfulness Measure (CAMM)  The Self-Management and Transition to Adulthood with Rx = Treatment (STARx) Questionnaire  Patient Self-Advocacy Scale (PSAS) | Behavior/mental health  Health literacy/knowledge  Transition/coping  Treatment experience Social well-being |
| Hsu  2023[89] | Taiwan | Cohort | To characterize longitudinal changes and correlations between EQ‑5D‑Y and generic PedsQL and their associations with clinical changes in children and adolescents with mild‑to‑moderate CKD | 121 | NR | Early CKD n=121 | 7 - 18 | 52 | 2 | EQ-5D-Y  PedsQL 4.0 | QoL |
| Imani  2021[90] | Uganda | Cross-Sectional | To describe the patterns of kidney disease in Ugandan Children and to evaluate the impact of CKD on QoL in children with CKD in comparison with that of children with benign or resolving kidney disease and assess for factors that might be associated with QoL impairment | 71 | 71 | NR | 0.2 - 18 | 62 | 1 | PedsQL 4.0 | QoL |
| Jalali-Farahani 2018[91] | Iran | Cross-Sectional | To compare QoL among healthy, obese and chronically ill children | 100 | 100 | NR | 8 - 12 | NR | 1 | PedsQL 4.0 (Iranian version) | QoL |
| Jawa  2022[92] | Canada | Cross-Sectional | To develop and refine a patient-reported symptom assessment tool for children with CKD/kidney failure (PRO-Kid), to evaluate the frequency and impact of symptoms | 11 | - | Early CKD n=7 HD n=1 PD n=3 | 8 - 18 | 45.50 | 1 | PRO-Kid | QoL |
| Johnson 2020[93] | USA and Canada | Cohort | To describe parent-reported emotional-behavioral functioning of children with CKD at baseline and examine emotional-behavioral functioning longitudinally as CKD progresses and determine which are associated with changes in emotional-behavioral functioning. | - | 845 | Early CKD  n=845 | 2 – 18 | 61 | 1 | BASC-2 PRS (Parent report scale) | Behavior/mental health |
| Kang  2019[94] | Korea | Cohort | To assess the mental health problems and psychosocial adjustment problems in children and adolescents with CKD in the KNOW-Ped CKD cohort, using the Korean-Child Behavior Checklist | 166 | NR | Early CKD n=166 | 3.8 - 16.6 | 70.50 | 1 | Korean-Child Behavioral Checklist (K-CBCL) | Behavior/mental health |
| Karava 2022[95] | Greece | Cross-sectional | To investigate the effect of CKD stage on fatigue and QoL in the pediatric population | 30 | - | Early CKD n=23 Tx n=7 | 7 - 18 | 63.30 | 2 | PedsQL-Multidimensional Fatigue scale (Greek translation)  PedsQL- Pediatric Quality of Life Inventory (Greek translation) | QoL Sleep |
| Kari  2014[96] | Saudi Arabia | Cross-sectional | To evaluate the social consequences of dialysis on children and their parents | 36 | 36 | HD n=17 PD n=19 | NR | 55.60 | 1 | Study-specific questionnaire on treatment, care experience, and stress | Behavior/mental health Treatment experience |
| Kasemy 2020[97] | Egypt | Cohort | To assess the effect of omega-3 Fatty acid supplements on QoL among children on dialysis and to evaluate its use regarding adequacy of dialysis and inflammatory markers. | 31 | NR | NR | 8 - 14 | 45.20 | 1 | PedsQL | QoL |
| Khalf-Allah 2024[98] | Egypt | RCT | To investigate the effect of muscle stretching and isometric exercises on QoL of children undergoing HD | 30 | NR | HD n=30 | 14 - 18 | 70 | 1 | PedsQL 4.0 (Arabic translation) | QoL |
| Khalil  2020[99] | Egypt | Cross-sectional | To analyze the psychosocial situation in families of children treated with renal dialysis and their mothers | 40 | 40 | NR | 6 - 17 | 55 | 1 | PedsQL 4.0 | QoL |
| Khanjari 2018[100] | Iran | Other | To investigate the effect of blended learning on the QoL of children with NS | 38 | NR | NS | 8 - 12 | 65.80 | 1 | PedsQL 4.0 (Persian version) | QoL |
| Khazaei 2021[101] | Iran | Before and after study | To investigate the effectiveness of a support-training program developed based on Orem’s self-care theory on QoL among Iranian children undergoing HD | 27 | NR | HD n=27 | 8 - 15 | NR | 2 | KIDSCREEN-52  Author-initiated self-care measurement scale | QoL Activities of daily living |
| Kilicoglu 2016[102] | Turkey | Case-control | To assess depression, anxiety, QoL in a cohort of children and adolescents with ESRD, to compare these findings with healthy controls, and to evaluate the association between these and ESRD | 32 | NR | Tx n=13 PD n=11 HD n=8 | 8 - 18 | 40.60 | 3 | PedsQL (Turkish translation)  Child Depression Inventory (CDI)  State–Trait Anxiety Inventories for Children (STAI-C) | Behavior/mental health QoL |
| Kilis-Pstrusinska 1994[103] | Poland | Cross-sectional | To investigate the perception of the family among children and adolescents with CRF treated conservatively and on HD | 27 | NR | Early CKD, HD | 9 - 18 | NR | 2 | Unstructured psychological interview  Cattell Youth self-assessment questionnaire | Behavior/mental health Social well-being |
| Kilis-Pstrusinska 2013[104] | Poland | Cross-sectional | To analyze psychosocial aspects of CKD in children treated with APD | 41 | 79 | PD n=41 | 2.1 - 18 | 65.85 | 2 | PedsQL 4.0  Caregiver burden scale | QoL Treatment experience |
| Kilis-Pstrusinska 2014[105] | Poland | Cross-sectional | To explore the selected elements of life situation of patients suffering with CKD as well as their parents, depending on the CKD stage and appropriate treatment | 203 | 388 | Early CKD n=137 HD n=25 PD n=41 | NR | 61 | 1 | Author-initiated family changes questionnaire | Social well-being |
| Knight  2017[106] | USA and Canada | Cross-sectional | To identify factors contributing to cognitive impairment in children with lupus nephritis | 171 | N/A | NR | 1 - 16 | 60 | 2 | BASC-2  PedsQL 4.0 | Behavior/mental health QoL |
| Kogon  2016[107] | USA and Canada | Cross-sectional | To assess depression in children with CKD and to determine associations with patient characteristics, intellectual and educational levels, and QoL | 344 | - | NR | 6 - 17 | 59 | 2 | Child depression inventory (CDI)  PedsQL 4.0 | Behavior/mental health QoL |
| Krasnoff 2006[108] | USA | Cross-sectional | To measure and compare physical fitness used in U.S. school systems (FITNESSGRAM) and to assess physical activity participation in pediatric Kidney and liver transplant recipients | 25 | NR | Tx n=25 | 12 - 18 | 64 | 1 | Previous Day Physical Activity Recall (PDPAR) | Physical activity |
| Krissberg 2024[109] | USA | Mixed-methods | To explore associations of food insecurity (FI) and CKD outcomes, and understand its role in following prescribed kidney diet recommendations | 56 | 12 | Tx n=18 | 12 - 18 | Pt with FI– 67   Pt without FI – 61 | 1 | Hunger vital sign | Food insecurity |
| Kul  2013[110] | Turkey | Cross-sectional | To compare the QoL of children and adolescents in various stages of CKD who were managed with different treatment modalities to that of children and adolescents without any chronic disease | 55 | 92 | Tx n=18 HD n=8 PD n=13 Early CKD n=37 | 8 - 18 | 60 | 1 | PedsQL | QoL |
| Kupferman 2020[111] |  | Cross-sectional | To determine stroke incidence and assess the association between stroke and neurocognitive functioning in children with CKD | 20 | - | NR | 1 - 16 | 45 | 1 | Behavior Rating Inventory for Executive Functioning (BRIEF) | Behavior/mental health |
| Lambe 2018[112] | UK | Cross-sectional | To present a method for estimating CHU-9D index scores from responses to the PedsQL™ using data from a RCT of prednisolone therapy for treatment of childhood corticosteroid-sensitive nephrotic syndrome. | 643 | NR | NS | 5 - 12 | 59-70% (varying between groups) | 2 | CHU-9D  PedsQL 4.0 | QoL |
| Lindeback 2024[113] | Australia | Cross-sectional | To compare nutritional intake and diet quality of Australian children with CKD to controls | 36 | NR | Early CKD n=16 Tx n=20 | 2 - 18 | 69.40 | 2 | Australian Eating Survey Child and Adolescent  Australian Child and Adolescent Recommended Food Score (ACARFS) | Diet/nutrition |
| Lopes  2014[114] | Brazil | Cross-sectional | To describe the QoL of children and adolescents with pre-dialysis or dialysis/transplantation, as well as their caregivers, and compare this to that of a healthy control group | 64 | 64 | Early CKD n=21 PD n=13 HD n=12 Tx n=18 | 2 - 18 | 55 | 2 | PedsQL 4.0  Short form-36 (SF-36) | QoL |
| Lopes  2011[115] | Brazil | Cross-sectional | To translate into and adapt to Brazilian Portuguese the Peds QL TM - ESRD version 3.0 questionnaire. | 15 | 20 | Early CKD n=3 HD n=3 PD n=4 Tx n=5 | 2 - 18 | 66.67 | 1 | PedsQL 3.0 ESRD module (Brazilian Portuguese version) | QoL |
| Lopes  2015[116] | Brazil | Cross-sectional | To evaluate the reliability of the translation and cultural adaptation of the PedsQL 3.0 ESRD module using children and parents reports in Brazilian children with ESRD | 24 | 32 | Early CKD n=2 HD n=24 PD n=2 Tx n=4 | 3 - 18 | 56.25 | 1 | PedsQL 3.0 ESRD module (Brazilian Portugese version) | QoL |
| Lui  2020[117] | Canada | Cross-sectional | To assess the physical activity levels in a single-center pediatric solid organ transplant population and to identify baseline and contemporaneous factors that may play a contributing role to their level of physical activity | 29 | NR | Tx n=29 | 12 - 18 | 58.60 | 1 | Physical activity questionnaire (PAQ) (PAQ-C and PAQ-A) | Physical activity |
| Lui  2021[118] | Canada | Cross-sectional | To assess whether overall QoL was associated with physical activity and to determine whether that association was independent of other baseline and contemporaneous clinical and demographic indicators | 27 | NR | Tx n=27 | 12 - 18 | 63 | 3 | PedsQL 4.0  Pediatric Quality of Life Inventory 3.0 Transplant Module (PedsQL- TM)   Physical Activity Questionnaire for Children (PAQ- C; ages 8– 12 years) and Adolescents (PAQ- A; ages 13– 18 years) | QoL Physical activity |
| Madden 2002[119] | UK | Cross-sectional | To explore maternal and child perspectives on children’s adjustment in the context of pediatric CKD, and maternal psychological variables that may account for variance in child and maternal ratings | 43 | 43 | PD n=7 Tx n=19 Early CKD n=17 | 11 - 16 | 60.46 | 1 | Strengths and difficulties questionnaire (SDQ) | Behavior/mental health |
| Maikranz 2007[120] | USA | Cohort | To examine the associations among hope, illness-related uncertainty, anxiety, depression, and adherence in a sample of children with renal and liver transplantations | 28 | 28 | NR | 7 - 18 | 51.40 | 6 | Adult Trait hope scale  Children's hope scale  Parent’s Perception of Uncertainty in Illness Scale (PPU)  Child Uncertainty in Illness Scale (CUIS)  Behavioral Assessment Scale for Children, Parent Report Form (BASC-PRF)  Behavioral Assessment Scale for Children, Self-Report of Personality (BASC-SRP)  Children’s Depression Inventory (CDI)  State-Trait Anxiety Inventory for Children (STAIC)  Adolescent or caregiver self-report of child adherence (SRA) | Behavior/mental health Treatment adherence |
| Marciano 2011[121] | Brazil | Cross-sectional | To investigate the prevalence of behavioral disorders and to assess the QoL in patients with CKD | 136 | 136 | Early CKD n=75 Dx n=39 Tx n=22 | 2 - 18 | 56 | 2 | Strengths and difficulties questionnaire (SDQ)  PedsQL4.0 | Behavior/mental health QoL |
| Matsuda-Abedini 2024[122] | Canada | Cross-sectional | To externally validate PRO-Kid in a Canadian cohort of children with CKD/kidney failure | 100 | NR | Early CKD, HD, PD | 8 - 18 | 63 | 2 | PRO-KID  PedsQL 4.0 | QoL Symptoms |
| Mavis  2015[123] | Canada | Cross-sectional | To identify patient and family patterns of adaptation among kidney and liver transplant recipients regarding vulnerability, impact of illness on the family, family functioning, and QoL | 24 | 24 | Tx n=24 | 5 - 18 | 67 | 3 | Child Vulnerability Scale  Functional Status II-R   PedsQL 4.0 | Family/social relationships  QoL |
| McKenna 2006[124] | Canada | Cross-sectional | To compare QoL in children with different stages of CKD to each other and to a control population | 64 | NR | Early CKD n=20 Dx n=17 Tx n=27 | 2 - 18 | Early CKD: 65  Dx: 47  Tx: 14 67 | 1 | PedsQL 4.0 | QoL |
| Medynska 2017[125] | Poland | Cross-sectional | To analyze the selected psychosocial aspects of CKD in children treated with HD | 25 | 50 | HD n=25 | 2 - 18 | 40 | 1 | PedsQL 4.0 | QoL |
| Moreira 2015[126] | Brazil | Cross-sectional | To investigate resilience, QoL, anxiety and depressive symptoms in children and adolescents with pre-dialysis CKD and compare these to healthy controls | 28 | NR | Early CKD n=28 | 9 - 18 | 64.30 | 4 | The Wagnild and Young Resilience Scale  PedsQL 4.0  Child Depression Inventory (CDI)  Self-report for Childhood Anxiety Related Disorders (SCARED) | Behavior/mental health QoL |
| Neul  2013[127] | USA | Cohort | To describe longitudinally observed data examining both global and disease-specific aspects of QoL based on patient self- and parent proxy reports in a cohort of dialysis-dependent children with ESRD and investigate potential associations with demographic and medical variables of interest | 53 | - | HD n=27 PD n=26 | 2 - 18 | 60.40 | 2 | PedsQL 4.0    PedsQL3.0 ESRD Module | QoL |
| Ng  2020[128] | USA and Canada | Cohort | To describe participant-reported renal replacement therapy planning discussions by CKD stage, clinical and sociodemographic characteristics, in the CKD in Children cohort | 721 | NR | Early CKD n=721 | 0.5 - 15 | 61.90 | 1 | “In the past year, have you discussed renal replacement therapy with your doctor or healthcare provider?” | Treatment experience |
| Palupi-Baroto 2023[129] | Indonesia | Cross-sectional | To assess QoL in children with CKD in a national tertiary hospital in Indonesia | 82 | 82 | Early CKD n=62 HD n=10 PD n=10 | 8 - 18 | 58.50 | 1 | PedsQL 4.0 | QoL |
| Pardede 2019[130] | Indonesia | Cross-sectional | To evaluate the QoL in children with CKD in the association with demographic factors, duration of diagnosis, and disease severity | 112 | NR | NR | 2 - 18 | 53.60 | 1 | PedsQL 4.0 | QoL |
| Park  2012[131] | South Korea | Cross-sectional | To assess QoL in children and adolescents with ESRD using the Korean version of PedsQL ESRD | 92 | 92 | HD n=11 PD n=44 Tx n=37 | 2 - 18 | 47.80 | 1 | PedsQL 3.0 ESRD Module (Korean version) | QoL |
| Pattaragarn 2004[132] | USA | Cross-sectional | To evaluate the correlation between exercise capacity and hemoglobin in pediatric patients with end-stage renal ESRD treated with APD and HD | 24 | NR | HD n=9 PD n=15 | 11.5 - 18 | 66.60 | 1 | Physical Activity Questionnaire for Older Children (PAQ-C) | Physical activity |
| Qvist  2004[133] | Finland | Cross-sectional | To assess the impact of Tx in early childhood on the children’s psychosocial adjustment and QoL | 34 | NR | Tx n=34 | 8 - 11 | 69 | 2 | Child behavior checklist (CBCL)  17D | Behavior/mental health QoL |
| Rahman 2016[134] | Bangladesh | Cross-sectional | To assess the influence of demographic characteristics on QoL and to evaluate the use of QoL measures for the management in NS in children | 50 | 50 | NS | 2 - 12 | 58.00 | 1 | PedsQL 4.0 (Bengali translation) | QoL |
| Reeve  2016[135] | USA | Other | To evaluate the responsiveness of eight PROMIS Pediatric measures in diverse samples of children and adolescents with cancer, sickle cell disease, and NS | 127 | NR | NS | 8 - 17 | 65 | 8 | PROMIS Pediatric measures of:  pain interference, mobility, upper extremity, fatigue, depressive symptoms, anxiety, anger, and peer relationships | Behavior/mental health  Fatigue  Physical activity Social well-being  Symptoms |
| Reynolds 1991[136] | UK | Cross-sectional | To compare the psychosocial adjustment after translation with their adjustment before and during HD with comparable measures | 73 | 67 | early CKD n=22 HD n=22 Tx n=29 | NR | NR | 3 | Birleson depression inventory  Lipsitt self-concept scale  Rutter A behavioral questionnaire | Behavior/mental health |
| Riar  2013[137] | USA | Cross-sectional | To compare the point prevalence of restless leg syndrome in children with CKD to healthy controls | 124 | NR | NR | 8 - 18 | 64.50 | 3 | Pediatric Daytime Sleepiness Scale (PDSS)  PedsQL 4.0  Restless leg syndrome (RLS) diagnostic questionnaire | QoL Sleep  Symptoms |
| Roccella 2005[138] | Italy | Cross-sectional | To evaluate the impact of CKD on QoL of children and adolescents in terms of physical, psychological and social consequences, considering parent's perceptions regarding their child's illness | 47 | 94 | Dx n=5 Tx n=6 | 2 - 18 | 63.80 | 1 | Childhood illness impact scale | QoL |
| Rodriguez Cuellar 2019[139] | Mexico | Cross-sectional | To evaluate depression in a sample of pediatric patients with various RRT and impact on depression scores | 47 | NR | Tx n=8 HD n=7 PD n=6 | NR | 45 | 1 | Children's depression inventory (CDI) | Behavior/mental health |
| Rotella 2020[140] | Brazil | Mixed method | To investigate the emotional repercussions and QoL associated with ESKD in children and adolescents undergoing HD or a kidney Tx | 48 | 48 | HD n=24 Tx n=24 | 5 - 18 | 45.83 | 1 | PedsQL 4.0 | QoL |
| Roumelioti 2010[141] | USA and Canada | Cross-sectional | To examine the extent to which sleep, and fatigue symptoms were associated with poorer QoL | 301 | NR | Early CKD n=301 | 1 - 16 | 61 | 2 | PedsQL 4.0  CkiD symptoms list (adapted from the chronic renal insufficiency cohort (CRIC) study | QoL Symptoms |
| Roussel 2019[142] | France | Cross-sectional | To describe QoL in idiopathic nephrotic syndrome | 110 | NR | NS | 7 - 17 | NR | 1 | KINDL (French version) | QoL |
| Scheuerman 2017[143] | Israel | Cross-sectional | To investigate annual influenza vaccination rates in children with CKD and identified reasons for non-immunization. | 217 | NR | NR | 0.5 - 18 | 65 | 1 | Immunization adapted questionnaire | Health behaviors/Preventative health |
| Selewski 2014[144] | USA | Cross-sectional | To evaluate the sensitivity of the PROMIS pediatric instrument to indicators of disease severity and activity in pediatric CKD | 229 | 229 | Early CKD n=144 Tx n=85 | 8 - 17 | 61.10 | 7 | PROMIS pediatric measures of depression, anxiety, social-peer relationships, pain interference, fatigue, mobility, upper extremity function | Behavior/mental health  QoL  Mobility Social well-being Symptoms |
| Selewski 2015[145] | USA and Canada | Cohort | To compare the differences in QoL in children with prevalent versus incident NS to investigate the impact of disease duration on QoL as measured by the PROMIS measures | 127 | NR | NS | 8 - 17 | 65 | 8 | PROMIS pediatric measures of depression, anxiety, social-peer relationships, pain interference, fatigue, and mobility  PedsQL 4.0 | Behavior/mental health QoL  Mobility Social well-being Symptoms |
| Selewski 2017[146] | USA and Canada | Other | To longitudinally validate the PROMIS measures in pediatric NS | 127 | NR | NS | 8 - 17 | 65 | 8 | PROMIS pediatric measures of depression, anxiety, social-peer relationships, pain interference, fatigue, and mobility  PedsQL 4.0  Author initiated global assessment of change for nephrotic syndrome and overall health | Behavior/mental health  Health status QoL  Mobility Social well-being  Symptoms |
| Senses Dinc 2019[147] | Turkey | Cohort | To evaluate the psychiatric morbidity in children with CKD in terms of different treatment modalities in comparison to healthy peers | 66 | NR | Tx n=21 Dx n=27 Conservative n=18 | 8 - 18 | 59.10 | 2 | Children's depression inventory (CDI)  State-Trait Anxiety Inventory for Children (STAIC) | Behavior/mental health |
| Shingde 2024[148] | Australia and New Zealand | Cohort | To assess the longitudinal association between SEP and the carer’s rated health of the child with CKD and the carers’ self-rated health | 375 | 375 | Early CKD n=199 Dx n=42 Tx n=134 | 6 - 18 | 62 | 1 | Carer-rated child health score | Overall health/health status |
| Silva  2019[149] | Brazil | Cross-sectional | To evaluate if health and oral health status of children and adolescents with different stages of CKD are associated with their QoL), oral QoL and socioeconomic and demographic conditions | 100 | 100 | NR | 8 - 18 | 67 | 2 | Both translated and validated for Brazilian culture  PedsQL 4.0   Peds QL ™—Oral Health Scale (designed to be used simultaneously with PedsQL) | QoL Oral health |
| Sinha  2009[150] | Canada | Cross-sectional | To characterize the prevalence of sleep disorders among children and adolescents with non-dialysis-dependent CKD | 49 | NR | Tx n=19 Early CKD n=30 | 6 - 18 | 71.43 | 1 | Pediatric sleep questionnaire (PSQ) | Sleep |
| Snethen 2004[151] | USA | Cross-sectional | To identify coping strategies specific to adolescents with ESRD | 35 | NR | Tx n=24 Dx n=11 | 13 - 18 | 57.14 | 1 | A-COPE | Behavior/mental health  Transition/coping  Social well-being |
| Stabouli 2019[152] | Greece | Case-control | To assess the prevalence of sleep-related disorders in CKD pediatric patients, and to examine possible correlations with measures of executive function | 51 | NR | Early CKD n=51 | 5 - 18 | 57 | 2 | Pediatric Seep Questionnaire (PSQ) (Greek version)  Behavior Rating Inventory of Executive Function (BRIEF) (Greek version) | Behavior/mental health Sleep |
| Stahl  2022[153] | USA and Canada | Cross-sectional | To examine key psychiatric diagnoses in children with CKD compared with those in the general pediatric population and assess the correlation between parent-reported diagnosis and self-reported symptoms of depression | 875 | NR | Early CKD n=875 | 2 - 17 | 62 | 1 | Children's Depression Inventory (CDI) | Behavior/mental health |
| Sugiarto 2019[154] | Indonesia | Cross-sectional | To assess for potential associations between CKD and emotional/behavioral disorders in adolescents | 75 | NR | Early CKD n=64 HD/PD n=11 | 10 - 18 | 41 | 1 | Strengths and difficulties questionnaire (SDQ) | Behavior/mental health |
| Sundaram 2007[155] | USA | Cross-sectional | (i) To quantify QoL in adolescent liver and kidney transplant patients, (ii) compare caregiver ratings of their children’s QoL to adolescent self-reports and (iii) examine the relationship between QoL and disease specific disability | 54 | n=18 | Tx n=18 | 11 - 18 | 67 | 1 | Child Health Questionnaire-Child Form 87 (CHQ-CF 87)  Child Health Questionnaire-Parent Form 50 (CHQ-PF 50) | QoL |
| Tain  2023[156] | Taiwan | Cohort | To assess the degree of agreement between child self- and parent proxy-rated QOL and to identify factors associated with discordance at baseline and during follow-up in Taiwanese children with CKD | 112 | 112 | NR | 5 - 18 | 53.46 | 1 | PedsQL 4.0 | QoL |
| Tan  2021[157] | Australia | Cross-sectional | To investigate factors contributing to the impact of caring for a child with posterior urethral valves and to determine the extent to which families view their care as family-centered, and whether this can affect the impact of care | 31 | 31 | Early CKD n=31 | 0 - 17 | NR | 1 | Measure of Processes of Care-20 (MPOC-20) | Treatment experience |
| Teixeira 2014[158] | Brazil | Cross-sectional | To evaluate the impact of the CKD on QoL, from the children's and their parents’ perspective, respiratory muscle strength, lung function, and functional capacity in children and adolescents | 40 | NR | Early CKD n=13 PD n=9 HD n=10 Tx n=8 | 8 - 17 | 52.50 | 1 | PedsQL questionnaire (version 3.0) | QoL |
| Tiwari  2015[159] | India | Cross-sectional | To assess the QoL of children with ESRD on maintenance dialysis and following KTP & to compare the QoL assessed by child self-report and parent’s proxy report | 55 | NR | Dx n=27 Tx n=28 | 5 - 18 | NR | 1 | PedsQL 3.0 ESRD Module (Hindi version) | QoL |
| Troost 2019[160] | USA | Cohort | To use a latent profile analysis to define clinically distinct QOL profiles in patients with NS | 161 | NR | NS n=161 | 8 – 18 | PROMIS-II: 65  NEPTU-NE: 60 | 6 | PROMIS pediatric measures of fatigue, pain interference, anxiety, depression, mobility and peer relationships | Behavior/Mental Health  Fatigue QoL Mobility Social well-being |
| Tsampalieros  2019[161] | Canada | Cross-sectional | To explore associations between obstructive sleep apnea (OSA) severity using the apnea-hypopnea-index and obstructive apnea-hypopnea-index on polysomnography, OSA symptoms, and measures of hypertension in children with CKD | 19 | NR | Early CKD n=19 | 6 - 18 | 57.90 | 2 | Pediatric Sleep Questionnaire (PSQ)  Modified Epworth Sleepiness Scale | Sleep |
| Velasco  2020[162] | Argentina | Cross-sectional | To remedy that gap in understanding by determining the QoL of siblings of Kidney Tx recipients (KTR) and comparing them with the QOLs of both KTRs and a healthy control group | 43 | NR | Tx n=43 | 8 - 18 | 49 | 1 | KIDSCREEN-52 (Argentinian version) | QoL |
| Veltkamp 2022[163] | Amsterdam | Cross-sectional | To describe and assess the implementation and use of the KLIK PROM portal in the pediatric nephrology department | 112 | NR | NR | 8 - 18 | 57 | 2 | TNO-AZL Preschool Children Quality of Life (TAPQOL)  PedsQL 4.0 | QoL |
| Verhofste 2024[164] | USA | Cross-sectional | To describe patient-reported self-concept among children with CKD and evaluate the relationship between self-concept and academic performance | 23 | NR | Early CKD | 6 - 16 | 100 | 1 | Self-Description Questionnaire | Social well-being |
| Voet  2024[165] | Netherlands | Mixed methods | To explore young children's and their parents’ experiences during research participation in the context of an intensive medical treatment | 9 | 9 | Tx n=14 | NR | 44 | 1 | DISCOmfort in Research with Children (DISCO-RC) | Experience of medical treatments in research |
| Wang  2023[166] | China | Cohort | To investigate the impact of continuous home care on nursing compliance, immune function, and QoL among pediatric patients diagnosed with NS | 98 | NR | NS | 9 - 18 | 68 | 3 | The Knowing, Believing, and Acting survey  Self-developed nursing compliance scale  The concise health status survey scale | QoL  Overall health/health status  Treatment adherence |
| Webb  2019[167] | UK | RCT | To determine whether extending initial prednisolone treatment from 8 to 16 weeks in children with idiopathic steroid sensitive nephrotic syndrome improves the pattern of disease relapse | 237 | NR | Early CKD n=237 | 1 - 17 | 65 | 3 | Child behavior checklist (CBCL)  Child health utility 9 dimension (CHU-9D)  PedsQL 4.0 | Behavior/mental health QoL |
| Weigmann-Fasbender 2020[168] | Germany | Cohort | To evaluate cardiorespiratory fitness, motor coordination, muscle strength, daily physical activity, and QoL and to find out, if active video gaming is effective for improving these items in this patient population | 33 | NR | Tx n=33 | NR | 24.24 | 1 | PedsQL 4.0 | QoL |
| Wong  2016[169] | USA and Canada | Cross-sectional | To address this gap in the literature by determining if the presence of elevated blood pressure, antihypertensive medication use, and/or duration of hypertension were associated with decreased QoL in children with mild to moderate CKD | 551 | NR | Early CKD n=551 | 1 - 16 | 62 | 1 | PedsQL 4.0 | QoL |
| Wong  2024[170] | Australia | RCT | To evaluate the effectiveness of a patient navigation program in children/adolescents with CKD from socioeconomically disadvantaged backgrounds or rural and/or remote areas, and to describe caregivers’ perspectives on experiences of the program | 162 | 162 | Tx n=42 PD n=8 HD n=3 Early CKD n=109 | 0 - 16 | 64.80 | 2 | Self-Rated Health (SRH)  Health Utilities Index Mark III (HUI-3) | QoL Overall health/health status |
| Yadav  2017[171] | India | Cross-sectional | To examine the multidimensional aspects of QOL after renal transplant and to find the physical, psychosocial, and economic issues that pediatric transplant patients and their parents face especially in context to Indian society | 62 | NR | Tx n=62 | 5 - 18 | 71 | 2 | WHOQOL- BREF questionnaire  Author-initiated child/parent-reported assessment of the child’s behavioral and emotional status | Behavior/mental health  QoL |
| Yang  2024[172] | Australia | Cross-sectional | To define the prevalence and typology of sleep disorders in children and adolescents after kidney transplantation using the International Classification of Sleep Disorders, and to identify predictors of sleep disorders | 35 | 42 | Tx | 2 - 18 | 48.50 | 5 | The Sleep Disturbance Scale for Children (SDSC)  Questions adapted from the Children’s Report of Sleep Patterns  Questions about restless leg syndrome (RLS) symptoms, derived from standard criteria for RLS diagnosis in children and adolescents  Epworth Sleepiness Scale for Children and Adolescents (ESS-CHAD)  Author-initiated Nocturnal Medical Needs Impacting Sleep questionnaire | Health literacy/knowledge  Sleep Symptoms |
| Youssef 2015[173] | Egypt | Cohort | To evaluate the impact of nutritional knowledge following interventional educational sessions in chronic Dx patients | 40 | NR | Early CKD n=40 | 2 - 18 | 42.50 | 1 | Pre-designed questionnaire for assessment of nutritional knowledge | Nutrition knowledge |
| Yurtdas Depboylu 2023[174] | Turkey | Cross-sectional | To determine the dietary acid load of children with CKD and to evaluate the relationship between dietary acid load, nutritional status, and QoL | 67 | 67 | Early CKD n=67 | 3 - 18 | 52 | 1 | PedsQL | QoL |
| Zelikovsky 2008[175] | USA | Cross-sectional | To examine the potentially modifiable barriers related to adherence among adolescent kidney transplant candidates | 56 | NR | HD n=11 PD n=4 (all pts listed for a kidney Tx) | 11 - 18 | 73.20 | 1 | Medication adherence measure (MAM) | Medication adherence |
| Note: ADOS = Autism Diagnostic Observation Schedule, AE = Adverse Event, APD = Automated Peritoneal Dialysis, AUQEI = Autoquestionnaire Qualité de Vie Enfant Imagé, BAASIS = Basel Assessment of Adherence to Immunosuppressive Medications Scale, BASC-2 = Behavior Assessment System for Children, Second Edition, BDI = Beck Depression Inventory, BRIEF = Behavior Rating Inventory of Executive Function, CAKUT = Congenital Anomalies of the Kidney and Urinary Tract, CBCL = Child Behavior Checklist, CDI = Children’s Depression Inventory, CHIP-AE = Child Health and Illness Profile - Adolescent Edition, CHQ-CF 87 = Child Health Questionnaire - Child Form 87, CHQ-PF50 = Child Health Questionnaire - Parent Form 50, CKD = Chronic Kidney Disease, COSMIN = COnsensus-based Standards for the selection of health Measurement INstruments, CRF = Chronic Renal Failure, Dx = Dialysis, EQ-5D-Y = EuroQol Five Dimensions Youth version, ESRD = End-Stage Renal Disease, ESS = Epworth Sleepiness Scale, ESS-CHAD = Epworth Sleepiness Scale for Children and Adolescents, FI = Food Insecurity, FRI = Family Relationship Index, GCQ = Generic Children's Quality of Life Measure, HD = Haemodialysis, QoL = Health-Related Quality of Life, HUI = Health Utilities Index, HUI3 = Health Utilities Index Mark 3, K-CBCL = Korean Child Behavior Checklist, KINDL = Kinder Lebensqualität Fragebogen, MD = Maintenance Dialysis, MPOC-20 = Measure of Processes of Care-20, MTSOSD-59R = Modified Transplant Symptom Occurrence and Symptom Distress Scale, N/a = Not applicable, NR = Not reported NS = Nephrotic Syndrome, PAQ = Physical Activity Questionnaire, PAQ-A = Physical Activity Questionnaire for Adolescents, PAQ-C = Physical Activity Questionnaire for Children, PCFES = Perceived Collective Family Efficacy Scale, PedsQL = Pediatric Quality of Life Inventory, PedsQL-TM = Pediatric Quality of Life Inventory - Transplant Module, PREM = Patient-Reported Experience Measure, PROM = Patient-Reported Outcome Measure, PROMIS = Patient-Reported Outcomes Measurement Information System, PR-CBS = Pediatric Renal Caregiver Burden Scale, PSAS = Patient Self-Advocacy Scale, PSQ = Pediatric Sleep Questionnaire, PUV = Posterior Urethral Valves, QoL = Quality of Life, RCT = Randomized Controlled Trial, RLS = Restless Leg Syndrome, SDQ = Strengths and Difficulties Questionnaire, SEP = Socioeconomic Position, SRA = Self-Report of Adherence, SRH = Self-Rated Health, SS = Short Stature, STAIC = State-Trait Anxiety Inventory for Children, STAI = State-Trait Anxiety Inventory, TECAVNER = Test of Quality of Life in Children with Kidney Disease, TRAQ = Transition Readiness Assessment Questionnaire, Tx = Transplant, UNC TRxANSITION Scale = University of North Carolina Transition Scale, VAQOL = Visual Analogue Quality of Life, VSP-A = Vécu et Santé Perçue de l’Adolescent, YSR = Youth Self-Report. | | | | | | | | | | | |

**Table S4. Detailed summary of psychometric properties of PRMs which were validated in the included studies**

| **Study details** | | | | | | **COSMIN psychometric properties based on included studies** | | | | | | | | | | |
| --- | --- | --- | --- | --- | --- | --- | --- | --- | --- | --- | --- | --- | --- | --- | --- | --- |
|  |  |  |  |  |  | **Validity** | | | | | | **Reliability** | | | |  |
| **PRM + study** | **Who reports?** | **Response  format** | **Items** | **Validated in general population** | **Developed for CKD** | **Content Validity** | **Convergent validity** | **Construct validity** | **Known groups validity** | **Structural validity** | **Criterion validity** | **Reliability (Test-retest, inter-rater, and intra-rater)** | **Internal consistency** | **Measurement  error** | | **Responsiveness** |
| **QoL** | | | | | | | | | | | | | | | | |
| 16D[81] | Participant (or proxy) | 5  ordinal levels on each dimension | 16 | Y | N | - | - | - | - | - | - | - | Internal consistency reliability for the 16D was 0.86 | - | - | |
| 17D[81] | Participant (or proxy) | 5 ordinal levels on each dimension | 17 | Y | N | - | - | - | - | - | - | - | Internal consistency reliability for the 17D was 0.72. | - | - | |
| EQ-5D-Y[36] | Participant (or proxy) | Each dimension has three levels, representing severity: no problems, some problems and a lot of problems. The instrument also includes a visual analogue scale. | 16 | Y | N | - | - | - | - | - | - | “This was based on a subset of children who completed two EQ-5D-y assessments (n=53).  Cohen’s κ value for mobility (0.79) and self-care (0.66) dimensions were in the high level of agreement (98.1%) while usual activities (0.2) were within fair agreement (88.7%). The pain/discomfort (0.01) and anxiety/depression (-0.15) dimensions fell within slight to no agreement (77.4% and 67.9%)  The ICC value for mean EQ VAS value was 0.47 (0.08–0.69).” | - | - | - | |
| KIDSCREEN-52[101] | Participant | 5-point Likert scale | 52 | Y | N | - | - | - | - | - | - | - | “Cronbach’s alpha coefficients  for the subscales were 0.89 (physical), 0.77 (emotional), 0.76 (social), 0.82 (academic functioning), and 0.79 (overall QoL).” | - | - | |
| MOS-SF-20 (20-item Medical Outcomes Study)[16] | Participant or proxy | Varying scales – 3-to-5-point ordinal scales | 20 | Y | N | - | - | - | - | - | - | - | “Cronbach’s alpha: 0.8 (patients), 0.84 (parents) indicating good internal consistency.” | - | - | |
| PedsQL 4.0[20, 23, 43, 71, 74, 122, 124, 146, 149, 156] | Participant and/or proxy (depending on age) | 5-point likert scale | 23 | Y | N | - | - | **Bakar 2023: “**Positive Spearman rank correlations across all domains supports the construct validity of the translated version.” **Matsuda-Abedini 2024:** “Pearson correlations between PRO-Kid and PedsQL scores were strong: R −0.78 (95% CI, −0.85 to −0.70) for the frequency score and R −0.69 (95% CI, −0.78 to −0.56) for the impact score, reflecting poorer quality-of-life association with higher symptoms burden.  **Goldstein 2008: “**The majority of intercorrelations were in the medium to large range, supporting construct validity. Confirmatory factor analysis (CFA) fit indices indicated that the hypothesized 7-factor model had acceptable fit for child self-reports (CFI 0.94, RMSEA 0.062, NNFI 0.93) and parent-proxy reports (CFI 0.95, RMSEA 0.077, NNFI 0.94), further validating the construct.  Higher PedsQL ESRD Module Scale scores significantly correlated with higher Generic Core Total Scale scores, indicating that more severe ESRD-specific symptoms were associated with more impaired generic HRQoL. These findings, along with CFA results, strongly support the construct validity of the PedsQL ESRD Module.  **Selewski 2017: “**Correlations between changes in PROMIS and PedsQL scores across similar domains were generally strong. For instance, PROMIS Mobility correlated with PedsQL Physical Functioning (r = 0.60), and PROMIS Anxiety showed a negative correlation with PedsQL Anxiety (r = −0.68), indicating higher PROMIS Anxiety was associated with worse emotional functioning in PedsQL, as expected. However, PROMIS Peer Relationships did not exhibit a significant correlation with PedsQL Social Functioning (r = 0.11).” | **De Bruyne 2022:** “The known-groups validity of the general QoL measure was supported for parent proxy-reports, with parents of children with CKD rating lower QoL scores (67.0 ± 18.7) compared to healthy children (86.2 ± 13.3; p < 0.001) across all scales, except Social Functioning (p < 0.05). For child self-reports, only School Functioning showed significantly lower scores for CKD patients (67.8 ± 16.1) compared to healthy children (76.5 ± 15.8; p < 0.001), with no significant differences in the other scales.” | - | **Selewski 2017: “**The PedsQL Physical Functioning domain showed the strongest relationship to global assessment of change in nephrotic syndrome and overall health (\|d\|=0.65 and 0.72 respectively).” | **Bakar 2023:**  “Scores obtained from Generic 4.0 scales correlated with the disease-specific ESRD 3.0 scale, Spearman’s rho = 0.32, p = 0.007.”  **McKenna 2006: “**The ICC coefficient between parent and child reports was 0.63 - indicating a modest agreement on PedsQL scores between children and their parents.  **Silva 2019: “**Intra-examiner reliability was determined by the re-evaluation of 20 children and adolescents within a two-week interval (10% of the sample) and by means of Kappa values: 0.9 for decayed, missing, and filled teeth index; 0.8 for plaque index and gingival index system and 1.0 for developmental enamel defect index  **Tain 2023: “**The magnitude of discordance varied depending on the PedsQL domain. At initial assessment, the ICC showed higher agreements (> 0.70) in the total, physical health, and psychosocial health scores in child–parent dyads involving children aged 5–7 years than other age groups. The ICC for all domains of the PedsQL were low (< 0.5) for agreement in the child–parent dyads involving children aged 7–12 years, and the ICC for psychosocial health had the lowest agreement (ICC 0.42; 95% CI 0.25, 0.56), mainly due to discordance in social functioning (ICC − 0.12; 95% CI − 0.38, 0.16) in the child–parent dyads for adolescents (13–18 years)  In this study’s cohort of childhood CKD, the level of agreement between the HRQoL reports by patients and their parent proxies was consistently moderate–good for physical health, and poor for the emotional and social functioning domains of the PedsQL at baseline and during follow-up.  The agreement between self and proxy reports slightly improved in the follow-up in the physical, social, and school domains. In general, development of growth retardation, MBD/anemia, and proteinuria were most likely to be associated with increased discordance between child and parent proxy reports of physical or school domain of PedsQL during follow-up. On average, parent proxies reported lower HRQoL scores than children/adolescents themselves both at T1 and T2.” | **Azukaitis 2023:** “High in all proxy-reported PROM questionnaires (Cronbach alpha > 0.9), except for the 2–4-year-old group (0.631).”  **De Bruyne 2022: “C**ronbach’s alpha coefficients for Total Scale scores exceeding the 0.70 standard for group comparisons in both child self-reports and parent proxy-reports, demonstrating excellent reliability.  ICR analyses showed α’s ranging from 0.79 to 0.92 (high acceptable to excellent) for parent proxy-reports and from 0.62 to 0.83 (questionable to good) for child self-reports.  **Gerson 2010:**  “Internal consistency measured across PedsQL domains for parents and children separately, with additional analyses for toddler and school-age groups with CKD. The standardized alphas for parent proxies of overall HRQoL were 0.91 (toddlers) and 0.89 (school-age youth), and 0.92 for child self-reports. The lowest alpha (0.67) was found in the parent proxy for School Functioning in toddlers.  **Goldstein 2008: “**The PedsQL 4.0 Generic Core Scales demonstrated internal consistency reliabilities exceeding the 0.70 standard for child self-reports and parent-proxy reports, with total scale scores meeting or nearing the 0.90 threshold recommended for individual patient analysis, making them suitable as a summary score for primary HRQoL analysis in children with ESRD” | - | - | |
| PedsQL 3.0 - ESRD[23, 43, 74, 116] | Participant and/or proxy (depending on age) | 4-point Likert scale | 34 | Y | Y | - | - | - | **De Bruyne 2022: “**The construct validity of the PedsQL™ 3.0 ESRD Module was supported by intercorrelations between its 7 subscales and the 5 scales of the PedsQL™ 4.0 Generic Core Scales. For parent proxy-reports, most intercorrelations were medium (0.30–0.49) to large (>0.50), except a few small intercorrelations, with the highest being Family and Peer Interactions with Psychosocial Health (r = 0.73). For child self-reports, intercorrelations were predominantly medium to large, with small intercorrelations noted for School Functioning, Physical Health, and Social Functioning with specific ESRD subscales. Significant correlations comprised 28 out of 35 for parent proxy-reports and 31 out of 35 for child self-reports.” | - | - | **Bakar 2023: *Inter-rater reliability:*** “Responses from the child self-report group and the parent proxy group demonstrated good to excellent agreement for all the domains (ICC 0.70-0.91) except for the “Treatment” domain, where the ICC was 0.68.”  ***Reliability:*** “Scores obtained from Generic 4.0 scales correlated with the disease-specific ESRD 3.0 scale, Spearman’s rho = 0.32, p = 0.007.” | **Bakar 2023: “**Evaluation of the PedsQL 3.0 ESRD Module Bahasa Melayu version demonstrated good internal consistency (Cronbach alpha 0.82)”  **De Bruyne 2022: “C**ronbach’s alpha coefficients for Total Scale scores exceeding the 0.70 standard for group comparisons in both child self-reports and parent proxy-reports, demonstrating excellent reliability.  For the parent proxy-reports, subscale ICR analyses yielded αs ranging from 0.60 (questionable) to 0.94 (excellent), while patient self-reports showed αs between 0.62 (questionable) and 0.85 (good), with an unacceptable α of 0.47 for Perceived Physical Appearance.”  **Goldstein 2008:** “For  the PedsQL 3.0 ESRD Module, 6 of 7 parent proxy  report scales and 4 of 7 child self-report  scales exceeded the minimum   coefficient standard  of 0.70.”  **Lopes 2015: “**The overall rating of Cronbach´s alpha values resulted in values of 0.81 and 0.71 in patients and PC reports, respectively, demonstrating good internal consistency” | - | - | |
| TECAVNER (Test of Quality of Life in Children with kidney disease)[17] | Participant or proxy | 14 domains, some of which comprise various items or questions | varying |  | Y | - | - | - | - | - | - | - | “Cronbach Alpha Coefficient is 0.925 in the child self-report and 0.9156 in the parent proxy-report; extremely reliable with a high internal consistency across all domains.” | - | - | |
| **Health status/ symptoms** | | | | | | | | | | | | | | | | |
| CHIP-AE[70] | Participant | 5-point scale | 107 | Y | N | - | - | “The observed relationships between increased health care utilization and impairment in health status support the construct validity of the CHIP-AE.   Comparison of CKD pts results with controls showed statistically significant differences in risk, resilience subdomains and overall satisfaction with Health, Acute Major Disorders, and Long-Term Surgical Disorders.” | - | - | “The comparison to a second, contemporaneously collected CHIP-AE public school sample, showing consistent statistically significant differences in similar domains (Overall Satisfaction with Health, Risks subdomains, and Resilience subdomains).  Clinically significant differences observed, such as less physical activity in CKD patients compared to control subjects.” | - | “Comparison of Health Status of CRI, Dialysis, and Transplant Patients:  Statistically significant differences in physical discomfort, limitations in activities, and physical activity among the three patient groups.  Stepwise increase in physical activity indicating consistent differences among groups.” | - | - | |
| Author-initiated global assessment of change for nephrotic syndrome and overall health[146] | Participant | At the event (post baseline assessment) and month 12 visits, patients were asked “How is your nephrotic syndrome compared to your last study visit?,” and “How is your overall health compared to your last study visit?” each with possible responses of “Better, about the same or Worse.” | 2 | N | Y | - | - | - | - | - | - | - | - | - | “The minimally important difference of 3 points in PROMIS Pediatric measures has been established for children with nephrotic syndrome and other chronic health conditions through research involving patients, caregivers, and physicians. This benchmark indicates that PROMIS Mobility, Fatigue, Pain Interference, and Anxiety measures effectively detected changes exceeding 3 points, correlating with patients’ global assessments of their nephrotic syndrome and overall health. The effect sizes were notably stronger for changes in overall health compared to nephrotic syndrome-specific changes, which aligns with the non-disease-specific nature of PROMIS domains, making them more responsive to general health alterations.  An important finding from the analyses is that, except for rare instances, PROMIS domain score changes did not differentiate between patients achieving proteinuria remission and those with ongoing active disease during follow-up.” | |
| PRO-KID[92, 122] | Participant | 5-point likert scale for the dimensions of frequency and impact. | 14 | Y | Y | **Jawa 2022:**  Looked at face validity through semi-structured interview | - | **Matsuda-Abedini 2024:** “Pearson correlations between PRO-Kid and PedsQL scores were strong: R −0.78 (95% CI, −0.85 to −0.70) for the frequency score and R −0.69 (95% CI, −0.78 to −0.56) for the impact score, reflecting poorer quality-of-life association with higher symptoms burden.” | - | **Matsuda-Abedini 2024:** “CFA models with one latent factor demonstrated good fit for both frequency (CFI = 0.993, RMSEA = 0.032, SRMR = 0.105, with residual correlation added between "tired" and "sleep," r = 0.48) and impact instruments (CFI = 0.987, RMSEA = 0.045, SRMR = 0.117). All items were strongly associated with latent factors (standardized loadings ≥ 0.40), confirming uni-dimensionality with no ill-fitting items.” | - | - | **Matsuda-Abedini 2024:** “Cα was high for both the PRO-Kid frequency and impact, 0.83 (95% confidence interval [CI], 0.78 to 0.88) and 0.84 (95% CI, 0.79 to 0.88), respectively, showing strong internal consistency” | - | - | |
| **Behavior/mental health** | | | | | | | | | | | | | | | | |
| CAMM (child and adolescent mindfulness measure)[88] | Participant | 4-point Likert scale | 10 | Y | N | - | - | - | - | - | - | - | “Cronbach's alpha was 0.81, and was comparable to the previously reported Cronbach's alpha of 0.80” | - | - | |
| CBCL[81] | Participant or proxy | 3-point Likert scale | 113 | Y | N | - | - | - | - | - | - | - | “Cronbach’s alpha was 0.86 for parent-reports and 0.83 for self-reports” | - | - | |
| YSR[81] | Participant or proxy | 3-point Likert scale and open-ended questions | 113+ | Y | N |  |  |  |  |  |  |  | “Cronbach’s alpha was 0.86 for parent-reports and 0.83 for self-reports” |  |  | |
| **Other** | | | | | | | | | | | | | | | | |
| Study-specific semi-structured interview based on PROMIS domains [67] | Participant | Open-ended questions | - | N | N | “The findings suggest that children with Crohn’s disease and chronic kidney disease share similar breadth and types of lived experiences with children from the PROMIS development samples who do not have chronic illness. This indicates that the presence of chronic illness does not affect the content validity of the universally experienced health domains examined. Specifically, the content of four PROMIS measures—pediatric pain interference and fatigue measures for children with Crohn’s disease, and pediatric fatigue, sleep disturbance, and sleep-related impairment measures for children with chronic kidney disease adequately captures the diverse manifestations reported by participants in this study.” | - | - | - | - | - | - | - | - | - | |
| Study-developed PREM on hospital and clinical staff experience[20] | Participant or proxy | 5-point Likert scale (from 1—completely agree to 5—completely disagree) | 12 | N | Y | - | - | - | - | - | - | - | “Proxy-reported PREM questionnaires showed high internal consistency (Cronbach alpha 0.856), which was similarly lower in the 2–4-year-old group (0.554) but higher than 0.7 in all the remaining groups. Cronbach’s alpha for children reported in PREMs questionnaires was 0.911.” | - | - | |
| Study-developed self-care measurement scale (in Persian)[101] | Participant | Yes/no response format | 60 | N | Y | - | - | - | - | - | - | - | “Cronbach’s alpha coefficients were diet adjustment (0.94), stress prevention (0.86), food safety (0.79), sports activities regulation (0.83), and habits (0.77). The total score of the universal self-care needs Cronbach’s alpha was 0.88.” | - | - | |
| PedsQL Oral health scale (Brazilian version)[149]  (used in parallel with PedsQL 4.0) | Participant or proxy | 5-point Likert scale | 5 | Y | N | - | - | - | - | - | - | “Intra-examiner reliability was determined by the re-evaluation of 20 children and adolescents within a two-week interval (10% of the sample) and by means of Kappa values: 0.9 for decayed, missing, and filled teeth index; 0.8 for plaque index and gingival index system and 1.0 for developmental enamel defect index.” | - | - | - | |
| Pediatric Renal Caregiver Burden Scale (PR-CBS)[61] | Proxy | 3-point Likert scale | 51 | Y | Y | Paper stated that the authors conducted this, but no further data was given | - | - | - | - | - | “Researchers conducted test-retest reliability by administrating the tool to the same subjects under similar conditions on one or more occasions. Answers from repeat testing were compared.” | - | - | - | |
| Pediatric PROMIS measures of physical health (Mobility, Fatigue, Pain Interference), mental health (Depressive Symptoms and Anxiety) and social health (Peer Relationships)[146] | Participant or proxy | 5- point Likert scale and 1 10-point rating of pain intensity | 7 | Y | N | - | - | “Correlations between changes in PROMIS and PedsQL scores across similar domains were generally strong. For instance, PROMIS Mobility correlated with PedsQL Physical Functioning (r = 0.60), and PROMIS Anxiety showed a negative correlation with PedsQL Anxiety (r = −0.68), indicating higher PROMIS Anxiety was associated with worse emotional functioning in PedsQL, as expected. However, PROMIS Peer Relationships did not exhibit a significant correlation with PedsQL Social Functioning (r = 0.11).” | - | - | - | - | - | - | “The minimally important difference of 3 points in PROMIS Pediatric measures has been established for children with nephrotic syndrome and other chronic health conditions through research involving patients, caregivers, and physicians. This benchmark indicates that PROMIS Mobility, Fatigue, Pain Interference, and Anxiety measures effectively detected changes exceeding 3 points, correlating with patients’ global assessments of their nephrotic syndrome and overall health. The effect sizes were notably stronger for changes in overall health compared to nephrotic syndrome-specific changes, which aligns with the non-disease-specific nature of PROMIS domains, making them more responsive to general health alterations.  An important finding from the analyses is that, except for rare instances, PROMIS domain score changes did not differentiate between patients achieving proteinuria remission and those with ongoing active disease during follow-up.” | |
| PSAS (patient self-advocacy scale)[88] | Participant | 5-point Likert scale | 12 | Y | N | - | - | - | - | - | - | “Overall Cronbach's alpha was 0.50, and internal consistencies of the subscales were 0.68 for illness education, 0.22 for assertiveness, and 0.67 for mindful nonadherence.” | - | - | - | |
| STARx (The Self-Management and Transition to Adulthood with Rx = Treatment (STARx) Questionnaire) [88] | Participant | 5-point Likert scale | 13 | Y | N | - | - | - | - | - | - | “The overall internal consistency of the STARx was 0.77, and the subscales were for disease knowledge (0.49), self-management (0.72), and for provider communication (0.41).” | - | - | - | |
| Study-specific questionnaire covering treatment, experience with healthcare professionals, and stress[96] | Proxy | Open-ended interview questions | 13 | N | Y | - | - | The content validity of the questionnaire was tested by a team of three consultants in pediatric nephrology. Face validity was evaluated by giving the questionnaire to five parents. No further results given. | - | - | - | - | - | - | - | |

**References**

1.	Abbasi A, Valizadeh M, Fahimi D, Moghtaderi M, Bazargani B, Mojtahedi SY, et al. (2022) Heath-Related Quality of Life in Iranian Children with Nephrotic Syndrome. Iranian Journal of Pediatrics 32(2) (no pagination):<https://doi.org/https://doi.org/10.5812/ijp-118426>

2.	Abrao RO, Lopes M, Silva GJS, Ferraro AA, Koch VH (2021) Study of the association between generic and disease-specific quality of life and behavior problems in pediatric patients with chronic kidney disease stage 3 or higher and the quality of life and mental health of their primary caregivers. Pediatric Nephrology 36:3201-10. <https://doi.org/https://doi.org/10.1007/s00467-021-04986-0>

3.	Abreu IS, Cabral Kourrouski MF, de Souza Serio dos Santos DM, Bullinger M, Nascimento LC, de Lima RAG, et al. (2014) Children and adolescents on hemodialysis: attributes associated with quality of life. Revista da Escola de Enfermagem da USP 48:602-9. <https://doi.org/https://doi.org/10.1590/S0080-623420140000400005>

4.	Agrawal S, Krishnamurthy S, Naik BN (2017) Assessment of quality of life in children with nephrotic syndrome at a teaching hospital in South India. Saudi J Kidney Dis Transpl 28:593-8. <https://doi.org/https://doi.org/10.4103/1319-2442.206452>

5.	Ahmed A, Cote A, Lui S, Blydt-Hansen TD (2022) Height-adjusted lean body mass and its associations with physical activity and kidney function in pediatric kidney transplantation. Pediatric Transplantation 26(1) (no pagination):<https://doi.org/https://doi.org/10.1111/petr.14128>

6.	Al-Uzri A, Matheson M, Gipson DS, Mendley SR, Hooper SR, Yadin O, et al. (2013) The impact of short stature on health-related quality of life in children with chronic kidney disease. Journal of Pediatrics 163:736-41.e1. <https://doi.org/https://doi.org/10.1016/j.jpeds.2013.03.016>

7.	Albatati S, Alnasser Y, Alomar O, Alsharidi T, Almousa H, Almezel S, et al. (2024) Social determinants of health and quality of life in children with chronic kidney disease: insights from Saudi Arabia. BMC Nephrology 25:272. <https://doi.org/https://doi.org/10.1186/s12882-024-03710-y>

8.	Alhamed AA, Toly VB, Hooper SR, Dell KM (2022) The link between executive function, socio-emotional functioning and health-related quality of life in children and adolescents with mild to moderate chronic kidney disease. Child: Care, Health and Development 48:455-64. <https://doi.org/https://doi.org/10.1111/cch.12946>

9.	Alhusaini OA, Wayyani LA, Dafterdar HE, Gamlo MM, Alkhayat ZA, Alghamdi AS, et al. (2019) Comparison of quality of life in children undergoing peritoneal dialysis versus hemodialysis. Saudi Medical Journal 40:840-3. <https://doi.org/https://doi.org/10.15537/smj.2019.8.12747>

10.	Almardini R, Taybeh EO, Alsous MM, Hawwa AF, McKeever K, Horne R, et al. (2019) A multiple methods approach to determine adherence with prescribed mycophenolate in children with kidney transplant. British Journal of Clinical Pharmacology 85(7):1434-42. <https://doi.org/https://doi.org/10.1111/bcp.13911>

11.	Al Qaisy U, Ali SH, Ali IH (2019) Assessment of health-related quality of life in children with nephrotic syndrome in Iraq--A comparative study. Indian Journal of Public Health 10:2657-62. <https://doi.org/https://doi.org/10.5958/0976-5506.2019.03268.6>

12.	Aman NF, Fitzpatrick J, de Verteuil I, Vasilevska-Ristovska J, Banh THM, Korczak DJ, et al. (2022) Family functioning and quality of life among children with nephrotic syndrome during the first pandemic wave. Pediatric Nephrology <https://doi.org/https://doi.org/10.1007/s00467-022-05809-6>

13.	Amin R, Sharma N, Al-Mokali K, Sayal P, Al-Saleh S, Narang I, et al. (2015) Sleep-disordered breathing in children with chronic kidney disease. Pediatric Nephrology 30:2135-43. <https://doi.org/https://doi.org/10.1007/s00467-015-3155-x>

14.	Amr M, Bakr A, El Gilany AH, Hammad A, El-Refaey A, El-Mougy A (2009) Multi-method assessment of behavior adjustment in children with chronic kidney disease. Pediatric Nephrology 24:341-7. <https://doi.org/https://doi.org/10.1007/s00467-008-1012-x>

15.	Anthony SJ, Hebert D, Todd L, Korus M, Langlois V, Pool R, et al. (2010) Child and parental perspectives of multidimensional quality of life outcomes after kidney transplantation. Pediatric Transplantation 14:249-56. <https://doi.org/https://doi.org/10.1111/j.1399-3046.2009.01214.x>

16.	Aparicio Lopez C, Fernandez Escribano A, Garrido Cantanero G, Luque A, Izquierdo Garcia E (2010) [Perceived quality of life in children with chronic renal disease and in their parents]. Nefrologia 30:103-9. <https://doi.org/https://doi.org/10.3265/Nefrologia.pre2009.Dic.5683>

17.	Aparicio Lopez C, Fernandez Escribano A, Garrido Cantanero G, Luque de Pablos A, Izquierdo Garcia E (2010) [Design of a quality of life questionnaire in Spanish for children with chronic kidney disease]. Nefrologia 30:168-76. <https://doi.org/https://doi.org/10.3265/Nefrologia.pre2009.Dic.5684>

18.	Aparicio-Lopez C, Fernandez-Escribano A, Garrido-Cantanero G, Luque-de Pablos A, Izquierdo-Garcia E (2013) The influence of clinical situation on health-related quality of life in paediatric chronic kidney disease patients. Nefrologia 33:61-9. <https://doi.org/https://doi.org/10.3265/Nefrologia.pre2012.Jul.11484>

19.	Ayestaran FW, Schneider MF, Kaskel FJ, Srivaths PR, Seo-Mayer PW, Moxey-Mims M, et al. (2016) Perceived appetite and clinical outcomes in children with chronic kidney disease. Pediatric Nephrology 31:1121-7. <https://doi.org/https://doi.org/10.1007/s00467-016-3321-9>

20.	Ažukaitis K, Puronaitė R, Vaitkevičienė GE, Trinkūnas J, Jankauskienė A, Jankauskienė D (2023) Integrated Collection of Patient-Reported Outcomes and Experiences in Children with Kidney and Hematological Diseases: A Pilot Study. Children (Basel) 10:<https://doi.org/https://doi.org/10.3390/children10071245>

21.	Baek HS, Kang HG, Choi HJ, Cheong HI, Ha IS, Han KH, et al. (2017) Health-related quality of life of children with pre-dialysis chronic kidney disease. Pediatr Nephrol 32:2097-105. <https://doi.org/10.1007/s00467-017-3721-5>

22.	Baek HS, Park KS, Ha IS, Kang HG, Cheong HI, Park YS, et al. (2018) Impact of end-stage renal disease in children on their parents. Nephrology 23:764-70. <https://doi.org/https://doi.org/10.1111/nep.13083>

23.	Bakar KA, Muhamad NA, Sarpin MA, Shaharudin S, Sidhu S, Yap SL, et al. (2023) Health-related quality of life amongst children with chronic kidney disease in Malaysia: performance of the Bahasa Melayu version of the PedsQL 3.0 ESRD Module: (PedsQL 3.0 VerBATIM: version in Bahasa Melayu. Translated for use in Malaysia). Pediatric Nephrology 38:1897-905. <https://doi.org/https://doi.org/10.1007/s00467-022-05774-0>

24.	Bamford J, Wirz L (2016) Piloting Psychology Annual Reviews as a Method of Measuring Psychological Distress and Quality of Life in Paediatric Renal Transplant Patients. BioMed Research International 2016:1685362. <https://doi.org/https://doi.org/10.1155/2016/1685362>

25.	Bauer A, Blanchette E, Taylor Zimmerman C, Wightman A (2021) Caregiver burden in pediatric dialysis: application of the Paediatric Renal Caregiver Burden Scale. Pediatric Nephrology 36:3945-51. <https://doi.org/10.1007/s00467-021-05149-x>

26.	Berney-Martinet S, Key F, Bell L, Lepine S, Clermont MJ, Fombonne E (2009) Psychological profile of adolescents with a kidney transplant. Pediatric Transplantation 13(6):701-10. <https://doi.org/https://doi.org/10.1111/j.1399-3046.2008.01053.x>

27.	Brunelli G, Nogueira PC, Pestana JO, Diniz DH (2012) Quality of life in children on dialysis. Int Urol Nephrol 44:1515-9. <https://doi.org/https://doi.org/10.1007/s11255-011-0047-2>

28.	Buyan N, Turkmen MA, Bilge I, Baskin E, Haberal M, Bilginer Y, et al. (2010) Quality of life in children with chronic kidney disease (with child and parent assessments). Pediatric Nephrology 25(8):1487-96. <https://doi.org/https://doi.org/10.1007/s00467-010-1486-1>

29.	Buyukkaragoz B, Buyan N, Dogrucan N, Celik B (2016) Health-related quality of life of pediatric renal transplant recipientsand their parents: the role of associated factors and clinical counseling. Turkish Journal of Medical Sciences 46:1481-5. <https://doi.org/https://doi.org/10.3906/sag-1505-18>

30.	Carlson J, Gerson AC, Matheson MB, Manne S, Warady BA, Hooper SR, et al. (2020) A longitudinal analysis of the effect of anemia on health-related quality of life in children with mild-to-moderate chronic kidney disease. Pediatric Nephrology 35:1659-67. <https://doi.org/https://doi.org/10.1007/s00467-020-04569-5>

31.	Carlson J, Gerson AC, Matheson MB, Manne S, Lande M, Harshman L, et al. (2023) Longitudinal changes of health-related quality of life in childhood chronic kidney disease. Pediatr Nephrol 38:4127-36. <https://doi.org/https://doi.org/10.1007/s00467-023-06069-8>

32.	Cavusoglu I, Tarakci D, Zenginler Yazgan Y, Yavuz S (2023) Comparison of effectiveness of Nintendo Wii-based exergaming and home-based fun video exercises in pediatric patients with chronic kidney disease. International Journal of Rehabilitation Research 46:26-34. <https://doi.org/https://doi.org/10.1097/MRR.0000000000000554>

33.	Chan EYH, Liu MS, Or PC, Ma ALT (2022) Outcomes and perception of cloud-based remote patient monitoring in children receiving automated peritoneal dialysis: a prospective study. Pediatric nephrology 30:<https://doi.org/https://doi.org/10.1007/s00467-022-05828-3>

34.	Chan M, Young S, Hanna M (2024) Improving Transition of Care for Pediatric Patients With Chronic Kidney Disease: A Pilot Project. Cureus 16:e63367. <https://doi.org/https://doi.org/10.7759/cureus.63367>

35.	Cheung V, Wertenteil S, Sasson S, Vento S, Kothare S, Trachtman H (2015) Restless Legs Syndrome in Pediatric Patients With Nephrotic Syndrome. 1:2333794x15585994. <https://doi.org/https://doi.org/10.1177/2333794X15585994>

36.	Chien-Ning HSU, Hsiang-Wen LIN, Pickard AS, You-Lin T, Hsu C-N, Lin H-W, et al. (2018) EQ-5D-Y for the assessment of health-related quality of life among Taiwanese youth with mild-to-moderate chronic kidney disease. International Journal for Quality in Health Care 30:298-305. <https://doi.org/https://doi.org/10.1093/intqhc/mzy011>

37.	Clark S, Denburg M, Furth S (2016) Physical activity and screen time in adolescents in the chronic kidney disease in children (CKiD) cohort. Pediatric Nephrology 31:801-8. <https://doi.org/https://doi.org/10.1007/s00467-015-3287-z>

38.	Clavé S, Tsimaratos M, Boucekine M, Ranchin B, Salomon R, Dunand O, et al. (2019) Quality of life in adolescents with chronic kidney disease who initiate haemodialysis treatment. BMC Nephrology 20:N.PAG-N.PAG. <https://doi.org/https://doi.org/10.1186/s12882-019-1365-3>

39.	Coleman JE, Watson AR, Chowdhury S, Thurlby D, Wardell J, Coleman JE, et al. (2002) Comparison of two micronutrient supplements in children with chronic renal failure. Journal of Renal Nutrition 12:244-7. <https://doi.org/https://doi.org/10.1053/jren.2002.35317>

40.	Conde García P, Coyo Montero M, Tosquella Ambiás R (2010) Compliance with treatment in paediatric renal transplant. Revista de la Sociedad Española de Enfermería Nefrológica 13:173-9.

41.	Craven S, Brumbach BH, Richardson KL (2023) Patient- and caregiver-reported factors associated with school absenteeism in children with chronic kidney disease. Pediatric Nephrology 38:1591-8. <https://doi.org/https://doi.org/10.1007/s00467-022-05780-2>

42.	Davis I, Greenbaum L, Gipson D, Wu L, Sinha R, Matsuda-Abedini M, et al. (2012) Prevalence of sleep disturbances in children and adolescents with chronic kidney disease. Pediatric Nephrology 27:451-9. <https://doi.org/https://doi.org/10.1007/s00467-011-2010-y>

43.	De Bruyne E, Eloot S, Vande Walle J, Raes A, Van Biesen W, Goubert L, et al. (2022) Validity and reliability of the Dutch version of the PedsQL™ 3.0 End Stage Renal Disease Module in children with chronic kidney disease in Belgium. Pediatric Nephrology 37:1087-96. <https://doi.org/https://doi.org/10.1007/s00467-021-05224-3>

44.	de Pádua Paz I, Konstantyner T, de Castro Cintra Sesso R, de Xavier Pinto CC, de Camargo MFC, Nogueira PCK (2021) Access to treatment for chronic kidney disease by children and adolescents in Brazil. Pediatric Nephrology 36:2827-35. <https://doi.org/https://doi.org/10.1007/s00467-021-05009-8>

45.	Demir MHB, Taner S, Demir HB, Tasdemir AI, Keskinoglu A, Bulut IK, et al. (2023) Quality of Life and Psychological Well-Being in Children and Adolescents After Renal Transplantation. Transplantation Proceedings <https://doi.org/https://doi.org/10.1016/j.transproceed.2023.04.022>

46.	DeWalt DA, Gross HE, Gipson DS, Selewski DT, DeWitt EM, Dampier CD, et al. (2015) PROMIS( R) pediatric self-report scales distinguish subgroups of children within and across six common pediatric chronic health conditions. Quality of Life Research 24:2195-208. <https://doi.org/https://doi.org/10.1007/s11136-015-0953-3>

47.	Díaz-González de Ferris ME, Pierce CB, Gipson DS, Furth SL, Warady BA, Hooper SR (2021) Health-related quality of life in children with chronic kidney disease is affected by the number of medications. Pediatric Nephrology 36:1307-10. <https://doi.org/https://doi.org/10.1007/s00467-021-04919-x>

48.	Didsbury M, van Zwieten A, Chen K, James LJ, Francis A, Kim S, et al. (2019) The association between socioeconomic disadvantage and parent-rated health in children and adolescents with chronic kidney disease-the Kids with CKD (KCAD) study. Pediatr Nephrol 34:1237-45. <https://doi.org/10.1007/s00467-019-04209-7>

49.	Dobbels F, Decorte A, Roskams A, Van Damme-Lombaerts R (2010) Health-related quality of life, treatment adherence, symptom experience and depression in adolescent renal transplant patients. Pediatric Transplantation 14:216-23. <https://doi.org/https://doi.org/10.1111/j.1399-3046.2009.01197.x>

50.	Dodson JL, Cohn SE, Cox C, Hmiel PS, Wood E, Mattoo TK, et al. (2009) Urinary incontinence in the CKiD cohort and health related quality of life. Journal of Urology 182:2007-14. <https://doi.org/https://doi.org/10.1016/j.juro.2009.06.012>

51.	Dodson JL, Diener-West M, Gerson AC, Kaskel FJ, Furth SL (2007) An assessment of health related quality of life using the child health and illness profile-adolescent edition in adolescents with chronic kidney disease due to underlying urological disorders. Journal of Urology 178:660-5; discussion 5. <https://doi.org/https://doi.org/10.1016/j.juro.2007.04.008>

52.	Dodson JL, Gerson AC, Hsiao CJ, Kaskel FJ, Weiss RA, Furth SL (2008) Parent perspectives of health related quality of life in adolescents with chronic kidney disease due to underlying urological disorders: an assessment using the Child Health Questionnaire-Parent Form 50. Journal of Urology 180:1700-4; discussion 4. <https://doi.org/https://doi.org/10.1016/j.juro.2008.03.117>

53.	Dotis J, Pavlaki A, Printza N, Stabouli S, Antoniou S, Gkogka C, et al. (2016) Quality of life in children with chronic kidney disease. Pediatric Nephrology 31:2309-16. <https://doi.org/https://doi.org/10.1007/s00467-016-3457-7>

54.	Duverger P (2013) [Quality of life after kidney transplantation in adolescence]. Bulletin de l Academie Nationale de Medecine 197:1583-97; discussion 97-8.

55.	Eid R, Fathy AA, Hamdy N (2020) Health-related quality of life in Egyptian children with nephrotic syndrome. Quality of Life Research: An International Journal of Quality of Life Aspects of Treatment, Care & Rehabilitation 29:2185-96. <https://doi.org/https://doi.org/10.1007/s11136-020-02438-0>

56.	Eijsermans RM, Creemers DG, Helders PJ, Schroder CH (2004) Motor performance, exercise tolerance, and health-related quality of life in children on dialysis. Pediatric Nephrology 19:1262-6. <https://doi.org/https://doi.org/10.1007/s00467-004-1583-0>

57.	El Shafei AM, Soliman Hegazy I, Fadel FI, Nagy EM (2018) Assessment of Quality of Life among Children with End-Stage Renal Disease: A Cross-Sectional Study. Journal of Environmental & Public Health 1-6. <https://doi.org/https://doi.org/10.1155/2018/8565498>

58.	El-baroudy N, El Falaki M, Hagras A, Galal R, Azmy R, El-sayed B, et al. (2020) Sleep disorders in children and adolescents on regular hemodialysis. European Journal of Pediatrics 179:1139-46. <https://doi.org/https://doi.org/10.1007/s00431-020-03611-w>

59.	El-Gamasy MA, Eldeeb MM (2017) Assessment of physical and psychosocial status of children with ESRD under regular hemodialysis, a single centre experience. International Journal of Pediatrics and Adolescent Medicine 4(2):81-6. <https://doi.org/https://doi.org/10.1016/j.ijpam.2017.01.001>

60.	El-Refaey AM, Elsayed RM, Sarhan A, Bakr A, Hammad A, Elmougy A, et al. (2013) Sleep quality assessment using polysomnography in children on regular hemodialysis. Saudi J Kidney Dis Transpl 24:714-8. <https://doi.org/https://doi.org/10.4103/1319-2442.113862>

61.	Elabbassy A, Atia M, Alam F (2015) The effectiveness of Practical Guides on Burden's Coping Strategies among Caregiver of Children Undergoing Hemodialysis. International Journal of Nursing 2:<https://doi.org/https://doi.org/10.15640/ijn.v2n2a13>

62.	Esmaeeli MR, Erfani Sayar R, Saghebi A, Rahmani S, Elmi S, Rabbani Javadi A (2014) Screening for depression in hospitalized pediatric patients. Iranian Journal of Child Neurology 8(1):47-51.

63.	Fadrowski J, Cole SR, Hwang W, Fiorenza J, Weiss RA, Gerson A, et al. (2006) Changes in physical and psychosocial functioning among adolescents with chronic kidney disease. Pediatric Nephrology 21:394-9. <https://doi.org/https://doi.org/10.1007/s00467-005-2122-3>

64.	Falger J, Landolt MA, Latal B, Rüth EM, Neuhaus TJ, Laube GF (2008) Outcome after renal transplantation. Part II: quality of life and psychosocial adjustment. Pediatric Nephrology 23:1347-54. <https://doi.org/https://doi.org/10.1007/s00467-008-0798-x>

65.	Fayer Rotella AA, do Nascimento RA, de Camargo MFC, Koch Nogueira PC (2020) Emotional repercussions and quality of life in children and adolescents undergoing hemodialysis or after kidney transplantation. [Portuguese]. Revista Paulista de Pediatria 38 (no pagination):<https://doi.org/https://doi.org/10.1590/1984-0462/2020/38/2018221>

66.	Fenton N, Ferris M, Ko Z, Javalkar K, Hooper SR (2015) The relationship of health care transition readiness to disease-related characteristics, psychosocial factors, and health care outcomes: preliminary findings in adolescents with chronic kidney disease. Journal of Pediatric Rehabilitation Medicine 8:13-22. <https://doi.org/https://doi.org/10.3233/PRM-150314>

67.	Forrest CB, Forrest KD, Clegg JL, de la Motte A, Amaral S, Grossman AB, et al. (2020) Establishing the content validity of PROMIS Pediatric pain interference, fatigue, sleep disturbance, and sleep-related impairment measures in children with chronic kidney disease and Crohn's disease. Journal of Patientreported Outcomes 4:11. <https://doi.org/https://doi.org/10.1186/s41687-020-0178-2>

68.	Francis A, Didsbury MS, van Zwieten A, Chen K, James LJ, Kim S, et al. (2019) Quality of life of children and adolescents with chronic kidney disease: a cross-sectional study. Arch Dis Child 104:134-40. <https://doi.org/10.1136/archdischild-2018-314934>

69.	Gerson A, Hwang W, Fiorenza J, Barth K, Kaskel F, Weiss L, et al. (2004) Anemia and health-related quality of life in adolescents with chronic kidney disease. American Journal of Kidney Diseases 44:1017-23. <https://doi.org/https://doi.org/10.1053/j.ajkd.2004.08.024>

70.	Gerson AC, Riley A, Fivush BA, Pham N, Fiorenza J, Robertson J, et al. (2005) Assessing health status and health care utilization in adolescents with chronic kidney disease. J Am Soc Nephrol 16:1427-32. <https://doi.org/https://doi.org/10.1681/asn.2004040258>

71.	Gerson AC, Wentz A, Abraham AG, Mendley SR, Hooper SR, Butler RW, et al. (2010) Health-related quality of life of children with mild to moderate chronic kidney disease. Pediatrics 125:e349-57. <https://doi.org/10.1542/peds.2009-0085>

72.	Gipson DS, Selewski DT, Massengill SF, Wickman L, Messer KL, Herreshoff E, et al. (2013) Gaining the PROMIS perspective from children with nephrotic syndrome: A Midwest pediatric nephrology consortium study. Health and Quality of Life Outcomes 11(1) (no pagination):<https://doi.org/https://doi.org/10.1186/1477-7525-11-30>

73.	Goldstein SL, Graham N, Burwinkle T, Warady B, Farrah R, Varni JW (2006) Health-related quality of life in pediatric patients with ESRD. Pediatric Nephrology 21:846-50. <https://doi.org/https://doi.org/10.1007/s00467-006-0081-y>

74.	Goldstein SL, Graham N, Warady BA, Seikaly M, McDonald R, Burwinkle TM, et al. (2008) Measuring health-related quality of life in children with ESRD: performance of the generic and ESRD-specific instrument of the Pediatric Quality of Life Inventory (PedsQL). American Journal of Kidney Diseases 51:285-97. <https://doi.org/https://doi.org/10.1053/j.ajkd.2007.09.021>

75.	Goldstein SL, Rosburg NM, Warady BA, Seikaly M, McDonald R, Limbers C, et al. (2009) Pediatric end stage renal disease health-related quality of life differs by modality: a PedsQL ESRD analysis. Pediatric Nephrology 24:1553-60. <https://doi.org/https://doi.org/10.1007/s00467-009-1174-1>

76.	Gomes C, Oliveira L, Ferreira R, Simao C (2017) Sleep disturbance in pediatric patients on automated peritoneal dialysis. Sleep Medicine 32:87-91. <https://doi.org/https://doi.org/10.1016/j.sleep.2016.12.010>

77.	Guha C, Khalid R, van Zwieten A, Francis A, Hawley CM, Jaure A, et al. (2023) Baseline characteristics of participants in the NAVKIDS<sup>2</sup> trial: a patient navigator program in children with chronic kidney disease. Pediatric Nephrology 38:1577-90. <https://doi.org/https://doi.org/10.1007/s00467-022-05772-2>

78.	Guha C, van Zwieten A, Khalid R, Kim S, Walker A, Francis A, et al. (2023) Longitudinal assessment of the health-related quality of life of children and adolescents with chronic kidney disease. Kidney Int 103:357-64. <https://doi.org/10.1016/j.kint.2022.09.026>

79.	Guilfoyle SM, Goebel JW, Pai ALH (2011) Efficacy and flexibility impact perceived adherence barriers in pediatric kidney post-transplantation. Families, Systems & Health: The Journal of Collaborative Family HealthCare 29:44-54. <https://doi.org/https://doi.org/10.1037/a0023024>

80.	Guzmán-Carrillo KY, González-Betanzos F, Rivera-Heredia ME, Montes Delgado R, Salazar Garza ML, Aguirre Martínez JI (2022) Intervención cognitivo conductual para promover la adhesión al tratamiento médico, recursos psicológicos y calidad de vida en pacientes pediátricos con hemodiálisis en México. [Cognitive behavioral intervention to promote adherence to medical treatment, psychological resources, and quality of life in pediatric hemodialysis patients in Mexico.]. Revista de Psicología Clínica con Niños y Adolescentes 9:26-31. <https://doi.org/https://doi.org/10.21134/rpcna.2022.09.2.3>

81.	Haavisto A, Korkman M, Sintonen H, Holmberg C, Jalanko H, Lipsanen J, et al. (2013) Risk factors for impaired quality of life and psychosocial adjustment after pediatric heart, kidney, and liver transplantation. Pediatric Transplantation 17(3):256-65. <https://doi.org/https://doi.org/10.1111/petr.12054>

82.	Hajj E, Awouters M, Mosca M, Flammier S, Rachedi S, Bacchetta J, et al. (2025) Health-related quality of life in paediatric patients on peritoneal dialysis: Data from a tertiary centre. Perit Dial Int 45:121-3. <https://doi.org/https://doi.org/10.1177/08968608241241177>

83.	Hamedi-Kalajahi F, Imani H, Mojtahedi S, Shabbidar S (2022) Effect of L-Carnitine Supplementation on Inflammatory Markers and Serum Glucose in Hemodialysis Children: A Randomized, Placebo-Controlled Clinical Trial. Journal of Renal Nutrition 32:144-51. <https://doi.org/https://doi.org/10.1053/j.jrn.2021.03.009>

84.	Harmer M, Wootton S, Gilbert R, Anderson C (2019) Association of nutritional status and health-related quality of life in children with chronic kidney disease. Quality of Life Research: An International Journal of Quality of Life Aspects of Treatment, Care & Rehabilitation 28:1565-73. <https://doi.org/https://doi.org/10.1007/s11136-019-02104-0>

85.	Heath J, MacKinlay D, Watson A, Hames A, Wirz L, Scott S, et al. (2011) Self-reported quality of life in children and young people with chronic kidney disease. Pediatric Nephrology 26:767-73. <https://doi.org/https://doi.org/10.1007/s00467-011-1784-2>

86.	Heath J, Norman P, Christian M, Watson A (2017) Measurement of quality of life and attitudes towards illness in children and young people with chronic kidney disease. Quality of Life Research 26:2409-19. <https://doi.org/https://doi.org/10.1007/s11136-017-1605-6>

87.	Holroyd J, Guthrie D (1986) Family stress with chronic childhood illness: Cystic fibrosis, neuromuscular disease, and renal disease. Journal of Clinical Psychology 42:552-61. <https://doi.org/https://doi.org/10.1002/1097-4679%28198607%2942:4%3C552::AID-JCLP2270420403%3E3.0.CO;2-8>

88.	Huang Y, Rak E, Faldowski RA, Nazareth M, Ryan J, Javalkar K, et al. (2022) The self-management and transition manual "ALL YOU NEED IS LOVE" for adolescents with chronic kidney disease. Journal of pediatric nursing 14:<https://doi.org/https://doi.org/10.1016/j.pedn.2022.10.006>

89.	Hsu CN, Tain YL, Lu PC, Lin HW (2023) Comparisons of EQ-5D-Y and PedsQL in pediatric patients with mild-to-moderate chronic kidney disease in longitudinal analyses. Health Qual Life Outcomes 21:117. <https://doi.org/https://doi.org/10.1186/s12955-023-02197-9>

90.	Imani PD, Aujo J, Kiguli S, Srivaths P, Brewer ED (2021) Chronic kidney disease impacts health-related quality of life of children in Uganda, East Africa. Pediatric Nephrology 36:323-31. <https://doi.org/https://doi.org/10.1007/s00467-020-04705-1>

91.	Jalali-Farahani S, Shojaei FA, Parvin P, Amiri P (2018) Comparison of health-related quality of life (HRQoL) among healthy, obese and chronically ill Iranian children. BMC Public Health 18:N.PAG-N.PAG. <https://doi.org/https://doi.org/10.1186/s12889-018-6239-2>

92.	Jawa NA, Rapoport A, Widger K, Zappitelli M, Davison SN, Jha S, et al. (2022) Development of a patient-reported outcome measure for the assessment of symptom burden in pediatric chronic kidney disease (PRO-Kid). Pediatric Nephrology 37:1377-86. <https://doi.org/https://doi.org/10.1007/s00467-021-05269-4>

93.	Johnson RJ, Gerson AC, Harshman LA, Matheson MB, Shinnar S, Lande MB, et al. (2020) A longitudinal examination of parent-reported emotional-behavioral functioning of children with mild to moderate chronic kidney disease. Pediatr Nephrol 35:1287-95. <https://doi.org/https://doi.org/10.1007/s00467-020-04511-9>

94.	Kang NR, Ahn YH, Park E, Choi HJ, Kim SH, Cho H, et al. (2019) Mental health and psychosocial adjustment in pediatric chronic kidney disease derived from the KNOW-Ped CKD study. Pediatr Nephrol 34:1753-64. <https://doi.org/https://doi.org/10.1007/s00467-019-04292-w>

95.	Karava V, Goutou S, Dotis J, Kondou A, Charela E, Dadoudi O, et al. (2022) Fatigue and Quality of Life in Children with Chronic Kidney Disease. Children 9:N.PAG-N.PAG. <https://doi.org/https://doi.org/10.3390/children9091414>

96.	Kari JA, Alzahrany M, El-Deek B, Maimani M, El-Desoky S (2014) Social Impact of Dialysis on Children and Their Families. The Indian Journal of Pediatrics 81:1020-6. <https://doi.org/https://doi.org/10.1007/s12098-013-1236-z>

97.	Kasemy ZA, Hathout HM, Omar ZA, Samir MA, Bahbah WA (2020) Effect of Omega-3 supplements on quality of life among children on dialysis: A prospective cohort study. Medicine 99:e22240. <https://doi.org/https://doi.org/10.1097/MD.0000000000022240>

98.	Khalf-Allah SH, Ebrahim H, Badawy A, Sayed H (2024) Effect of muscle stretching and isometric exercises on quality of life in children undergoing regular hemodialysis. Pediatr Nephrol 39:3289-99. <https://doi.org/https://doi.org/10.1007/s00467-024-06398-2>

99.	Khalil AMS, Osman FES (2020) Psychological and Social Status of Children Treated with Renal Dialysis and their Mothers at Tanta City. International Journal of Nursing Education 12:275-84. <https://doi.org/https://doi.org/10.37506/ijone.v12i4.11263>

100.	Khanjari S, Jahanian S, Haghani H (2018) The effect of blended training on the quality of life of children with nephrotic syndrome. J Family Med Prim Care 7:921-6. <https://doi.org/https://doi.org/10.4103/jfmpc.jfmpc_176_18>

101.	Khazaei F, Razaghi N, Vashani HB (2021) Effectiveness of a Support-Training Program based on the Orem’s Self-Care Deficit Theory on the Quality of Life of Children Undergoing Hemodialysis. Journal of Evidence-based Care 11:7-15. <https://doi.org/https://doi.org/10.22038/EBCJ.2021.53217.2405>

102.	Kilicoglu AG, Bahali K, Canpolat N, Bilgic A, Mutlu C, Yalçın Ö, et al. (2016) Impact of end-stage renal disease on psychological status and quality of life. Pediatrics International 58:1316-21. <https://doi.org/https://doi.org/10.1111/ped.13026>

103.	Kilis-Pstrusinska K, Morawska Z (1994) [Perception of family by children and youth with chronic renal failure]. Wiadomosci Lekarskie 47:766-70.

104.	Kilis-Pstrusinska K, Wasilewska A, Medynska A, Balasz-Chmielewska I, Grenda R, Kluska-Jozwiak A, et al. (2013) Psychosocial aspects of children and families of children treated with automated peritoneal dialysis. Pediatric Nephrology 28:2157-67. <https://doi.org/https://doi.org/10.1007/s00467-013-2532-6>

105.	Kilis-Pstrusinska K, Medynska A, Adamczyk P, Balasz-Chmielewska I, Grenda R, Kluska-Jozwiak A, et al. (2014) Disease-related social situation in family of children with chronic kidney disease--parents` assessment. A multicentre study. Annals of Agricultural & Environmental Medicine 21:876-81. <https://doi.org/https://doi.org/10.5604/12321966.1129951>

106.	Knight A, Kogon AJ, Matheson MB, Warady BA, Furth SL, Hooper SR (2017) Cognitive Function in Children with Lupus Nephritis: A Cross-Sectional Comparison with Children with Other Glomerular Chronic Kidney Diseases. Journal of Pediatrics 189:181-8.e1. <https://doi.org/https://doi.org/10.1016/j.jpeds.2017.06.044>

107.	Kogon AJ, Matheson MB, Flynn JT, Gerson AC, Warady BA, Furth SL, et al. (2016) Depressive Symptoms in Children with Chronic Kidney Disease. Journal of Pediatrics 168:164-70.e1. <https://doi.org/https://doi.org/10.1016/j.jpeds.2015.09.040>

108.	Krasnoff JB, Mathias R, Rosenthal P, Painter PL (2006) The Comprehensive Assessment of Physical Fitness in Children Following Kidney and Liver Transplantation. Transplantation 82:211-7. <https://doi.org/https://doi.org/10.1097/01.tp.0000226160.40527.5f>

109.	Krissberg JR, Jones M, Guzman Z, Chen W, Sheehan K, Verghese PS (2025) "I have to pick my battles": a mixed-methods study exploring food insecurity and dietary restrictions in pediatric kidney disease. Pediatr Nephrol 40:473-81. <https://doi.org/https://doi.org/10.1007/s00467-024-06506-2>

110.	Kul M, Cengel Kultur E, Senses Dinc G, Bilginer Y, Uluc S, Baykan H (2013) Quality of life in children and adolescents with chronic kidney disease: a comparative study between different disease stages and treatment modalities. Turkish Journal of Pediatrics 55:493-9.

111.	Kupferman JC, Matheson MB, Lande MB, Flynn JT, Furth S, Warady BA, et al. (2020) Increased history of ischemic stroke and decreased neurocognitive performance in children with chronic kidney disease. Pediatric Nephrology 35:1315-21. <https://doi.org/https://doi.org/10.1007/s00467-020-04503-9>

112.	Lambe T, Frew E, Ives NJ, Woolley RL, Cummins C, Brettell EA, et al. (2018) Mapping the Paediatric Quality of Life Inventory (PedsQL™) Generic Core Scales onto the Child Health Utility Index-9 Dimension (CHU-9D) Score for Economic Evaluation in Children. Pharmacoeconomics 36:451-65. <https://doi.org/https://doi.org/10.1007/s40273-017-0600-7>

113.	Lindeback R, Abdo R, Schnabel L, Le Jambre R, Kennedy SE, Katz T, et al. (2024) Does the Nutritional Intake and Diet Quality of Children With Chronic Kidney Disease Differ From Healthy Controls? A Comprehensive Evaluation. J Ren Nutr 34:283-93. <https://doi.org/https://doi.org/10.1053/j.jrn.2023.12.002>

114.	Lopes M, Ferraro A, Koch VH (2014) Health-related quality of life of children and adolescents with CKD stages 4-5 and their caregivers. Pediatric Nephrology 29(7):1239-47. <https://doi.org/https://doi.org/10.1007/s00467-014-2769-8>

115.	Lopes M, Koch VH, Varni JW (2011) Translation and cultural adaptation of Peds QL TM ESRD to Portuguese. Jornal Brasileiro de Nefrologia 33:448-56.

116.	Lopes MT, Ferraro AA, Koch VH (2015) Reliability of the Brazilian version of the PedsQL - ESDR questionnaire to evaluate quality of life of children and adolescents. Jornal brasileiro de nefrologia : 'orgao oficial de Sociedades Brasileira e Latino-Americana de Nefrologia 37(2):158-65. <https://doi.org/https://doi.org/10.5935/0101-2800.20150026>

117.	Lui S, de Souza A, Sharma A, Fairbairn J, Schreiber R, Armstrong K, et al. (2020) Physical activity and its correlates in a pediatric solid-organ transplant population. Pediatric Transplantation 24:e13745. <https://doi.org/https://doi.org/10.1111/petr.13745>

118.	Lui S, Hind T, Moon E, de Souza A, Broad K, Fairbairn J, et al. (2021) Self-reported physical activity and lack of association with health-related quality of life in a pediatric solid-organ transplant population. Pediatric Transplantation 25(8) (no pagination):<https://doi.org/https://doi.org/10.1111/petr.14093>

119.	Madden SJ, Hastings RP, V'Ant Hoff W (2002) Psychological adjustment in children with end stage renal disease: the impact of maternal stress and coping. Child: Care, Health & Development 28:323-30. <https://doi.org/https://doi.org/10.1046/j.1365-2214.2002.00273.x>

120.	Maikranz JM, Steele RG, Dreyer ML, Stratman AC, Bovaird JA (2007) The relationship of hope and illness-related uncertainty to emotional adjustment and adherence among pediatric renal and liver transplant recipients. Journal of Pediatric Psychology 32:571-81.

121.	Marciano RC, Soares CM, Diniz JS, Lima EM, Silva JM, Canhestro MR, et al. (2011) Behavioral disorders and low quality of life in children and adolescents with chronic kidney disease. Pediatric Nephrology 26:281-90. <https://doi.org/https://dx.doi.org/10.1007/s00467-010-1683-y>

122.	Matsuda-Abedini M, Zappitelli M, Widger K, Rapoport A, Dionne JM, Chanchlani R, et al. (2024) Validation of Patient-Reported Outcome Measure in Pediatric CKD (PRO-Kid). Clin J Am Soc Nephrol 19:851-9. <https://doi.org/10.2215/cjn.0000000000000467>

123.	Mavis AM, Ertl A, Chapman S, Cassidy LD, Lerret SM (2015) Vulnerability and chronic illness management in pediatric kidney and liver transplant recipients. Prog Transplant 25:139-46. <https://doi.org/https://dx.doi.org/10.7182/pit2015551>

124.	McKenna AM, Keating LE, Vigneux A, Stevens S, Williams A, Geary DF (2006) Quality of life in children with chronic kidney disease-patient and caregiver assessments. Nephrol Dial Transplant 21:1899-905.

125.	Medynska A, Zwolinska D, Grenda R, Miklaszewska M, Szczepanska M, Urzykowska A, et al. (2017) Psychosocial aspects of children and families treated with hemodialysis. Hemodial 21:557-65. <https://doi.org/https://dx.doi.org/10.1111/hdi.12526>

126.	Moreira J, Bouissou Morais Soares C, Teixeira A, Simões e Silva A, Kummer A (2015) Anxiety, depression, resilience and quality of life in children and adolescents with pre-dialysis chronic kidney disease. Pediatric Nephrology 30:2153-62. <https://doi.org/10.1007/s00467-015-3159-6>

127.	Neul SK, Minard CG, Currier H, Goldstein SL (2013) Health-related quality of life functioning over a 2-year period in children with end-stage renal disease. Pediatr Nephrol 28:285-93. <https://doi.org/https://dx.doi.org/10.1007/s00467-012-2313-7>

128.	Ng DK, Xu Y, Hogan J, Saland JM, Greenbaum LA, Furth SL, et al. (2020) Timing of patient-reported renal replacement therapy planning discussions by disease severity among children and young adults with chronic kidney disease. Pediatric Nephrology 35:1925-33. <https://doi.org/10.1007/s00467-020-04542-2>

129.	Palupi-Baroto R, Indraswari BW, Mulyadi AWE, Rusdhy SN, Prabowo KN, Hermawan K (2023) Assessing Health-related Quality of Life in Children with Chronic Kidney Disease in Indonesia: A Single Tertiary Hospital Study. Saudi J Kidney Dis Transpl 34:S66-s78. <https://doi.org/10.4103/sjkdt.sjkdt_231_22>

130.	Pardede SO, Rafli A, Gunardi H (2019) Quality of Life in Chronic Kidney disease children using assessment Pediatric Quality Of Life Inventory TM. Saudi J Kidney Dis Transpl 30:812-8. <https://doi.org/https://dx.doi.org/10.4103/1319-2442.265456>

131.	Park KS, Hwang YJ, Cho MH, Ko CW, Ha IS, Kang HG, et al. (2012) Quality of life in children with end-stage renal disease based on a PedsQL ESRD module. 1:2293-300.

132.	Pattaragarn A, Warady BA, Sabath RJ (2004) Exercise capacity in pediatric patients with end-stage renal disease. Peritoneal Dialysis International 24(3):274-80. <https://doi.org/https://dx.doi.org/10.1177/089686080402400310>

133.	Qvist E, Narhi V, Apajasalo M, Ronnholm K, Jalanko H, Almqvist F, et al. (2004) Psychosocial adjustment and quality of life after renal transplantation in early childhood. Pediatric Transplantation 8:120-5.

134.	Rahman M, Afroz S, Ali R, Hanif M (2016) Health Related Quality of Life in Children with Nephrotic Syndrome in Bangladesh. Mymensingh Medical Journal: MMJ 25:703-9.

135.	Reeve BB, Edwards LJ, Jaeger BC, Gipson DS, Thissen D, Huang IC, et al. (2016) Assessing responsiveness over time of the PROMIS pediatric health-related quality of life measures in 3 chronic diseases. Quality of Life Research 25(1 Supplement 1):47-8. <https://doi.org/https://dx.doi.org/10.1007/s11136-016-1390-7>

136.	Reynolds JM, Garralda ME, Postlethwaite RJ, Goh D (1991) Changes in psychosocial adjustment after renal transplantation. Archives of Disease in Childhood 66:508-13. <https://doi.org/10.1136/adc.66.4.508>

137.	Riar SK, Leu RM, Turner-Green TC, Rye DB, Kendrick-Allwood SR, McCracken C, et al. (2013) Restless legs syndrome in children with chronic kidney disease. Pediatric Nephrology 28:773-95. <https://doi.org/https://dx.doi.org/10.1007/s00467-013-2408-9>

138.	Roccella M, Leggio L, Parisi L, Turdo G, Testa D (2005) [The quality of life in developing age subjects with chronic renal diseases]. Minerva Pediatrica 57:119-28.

139.	Rodriguez Cuellar CI, García de la Puente S, Hernández Moraria J, Bojórquez Ochoa A, Filler G, Zaltzman Grishevich S (2019) High depression rates among pediatric renal replacement therapy patients: A cross-sectional study. Pediatr Transplant 23:e13591. <https://doi.org/10.1111/petr.13591>

140.	Rotella AAF, Nascimento RAD, Camargo MFC, Nogueira PCK (2020) Emotional Repercussions and Quality of Life in Children and Adolescents Undergoing Hemodialysis or after Kidney Transplantation. Revista Paulista de Pediatria 38:e2018221. <https://doi.org/https://dx.doi.org/10.1590/1984-0462/2020/38/2018221>

141.	Roumelioti ME, Wentz A, Schneider MF, Gerson AC, Hooper S, Benfield M, et al. (2010) Sleep and fatigue symptoms in children and adolescents with CKD: a cross-sectional analysis from the chronic kidney disease in children (CKiD) study. American Journal of Kidney Diseases 55:269-80. <https://doi.org/10.1053/j.ajkd.2009.09.021>

142.	Roussel A, Delbet J-D, Micheland L, Deschenes G, Decramer S, Ulinski T (2019) Quality of life in children with severe forms of idiopathic nephrotic syndrome in stable remission-A cross-sectional study. Acta Paediatrica 108:2267-73. <https://doi.org/https://dx.doi.org/10.1111/apa.14912>

143.	Scheuerman O, Zilber E, Davidovits M, Chodick G, Levy I (2017) Nephrologists need to play a key role in improving annual influenza vaccination rates in children with kidney disease. Acta Paediatrica 106:812-8. <https://doi.org/10.1111/apa.13763>

144.	Selewski D, Massengill S, Troost J, Wickman L, Messer K, Herreshoff E, et al. (2014) Gaining the Patient Reported Outcomes Measurement Information System (PROMIS) perspective in chronic kidney disease: a Midwest Pediatric Nephrology Consortium study. Pediatric Nephrology 29:2347-56. <https://doi.org/10.1007/s00467-014-2858-8>

145.	Selewski DT, Troost JP, Massengill SF, Gbadegesin RA, Greenbaum LA, Shatat IF, et al. (2015) The impact of disease duration on quality of life in children with nephrotic syndrome: a Midwest Pediatric Nephrology Consortium study. Pediatric Nephrology 30(9):1467-76. <https://doi.org/https://dx.doi.org/10.1007/s00467-015-3074-x>

146.	Selewski DT, Troost JP, Cummings D, Massengill SF, Gbadegesin RA, Greenbaum LA, et al. (2017) Responsiveness of the PROMIS measures to changes in disease status among pediatric nephrotic syndrome patients: A Midwest pediatric nephrology consortium study. Health and Quality of Life Outcomes Vol 15 2017, ArtID 166 15:

147.	Senses Dinc G, Cak T, Cengel Kultur E, Bilginer Y, Kul M, Topaloglu R (2019) Psychiatric morbidity and different treatment modalities in children with chronic kidney disease. Arch Pediatr 26:263-7. <https://doi.org/10.1016/j.arcped.2019.05.013>

148.	Shingde R, Guha C, van Zwieten A, Kim S, Walker A, Francis A, et al. (2024) Longitudinal associations between socioeconomic position and overall health of children with chronic kidney disease and their carers. Pediatr Nephrol 39:1533-42. <https://doi.org/10.1007/s00467-023-06236-x>

149.	Silva TMC, Alves LAC, Garrido D, Watanabe A, Mendes FM, Ciamponi AL (2019) Health and oral health-related quality of life of children and adolescents with chronic kidney disease: a cross-sectional study. Quality of Life Research 28:2481-9. <https://doi.org/https://dx.doi.org/10.1007/s11136-019-02196-8>

150.	Sinha R, Davis ID, Matsuda-Abedini M (2009) Sleep disturbances in children and adolescents with non-dialysis-dependent chronic kidney disease. Archives of Pediatrics & Adolescent Medicine 163:850-5. <https://doi.org/https://dx.doi.org/10.1001/archpediatrics.2009.149>

151.	Snethen JA, Broome ME, Kelber S, Warady BA (2004) Coping strategies utilized by adolescents with end stage renal disease. Nephrology Nursing Journal 31:41-9.

152.	Stabouli S, Gidaris D, Printza N, Dotis J, Papadimitriou E, Chrysaidou K, et al. (2019) Sleep disorders and executive function in children and adolescents with chronic kidney disease. Sleep Medicine 55:33-9. <https://doi.org/10.1016/j.sleep.2018.11.020>

153.	Stahl JL, Wightman AG, Flythe JE, Weiss NS, Hingorani SR, Stoep AV (2022) Psychiatric Diagnoses in Children With CKD Compared to the General Population. Kidney Medicine 4(6) (no pagination):<https://doi.org/https://dx.doi.org/10.1016/j.xkme.2022.100451>

154.	Sugiarto B, Dhamayanti M, Sambas DR (2019) Chronic kidney disease and emotional-behavioral disorders in adolescents. Paediatrica Indonesiana 59:321-6. <https://doi.org/10.14238/pi59.6.2019.325-30>

155.	Sundaram SS, Landgraf JM, Neighbors K, Cohn RA, Alonso EM (2007) Adolescent health-related quality of life following liver and kidney transplantation. American Journal of Transplantation 7:982-9.

156.	Tain Y-L, Lu P-C, Kuo H-C, Hsu C-N (2023) Differences in health-related quality of life in children with chronic kidney disease as reported by children and parent proxies. Pediatric Nephrology 38:519-28. <https://doi.org/10.1007/s00467-022-05621-2>

157.	Tan PSP, Mallitt KA, McCarthy HJ, Kennedy SE (2021) The impact of caring for children with posterior urethral valves. Acta Paediatrica, International Journal of Paediatrics 110(3):1025-31. <https://doi.org/https://dx.doi.org/10.1111/apa.15516>

158.	Teixeira CG, Duarte Mdo C, Prado CM, Albuquerque EC, Andrade LB (2014) Impact of chronic kidney disease on quality of life, lung function, and functional capacity. Jornal de Pediatria 90:580-6. <https://doi.org/https://dx.doi.org/10.1016/j.jped.2014.03.002>

159.	Tiwari AN, Bansal M, Manju VM, Joshi P, Sinha A, Hari P, et al. (2015) A Comparative Study to Find out the Health Related Quality of Life of Children with End Stage Renal Disease on Various Renal Replacement therapies: Self and Parental Perception. International Journal of Nursing Education 7:142-6. <https://doi.org/10.5958/0974-9357.2015.00092.6>

160.	Troost JP, Gipson DS, Carlozzi NE, Reeve BB, Nachman PH, Gbadegesin R, et al. (2019) Using PROMIS to create clinically meaningful profiles of nephrotic syndrome patients. Health Psychology 38(5):410-21. <https://doi.org/https://dx.doi.org/10.1037/hea0000679>

161.	Tsampalieros A, Blinder H, Hoey L, Momoli F, Barrowman N, Feber J, et al. (2019) Obstructive sleep apnea and hypertension in pediatric chronic kidney disease. Pediatric Nephrology 34:2361-70. <https://doi.org/10.1007/s00467-019-04295-7>

162.	Velasco J, Ferraris JR, Eymann A, Ghezzi LFR, Coccia PA, Ferraris V (2020) Health-related quality of life among siblings of kidney transplant recipients. Pediatric Transplantation 24:e13734. <https://doi.org/https://dx.doi.org/10.1111/petr.13734>

163.	Veltkamp F, Teela L, van Oers HA, Haverman L, Bouts AHM (2022) The Use of Patient-Reported Outcome Measures in Daily Clinical Practice of a Pediatric Nephrology Department. Int J Environ Res Public Health 19:<https://doi.org/10.3390/ijerph19095338>

164.	Verhofste SL, Conrad AL, Johnson RJ, Steinbach EJ, Staber JM, Harshman LA (2024) Self-concept and academic achievement in children with chronic kidney disease. Pediatr Nephrol 39:819-27. <https://doi.org/10.1007/s00467-023-06106-6>

165.	Voet M, Lucker G, Malagon I, Schaaijk NM, Cornelissen EAM, Engels Y (2024) A Mixed Method Study in Young Children Participating in Clinical Research During A Kidney Transplantation Trajectory. Prog Transplant 34:89-95. <https://doi.org/10.1177/15269248241268718>

166.	Wang Y, Liu F, Ni D, Liu J (2023) The Impact of Prolonged Home Care on Nursing Adherence and Overall Quality of Life Among Pediatric Patients Diagnosed with Nephrotic Syndrome. Altern Ther Health Med 29:144-9.

167.	Webb NJA, Woolley RL, Lambe T, Frew E, Brettell EA, Barsoum EN, et al. (2019) Long term tapering versus standard prednisolone treatment for first episode of childhood nephrotic syndrome: Phase III randomised controlled trial and economic evaluation. The BMJ 365 (no pagination):<https://doi.org/https://dx.doi.org/10.1136/bmj.l1800>

168.	Weigmann-Fasbender S, Pfeil K, Betz T, Sander A, Weis K, Tonshoff B, et al. (2020) Physical fitness and health-related quality of life in pediatric renal transplant recipients: An interventional trial with active video gaming. Pediatric Transplantation 24:e13630. <https://doi.org/https://dx.doi.org/10.1111/petr.13630>

169.	Wong C, Gerson A, Hooper SR, Matheson M, Lande M, Kupferman J, et al. (2016) Effect of elevated blood pressure on quality of life in children with chronic kidney disease. Pediatric Nephrology 31:1129-36. <https://doi.org/https://dx.doi.org/10.1007/s00467-015-3262-8>

170.	Wong G, Guha C, Mallitt KA, van Zwieten A, Khalid R, Francis A, et al. (2024) The randomized controlled trial (NAVKIDS(2)) of a patient navigator program created for children with chronic kidney disease. Kidney Int 106:736-48. <https://doi.org/10.1016/j.kint.2024.05.031>

171.	Yadav P, Nunia S, Bansal A, Sureka SK, Jena R, Ansari MS, et al. (2017) Multidimensional assessment of quality of life of children and problems of parents in Indian society after pediatric renal transplant: Beyond the conventional thoughts. Pediatric Transplantation 21:<https://doi.org/https://dx.doi.org/10.1111/petr.13001>

172.	Yang M, Chuang SY, Kennedy SE (2024) Sleep disturbances in children and adolescents after kidney transplantation. Pediatr Nephrol 39:1577-85. <https://doi.org/10.1007/s00467-023-06204-5>

173.	Youssef DM, Abo Al Fotoh MN, Elibehidy RM, Ramadan SM, Mohammad EM (2015) Nutritional knowledge following interventional educational sessions in children on regular hemodialysis. Saudi J Kidney Dis Transpl 26:250-4.

174.	Yurtdaş Depboylu G, Kaner G, Özdemir Şimşek Ö, Turan KN, Kasap Demir B (2023) Dietary acid load in children with chronic kidney disease: its association with nutritional status and health-related quality of life. Pediatr Nephrol 38:3417-26. <https://doi.org/10.1007/s00467-023-05991-1>

175.	Zelikovsky N, Schast AP, Palmer J, Meyers KEC (2008) Perceived barriers to adherence among adolescent renal transplant candidates. Pediatric Transplantation 12:300-8. <https://doi.org/https://doi.org/10.1111/j.1399-3046.2007.00886.x>
